# A multi‐layered network model identifies Akt1 as a common modulator of neurodegeneration

**DOI:** 10.15252/msb.202311801

**Published:** 2023-11-20

**Authors:** Dokyun Na, Do‐Hwan Lim, Jae‐Sang Hong, Hyang‐Mi Lee, Daeahn Cho, Myeong‐Sang Yu, Bilal Shaker, Jun Ren, Bomi Lee, Jae Gwang Song, Yuna Oh, Kyungeun Lee, Kwang‐Seok Oh, Mi Young Lee, Min‐Seok Choi, Han Saem Choi, Yang‐Hee Kim, Jennifer M Bui, Kangseok Lee, Hyung Wook Kim, Young Sik Lee, Jörg Gsponer

**Affiliations:** ^1^ Department of Biomedical Engineering Chung‐Ang University Seoul Republic of Korea; ^2^ College of Life Sciences and Biotechnology Korea University Seoul Republic of Korea; ^3^ School of Systems Biomedical Science Soongsil University Seoul Republic of Korea; ^4^ Center for Systems Biology, Massachusetts General Hospital Boston MA USA; ^5^ College of Life Sciences Sejong University Seoul Republic of Korea; ^6^ Korea Institute of Science and Technology Seoul Republic of Korea; ^7^ Information‐based Drug Research Center, Korea Research Institute of Chemical Technology Deajeon Republic of Korea; ^8^ Department of Biochemistry and Molecular Biology, Michael Smith Laboratories University of British Columbia Vancouver BC Canada; ^9^ Department of Life Science Chung‐Ang University Seoul Republic of Korea

**Keywords:** common modifier, insulin signaling pathway, multi‐layered network expansion, neurodegenerative diseases, proteostasis, Computational Biology, Molecular Biology of Disease, Neuroscience

## Abstract

The accumulation of misfolded and aggregated proteins is a hallmark of neurodegenerative proteinopathies. Although multiple genetic loci have been associated with specific neurodegenerative diseases (NDs), molecular mechanisms that may have a broader relevance for most or all proteinopathies remain poorly resolved. In this study, we developed a multi‐layered network expansion (MLnet) model to predict protein modifiers that are common to a group of diseases and, therefore, may have broader pathophysiological relevance for that group. When applied to the four NDs Alzheimer's disease (AD), Huntington's disease, and spinocerebellar ataxia types 1 and 3, we predicted multiple members of the insulin pathway, including PDK1, Akt1, InR, and sgg (GSK‐3β), as common modifiers. We validated these modifiers with the help of four *Drosophila* ND models. Further evaluation of Akt1 in human cell‐based ND models revealed that activation of Akt1 signaling by the small molecule SC79 increased cell viability in all models. Moreover, treatment of AD model mice with SC79 enhanced their long‐term memory and ameliorated dysregulated anxiety levels, which are commonly affected in AD patients. These findings validate MLnet as a valuable tool to uncover molecular pathways and proteins involved in the pathophysiology of entire disease groups and identify potential therapeutic targets that have relevance across disease boundaries. MLnet can be used for any group of diseases and is available as a web tool at http://ssbio.cau.ac.kr/software/mlnet.

## Graphical abstract

MLnet is a multi‐layered network expansion model that finds proteins with pathophysiological relevance for groups of diseases. Application to four neurodegenerative diseases predicts multiple members of the insulin pathway as common modifiers.

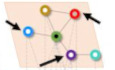

## Introduction

Neurodegenerative diseases (NDs) that cause reduced cognition and/or motor function due to extensive loss of neuronal cells affect millions of people worldwide (Erkkinen *et al*, 2018). The neuronal loss in NDs, such as Alzheimer's disease (AD), Parkinson's disease (PD), Huntington's diseases (HD) and spinocerebellar ataxias (SCAs), is believed to be caused by the abnormal accumulation of misfolded or aggregated proteins (Ross & Poirier, 2004; Chiti & Dobson, 2017; Calabrese *et al*, 2022). For all NDs, autosomal dominant disease‐causing mutations have been identified (St George‐Hyslop *et al*, 1987, 21; Campion *et al*, 1995; Roos, 2010; Klein & Westenberger, 2012). However, with the exception of diseases caused by CAG repeats (Gusella & MacDonald, 2006), familial forms with disease‐causing mutations represent a small minority of all cases of a given ND type (Bertram & Tanzi, 2005). A picture has emerged whereby multiple genetic loci are associated with specific NDs, consistent with a polygenic model in which multiple genes may interact in a synergistic or additive way to promote disease development (Ridge *et al*, 2016). Even for the case of familial NDs that are associated with a high penetrance disease‐causing mutation, genetic variation has been shown to affect the phenotype. Indeed, only between 40 and 70% of the variance in the age of onset of HD and SCA can be accounted for by the CAG repeat number in the disease‐causing allele (Wexler *et al*, 2004; Tezenas du Montcel *et al*, 2014).

As a result of these findings, significant efforts have been undertaken in the last two decades to identify genetic modifiers of NDs. Classically, genetic modifiers are studied in the context of a deterministic disease‐causing mutation and identified as those genes that affect disease severity and/or age of disease onset (Rahit & Tarailo‐Graovac, 2020). A powerful and systematic way of identifying modifier genes and pathways that impact NDs is to perform genetic screens in invertebrate models. Disease‐causing mutant genes have been used to generate various ND models in *D. melanogaster*, *C. elegans*, and *S. cerevisiae*, which have then enabled the identification of hundreds of modifiers via high‐throughput genetic screens (Fernandez‐Funez *et al*, 2000; Outeiro & Lindquist, 2003; Bilen & Bonini, 2007; van Ham *et al*, 2008, 2009; Wang *et al*, 2009; Moloney *et al*, 2010; Bloom, 2014; Shulman *et al*, 2014; Lavoy *et al*, 2018). Mapping of these modifiers has shed clear light on a broad range of processes that can modulate NDs, including RNA metabolism, protein folding, autophagy, and apoptosis, and has sparked hope for the identification of new targets for therapeutic intervention.

NDs belong to the ever‐growing group of diseases called proteinopathies (Hipp *et al*, 2014) because intracellular protein misfolding and aggregation are common to these diseases. Protein homeostasis (proteostasis) is crucial to the prevention of protein aggregation and has been demonstrated to decline with age and in proteinopathies (Balch *et al*, 2008; Labbadia & Morimoto, 2015; Hipp *et al*, 2019). Given the fact that protein misfolding and aggregation is common to proteinopathies and modifiers of one proteinopathy can influence another, e.g., a significant fraction of SCA3 modifiers in *Drosophila* had similar effects in Alzheimer models, we hypothesized that there may exist a subset of genetic modifiers that has broader relevance and may modify several or even all proteinopathies. Such common or generic modifiers may be central hubs in proteostatic control or key regulators of the cellular stress response. A bioinformatics analysis that we carried out previously on existing modifier sets revealed, however, only a small and incoherent set of modifiers that were identified in multiple ND models (Na *et al*, 2013), which may be due to the limited power and coverage of high‐throughput screens for modifiers. Therefore, we set out to develop a robust computational framework that, with the help of data integration, predicts protein modifiers common to multiple diseases. We believe that identifying modifiers is not only relevant for a better understanding of the pathophysiology of proteinopathies but may also be useful from a disease monitoring and therapeutic point of view. Common modifiers may serve as biomarkers, and monitoring their activity indicate disease risk across multiple proteinopathies. Moreover, altering the activity of the common modifiers directly or indirectly may slow disease progression or delay the age of disease onset independent of the type of proteinopathy.

**Figure 1:**
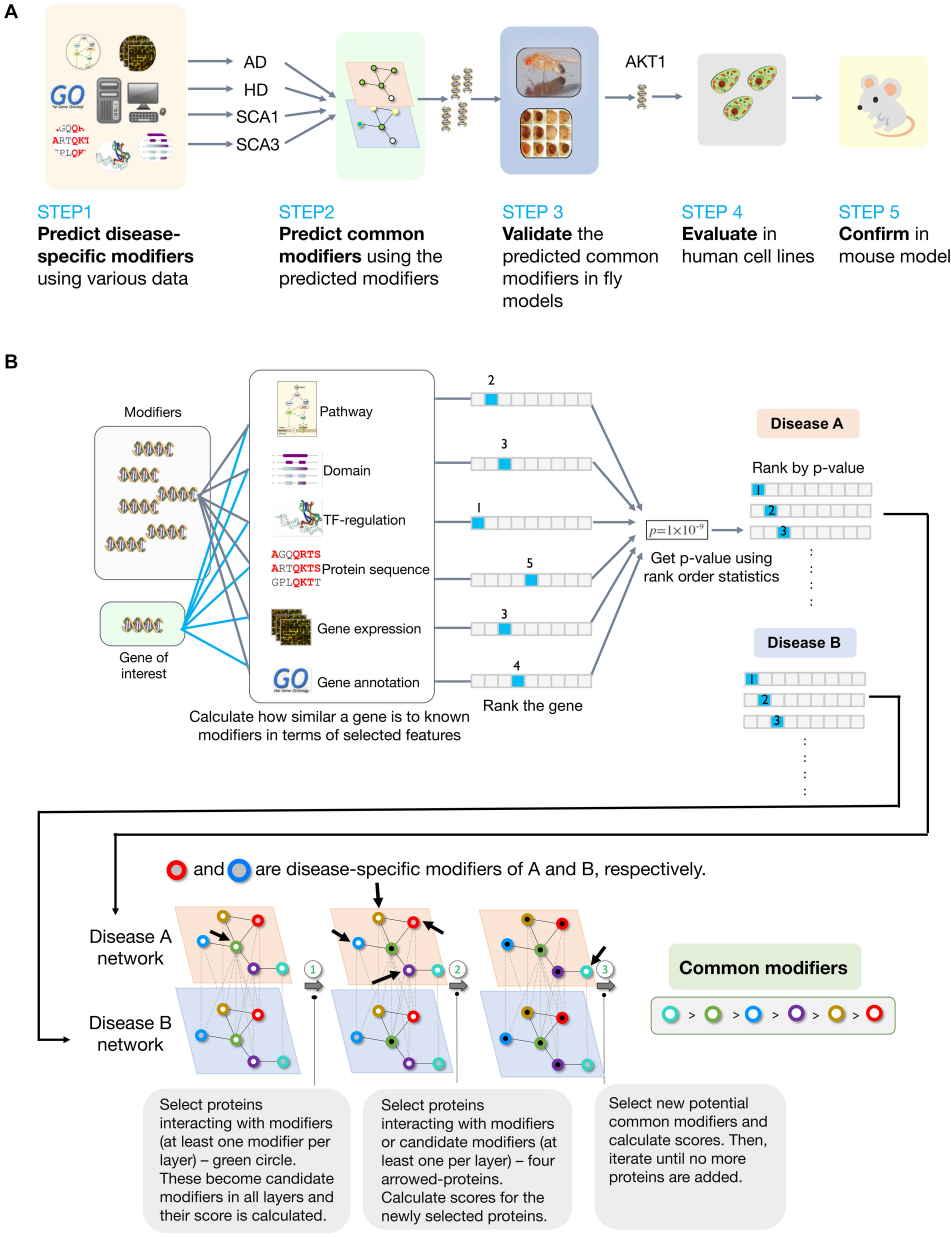
Overview of the workflow and the multi‐layered network expansion model Workflow of this study: From the identification of disease‐specific modifiers to the testing of the activation of a common modifier in an AD mouse model.Overall architecture of MLnet. It consists of two modules. In the first module (top), disease‐specific modifiers are predicted using the well‐established guilt‐by‐association principle and available annotations. In the second step, the top 100 predicted disease‐specific modifiers are used as seed proteins to predict common modifiers across multiple diseases. This prediction is done by using individual protein–protein interaction disease layers (bottom), and the idea that common protein modifiers should link disease‐specific modifiers across the different layers.

The multi‐layered network expansion model (MLnet), that we introduce here, combines transcriptome, transcription‐target relationship, protein–protein interaction (PPI), as well as meta‐data for the reliable identification of proteins that commonly affect multiple diseases. Using known AD, HD, and SCA modifiers as input, MLnet identifies many proteins in the insulin pathway as common ND modifiers. We validate predicted modifiers in *Drosophila* as well as mammalian cell models of AD, HD and SCA (Fig 1A). Following up on these results, we then show that activation of Akt1, a central hub in the insulin pathway, alleviates long‐term memory decline and ameliorates altered anxiety levels in the APP/PS1 transgenic AD mouse model. Our extensive experimental testing validates the ability of MLnet to identify generic modifier proteins that are common to a disease group.

## Results

Motivated by our hypothesis that there may exist a subset of proteins that modify the severity of multiple NDs, we aimed to develop a computational framework that allows for the identification of modifiers that are common to an entire disease group. Although computational methods for the prediction of disease‐associated genes and proteins have been developed before (Zolotareva & Kleine, 2019; Le, 2020; Chen *et al*, 2021; Ruan & Wang, 2021; Binder *et al*, 2022), no prediction methods exist, to the best of our knowledge, for the identification of proteins that commonly affect multiple diseases. Therefore, we developed the multi‐layered network expansion model, MLnet, as a general framework for the identification of modifier proteins common to a disease group and then used known ND modifiers as MLnet input in order to find proteins that may have broader relevance for proteinopathies.

### MLnet model

MLnet consists of two modules (Fig 1B). The first module predicts disease‐specific modifiers while the second integrates these predictions in multi‐layered modifier networks. The former is necessary because of imbalances in the knowledge of modifiers for different diseases, i.e., there may exist specific disease types with very few known modifiers, which will hamper any effort to identify common ones. For example, though we could find more than 100 reliable modifiers for AD and HD, only 36 and 59 modifiers for SCA1 and SCA3, respectively, were available (see Methods and Protocols, and Appendix Fig S1 for details).

Disease‐specific modifier predictions by the first module are made by the well‐established guilt‐by‐association principle and gene prioritization (Zolotareva & Kleine, 2019). Specifically, the module predicts so‐far unknown disease‐specific modifiers based on the similarity between a query gene and known genetic modifiers. The following features are used for disease‐specific modifier identification: GeneOntology (GO) annotations, InterPro domain content, gene regulation relationships (Murali *et al*, 2010), gene co‐expression data (GEO), KEGG pathway associations, and sequence similarities, which are all well‐known features successfully used in guilt‐by‐association approaches (Aerts *et al*, 2006; Zolotareva & Kleine, 2019). Since we used six different features, a gene can have up to six different scores depending on their information availability and consequently up to six different ranks. To generate a consensus list, we then integrated predicted ranks from each feature into one single *P*‐value via prioritization (Aerts *et al*, 2006). The detailed statistical calculations are explained in Methods and Protocols. From each list of disease‐specific modifiers, we then selected the proteins encoded by the top‐ranked genes as “seed” inputs for the second module.

The second module of MLnet generates disease‐specific modifier networks by mapping “seed” proteins of each disease on the PPI network of the model organism of interest. The module then identifies potential common modifiers by finding proteins that interact with or are modifiers in different disease‐specific modifier networks (Fig 1B). In the simplified example provided in Appendix Fig S2, only two layers are used. These layers are created by assuming that PPIs are identical in each disease and by mapping seeds predicted by the first module onto the individual PPI networks. In the first integration step, MLnet finds proteins that interact with at least one modifier in each layer, which in the given example is realized by the green protein because it interacts with the blue and the red seeds from the two layers. This protein is marked as a candidate common modifier across the two diseases and its score is calculated (Appendix Fig S2A and B). The common modifier score (*c*, Equation 2 in Methods and Protocols) takes into account the ranking of the connected modifiers (provided by module 1), the reliability of the protein interaction data, and the degree (number of connections) of all involved proteins. The latter is used for normalization and aims to prevent a strong bias toward interaction hubs as common modifiers. In the second step, proteins are selected that interact with at least one known modifier or candidate common modifier in each layer. In Appendix Fig S2C, four proteins (yellow, red, blue, and violet) are selected as next candidate common modifiers and their scores are calculated. In this step, the bottom right protein (cyan‐circled) is not selected, because of the constraint that proteins should interact with at least one or more known modifiers or candidate common modifiers from every layer. Finally, these steps are iterated until no more proteins are added (Appendix Fig S2D and E).

### Optimal seed number determination

We tested MLnet on its ability to predict modifiers that are common across AD, HD, SCA1, and SCA3. Specifically, we used high‐confidence modifiers identified in *Drosophila* disease models as inputs for MLnet: 113 modifiers for AD, 209 modifiers for HD, 36 modifiers for SCA1, and 59 modifiers for SCA3 (see Methods and Protocols for details and Dataset EV1 for the list of modifiers). Using these modifiers as input, MLnet outputs proteins ranked according to their likelihood of being common modifiers. Before assessing specific predictions further and validating them experimentally, we carried out several computational tests of prediction robustness.

**Figure 2:**
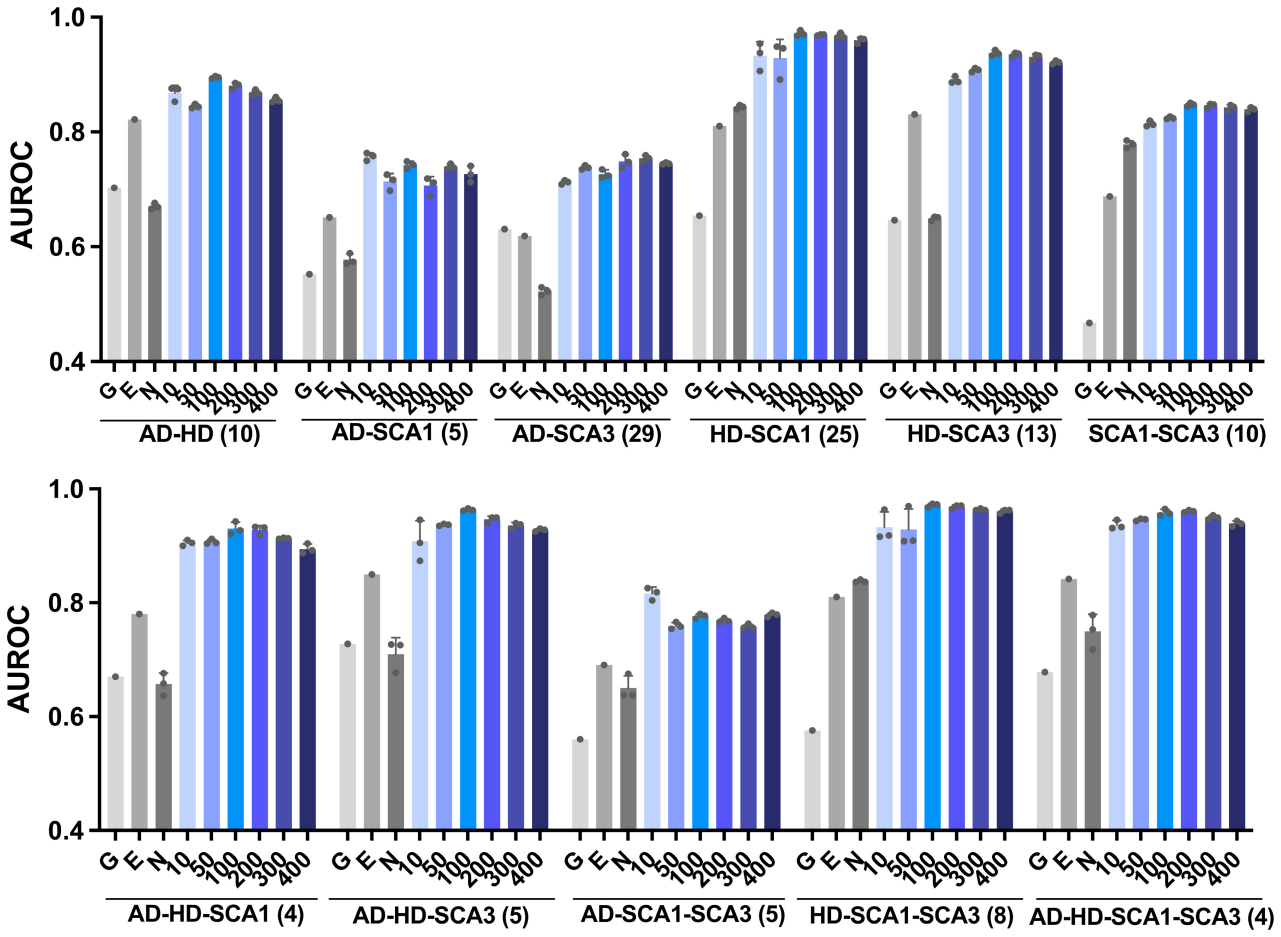
Performance evaluation of MLnet The performances of MLnet in the prediction of modifiers common to different disease groups were assessed using different numbers of seed proteins. Diseases were grouped as indicated and common modifiers for that group were predicted with MLnet. As ground truth served the intersection of high‐confidence genetic modifiers that were identified experimentally for each disease in that group. The total number of high‐confidence modifiers of each ND are: 113 for AD, 209 for HD, 36 for SCA1, and 59 for SCA3. The number of experimentally found common modifiers for each group is given in parenthesis. AUROC was calculated by leave‐one‐out‐cross‐validation and the bars are mean ± SEM (*n* = 3). The results for different numbers of seeds are shown as blue bars. As controls, we also assessed the performance of a simple gene prioritization approach (N), GeneMania (G), and Endeavour (E).

The second module of MLnet uses disease‐specific modifiers provided by the first module as input. Therefore, we first tested how variations in seed numbers affect predictions (Fig 2). We tested multiple seed numbers for their ability to identify common modifiers in a leave‐one‐out‐cross‐validation approach. In the cross‐validation, we marked experimentally identified modifiers that are common to different disease combinations as unknown and then tested how well they are predicted. Specifically, we excluded one of them from the prediction pipeline (both modules) and then calculated the rank of the excluded common modifier within the predicted common modifiers. This process was iterated for all experimentally determined common modifiers (numbers are given in parentheses in Fig 2) in order to evaluate the performance. As shown in Fig 2, using the top 100 seeds showed consistently the highest performance (Area Under the Receiver Operating Characteristics, AUROC) in predicting experimentally validated modifiers that are common to different NDs, and, thus, 100 seeds (predicted disease‐specific modifiers) were used to run the second module. The 100 seeds used to predict common modifiers across four NDs are listed in Dataset EV2.

### Robustness of MLnet output

Next, we tested the extent of MLnet output convergence toward a consistent set of proteins when including more disease layers, using different combinations of disease layers or layers with alternative modifier seeds. To this end, we predicted common modifiers using various alternative combinations of disease and seed data and compared the resulting common modifiers with those predicted when using the standard approach (Appendix Figs S3–S5). We compared the outputs by calculating Spearman's correlation coefficients of predicted common modifiers, and by counting the number of overlapping proteins within the top 100 predicted common modifiers (Dataset EV3).

In the first set of robustness test, we investigated whether the output of MLnet is dominated by modifiers from one disease layer or a pair of disease layers. In such a case, common modifiers of that pair of diseases should be more correlated with the output of MLnet than common modifiers of other disease pairs. Moreover, the top 100 common modifiers should be dominated by common modifiers of these two diseases. In other terms, leaving a disease or disease pair out should lead to a major drop in the consistency of the data. To test for this possibility, we predicted common modifiers for pairs of diseases such as AD and HD (a), and SCA1 and SCA3 (b) – or other pairings – using MLnet and then used the predicted common modifiers from (a) and (b) as seeds in the final MLnet prediction of common modifiers (c) (Appendix Fig S3A). We compared these common modifiers with those predicted by using data (seeds) from all four diseases concomitantly (d in Appendix Fig S3; the standard approach). Predictions of common modifiers for none of the disease pairs stand out or drop in terms of Spearman's correlation coefficients as well as the number of overlapping proteins within the top 100. Using randomly selected proteins as seeds in this comparison resulted in predicted common modifier lists that did not correlate at all (Spearman's correlation coefficients around 0) nor overlap.

MLnet integrates data from all disease layers concomitantly. However, it is not clear whether stepwise integration of disease layer information leads to different results. This may be the case if certain disease combinations have significantly different common modifiers. If not, the gradual integration of disease layer information should continuously increase the consistency (correlation) of the prediction. To test these ideas, we predicted common modifiers in a stepwise manner and compared correlation and overlap at each step (Appendix Fig S4A), i.e., first (a) with (d), then (b) with (d) and finally (c) with (d). Moreover, we used different orders of disease layers in this stepwise approach. In the majority of cases (8 of 12) gradual integration increased correlation in ranking and overlap of predicted common modifiers among the 100 top‐ranked proteins (Appendix Fig S4B and C). Interestingly, the overall number of overlapping proteins drops when data from the HD layer is integrated last. This suggests that HD may have quite different modifiers than the other three diseases. Nevertheless, the tests show that the majority of common modifiers that are top‐ranked by the standard data integration of MLnet (> 50%) are also found top‐ranked in an alternative integration approach where disease data is integrated gradually. Using randomly selected proteins as seeds in this comparison resulted in predicted common modifier lists that correlated negatively and had minimal overlap among the top 100 ranked proteins.

MLnet uses predicted disease‐specific modifiers from the first module as seeds. Predictions of disease‐specific modifiers are necessary for certain diseases to compensate for the lack of sufficient experimentally verified ones. We wanted to test whether using predicted disease‐specific modifiers as seeds produces results that are significantly different from those that are generated when sufficient experimentally verified disease‐specific modifiers are available and used as seeds. Therefore, we used a combination of known and predicted modifiers as seeds for MLnet and compared it to the predictions made by MLnet when using only predicted modifiers as seeds. Specifically, we used the predicted disease‐specific modifiers for SCA1 and SCA3 but known and experimentally established modifiers for AD and HD as seeds for MLnet (Appendix Fig S5A). We used this setup because the numbers of known high‐confidence SCA1 and SCA3 modifiers are lower than the optimal number of seeds for MLnet. The Spearman's correlation of the list of common modifiers predicted by this approach (b) compared to the standard one using only predicted modifiers (c) is 0.83, and the number of overlapping proteins within the top 100 is 62 (Appendix Fig S5B and C). Using randomly selected proteins as seeds in this comparison resulted in predicted common modifier lists that correlated minimally and had no overlap in the top 100 ranked proteins.

### Robustness analysis in terms of bias toward hub proteins

MLnet uses the number of interactions that a query protein has to normalize the common modifier score *c* (Equation 2) in order to reduce a potential bias toward hub proteins that are more connected in the PPI network. It needs to be stressed that the normalization aims to reduce a potential bias but not prevent hub proteins from being scored high. We tested whether alternative normalizations in module 2 would prevent any heavy bias toward interaction hubs as common modifiers and provide better prediction results. To this end, we modified the MLnet code and calculated *z*‐scores for each query protein. For the *z*‐score, we randomly selected seed proteins with the same degree as the original seed proteins (predicted disease‐specific modifiers) in 10,000 iterations (Seed randomization in Appendix Fig S6). Alternatively, we randomized the protein–protein interactions while maintaining proteins' interaction degrees (Network randomization in Appendix Fig S6). As shown in Appendix Fig S6, the integration of the alternative normalizations using seed randomization or network randomization did not improve performance when using a leave‐one‐out‐cross‐validation.

To confirm that the predicted common modifiers by the standard MLnet model were not heavily biased toward high‐degree proteins, Pearson's correlation coefficients between degrees (number of interacting partners) and ranks of top 100 and 1,000 predicted common modifiers were calculated. As shown in Appendix Fig S7, there is a low level of anti‐correlation between rank and network degree when looking at the top 1,000 predicted common modifiers. Such low level of correlation should be expected as common modifiers are likely to play a central role in the network. However, this analysis clearly shows that there is no strong bias toward high degree proteins (hubs). Importantly, there is no anti‐correlation between rank and network degree among the top 100 predicted common modifiers.

### Comparison with simple prioritization methods and added value of module 2

Finally, we assessed whether the multi‐layered approach of module 2 truly improves prediction of common modifiers. To this end, we first compared the performance of MLnet with a simple gene prioritization as employed in the first module. When we used the simple prioritization approach, the AUROCs (leave‐one‐out‐cross‐validation) of the prediction of experimentally determined common modifiers for different combinations of diseases vary between 0.5–0.8 (see bars *N* in Fig 2), but are in all cases significantly lower than the AUROCs that are achieved when using MLnet with various seed numbers (blue bars in Fig 2, *P* < 0.005).

To our knowledge, there are no computational models to predict common modifiers across multiple diseases, but there are some models that via prioritization find new genes/proteins associated with a user‐specified list of genes/proteins (Tranchevent *et al*, 2016; Zolotareva & Kleine, 2019). Our disease‐specific modifier prediction module is similar to the prioritization models, but we include a network expansion part to find common modifiers. To compare MLnet's performance further, we used GeneMania (Warde‐Farley *et al*, 2010) and Endeavour (Tranchevent *et al*, 2016). Specifically, we used them to predict disease‐associated proteins for each ND and identified the overlapping proteins as common modifiers. The resulting AUROCs are shown in Fig 2 (*G* and *E* in the graphs). The AUROCs achieved in this way are always lower than those of MLnet. To confirm the advance provided by MLnet further, we also calculated Areas under the Precision‐Recall Curve (AUPRC) for the prediction of modifiers common to different disease combinations using the optimal number of seeds (Appendix Fig S8). AUPRCs are lower than AUROCs due to the small number of common modifiers. More importantly, MLnet mostly outperforms the other methods, specifically when common modifiers across more than two diseases are predicted. In addition, AUPRCs of GeneMania and Endeavour are very low in 4–6 cases, while MLnet shows consistent performances.

To investigate the added value of module 2 in common modifier identification further, we carried out additional tests. First, we assessed whether experimentally‐identified modifiers common to different sets of disease combinations are highly ranked in the prediction lists of the other diseases (module 1) not included in the set. As shown in Dataset EV4, these experimentally established common modifiers of different disease combinations are generally not top‐ranked in the predicted lists of the other diseases. Second, we traced the disease‐specific ranks of the top 12 common modifiers predicted by MLnet, which we will discuss and experimentally validate in the following sections. Most of these genes are not ranked in top 200 of at least one of the four NDs (Dataset EV5). Thus, taking just the top ranked 200 genes of each disease and selecting common ones would not provide the result we achieve with MLnet. Finally, we color‐coded disease‐specific modifiers predicted by the first module according to their rank in the final prediction, i.e., how they are ranked as common modifiers (Appendix Fig S9A). For instance, violet‐colored disease‐specific modifiers are proteins that are found within the top 100 of the common modifiers predicted by MLnet, while red‐colored ones are found within the top 400–500 common modifiers. In a similar manner, Appendix Fig S9B shows, in color coding, how predicted common modifiers are ranked in the individual diseases. The figures show that only about 30–40% of the top 100 disease‐specific modifiers are also within the top 100 of MLnet‐predicted common modifiers. Moreover, there are no overlapping modifiers across the top 50 disease‐specific modifiers of the four diseases used here (Appendix Fig S9A). When the top 100 are considered, there are two proteins that overlap: one involved in alternative mRNA splicing and another in heat shock response.

Overall, these results demonstrate that MLnet outperforms the tested existing methods in identifying experimentally identified common modifiers of various NDs combinations. Moreover, robustness tests demonstrate convergence and superiority of the prediction by MLnet.

### Pathway analysis of predicted common modifiers

**Figure 3:**
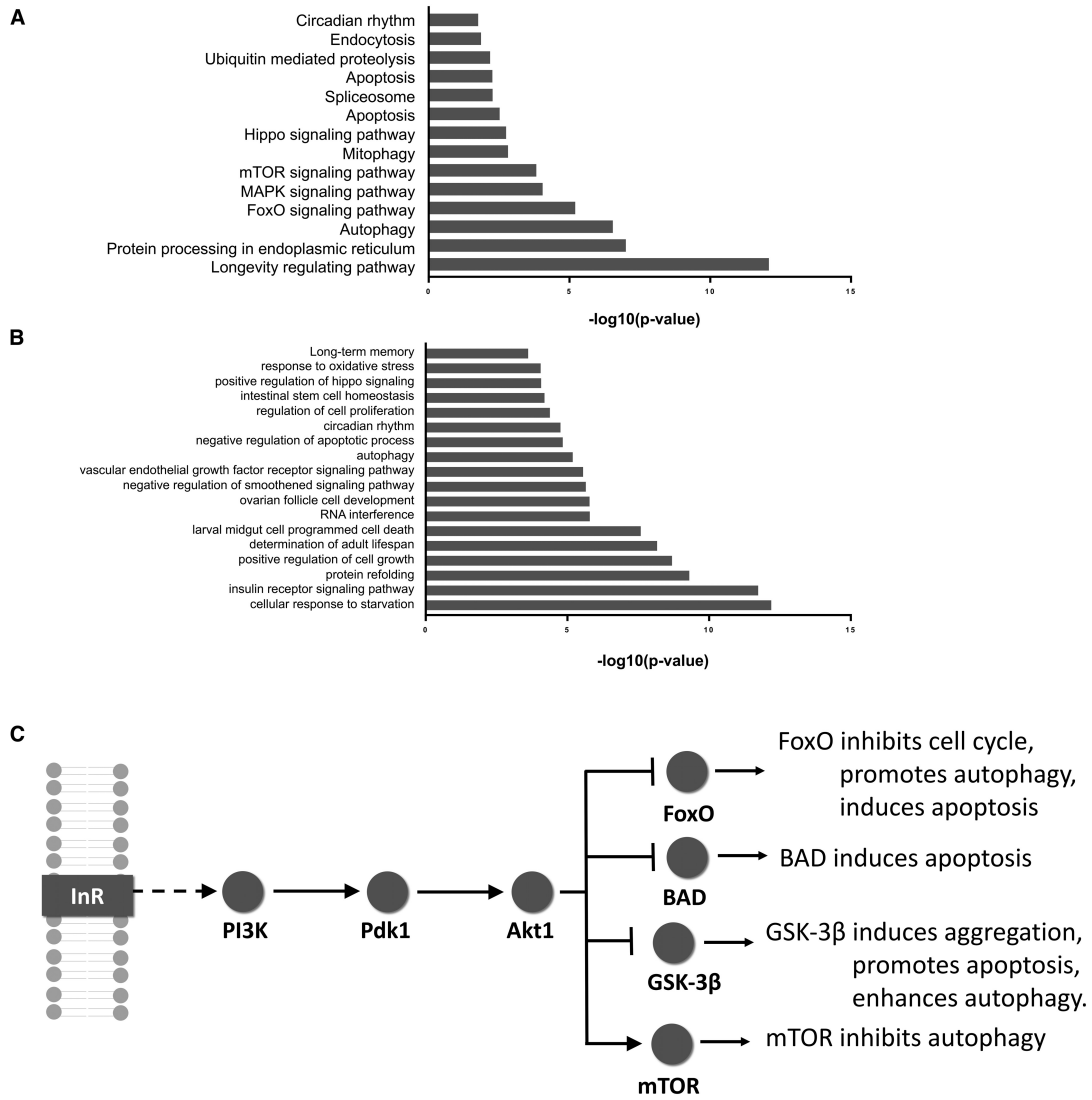
KEGG pathway and GO enrichment analyses with predicted common modifiers KEGG pathway enrichment analysis of the top 100 predicted common modifiers using DAVID (Fisher's exact test) (Sherman *et al*, 2022).GO enrichment analysis of the top 100 predicted common modifiers using DAVID (Fisher's exact test) (Sherman *et al*, 2022).A simplified schematic diagram of the insulin signaling pathway and downstream functions.

After the robustness test, we performed KEGG pathway and GO annotation enrichment analyses on the top 100 predicted common modifiers to get a better understanding of the processes these proteins are involved in. Predicted common modifiers are found with significant enrichment in the KEGG pathways of *apoptosis*, *autophagy*, and *mitophagy*, as well as the transduction pathways associated with FoxO, MAPK, mTOR, and Hippo (Fig 3A). Consistent with this KEGG pathway analysis, the GO terms of *autophagy*, and *apoptosis* but also *protein refolding* are significantly enriched among the top‐ranked common modifiers (Fig 3B). In addition, *determination of adult life span* and *long‐term memory* are also captured, terms well known to be associated with NDs (Branco *et al*, 2008; Doumanis *et al*, 2009; Zhang *et al*, 2010; Cleret de Langavant *et al*, 2013; Nuzzo *et al*, 2017; Fujikake *et al*, 2018). Most interestingly, both KEGG pathway and GO enrichment analyses find common modifiers enriched in the *insulin receptor (InR) signaling pathway* and/or its downstream effector annotations. The insulin signaling pathway plays a pivotal role in cell survival, cell growth, autophagy, and cytoskeleton organization by regulating downstream factors such as BAD, mTOR, FoxO, and GSK‐3β (Fig 3C) and has been linked to NDs in numerous studies (de la Monte & Wands, 2008; Caberlotto *et al*, 2019; Akhtar & Sah, 2020; Shaughness *et al*, 2020). Importantly, enrichment in these key annotations does not appear “*de novo*”, meaning that annotations related to downstream pathways of insulin signaling, autophagy, and apoptosis are also enriched among predicted disease‐specific modifiers (Appendix Fig S10).

To validate our specific findings, we first tested whether application of MLnet to human data would result in predicted common modifier proteins that are associated with the same pathways. Although information on human genetic modifiers is not available, there are many known ND‐associated proteins that have been discovered in genomic, proteomic, or transcriptomic analyses of ND patients. Thus, we investigated whether MLnet was able to predict common modifiers across multiple human NDs using disease‐associated proteins, not genetic modifiers. We obtained 359 AD‐associated proteins and 47 HD‐associated proteins from Neurocarta (Portales‐Casamar *et al*, 2013). Since proteins associated with SCA1 or SCA3 were not available, only modifiers common to AD and HD were predicted by MLnet, and a KEGG pathway enrichment analysis was performed on the predicted proteins. Microarray data from human brain tissue were obtained from GEO, and PPI data were filtered by using the human brain proteome obtained from the Human Protein Atlas (Sjöstedt *et al*, 2020). As shown in Appendix Fig S11, the most highly enriched pathway is the *PI3K‐Akt signaling pathway*, which is part of the insulin signaling cascade.

As the insulin signaling pathway plays a central role in metabolism and the proteins that are part of it interact with many partners, we investigated whether this pathway would automatically come up in our network‐based approach even when using genes associated with diseases not related to neurodegeneration. To this end, we collected genes related to three inflammatory diseases (gastroenteritis, hepatitis, and dermatitis) and tested for annotations enriched among the top‐ranked proteins predicted by MLnet to be common to these diseases. Specifically, we collected 265, 146 and 442 genes associated with gastroenteritis, hepatitis, and dermatitis, respectively, from Neurocarta (Portales‐Casamar *et al*, 2013) and submitted them to MLnet. Among the top 100 proteins predicted to be associated with all three inflammatory diseases, pathways related to the immune response and inflammation are significantly enriched (Appendix Fig S12 and Dataset EV6), but not insulin‐related or any other annotations found enriched among the top 100 common ND modifiers.

### Experimental validation of predicted common modifiers in *Drosophila* models

**Figure 4:**
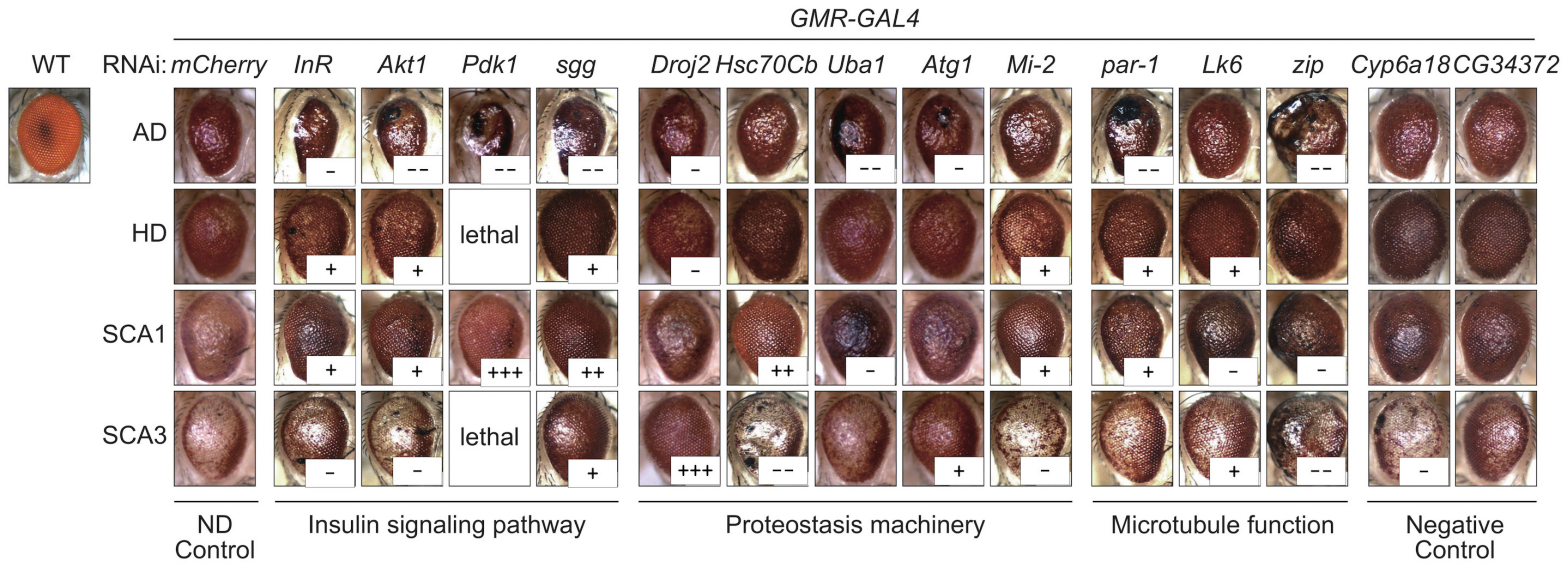
Changes in the rough eye phenotype of *Drosophila* models for AD, HD, SCA1, and SCA3 due to knockdown of predicted common modifiers Representative bright‐field microscope images of fly eyes with *GMR‐GAL4*‐driven misexpression of the ND‐causing genes, along with *GMR‐GAL4*‐driven RNAi against each of the indicated genes in which *mCherry* served as a control. Compared with the wild‐type (WT) compound eye with the ordered structure of ommatidia, flies with misexpression of individual ND‐causing genes and RNAi against *mCherry* under the control of the *GMR‐GAL4* driver had rough eyes with the variation in phenotypic severity. Suppression or enhancement of these rough eye phenotypes caused by RNAi‐mediated knockdown of the predicted common modifiers is indicated with + or −, respectively. As a negative control, two genes (*Cyp6a18* and *CG34372*) randomly selected among low‐ranked genes were also tested.Source data are available online for this figure.

To experimentally validate our findings, we tested the top 12 candidate common modifier proteins predicted by MLnet (Fig 4) with the help of *D. melanogaster* disease models (Chen *et al*, 2001; Franke *et al*, 2003). *Drosophila* compound eyes with a simple nervous system are ideal for such a test (Castedo *et al*, 2002). The severity of the eye phenotype, which is correlated with the degree of neurodegeneration, provides an easily measurable readout in this model system. To establish fly eye models for AD, HD, SCA1, and SCA3, we expressed the respective disease‐causing genes in the developing eyes using the *GMR‐GAL4* driver; *Aβ*_*1‐42*_ for AD, *Htt‐Q128* for HD, *Ataxin1‐Q82* for SCA1, and *Ataxin3‐Q78* for SCA3. As observed previously (Chan & Bonini, 2000; Nelson *et al*, 2005; Boland *et al*, 2008; Wangler *et al*, 2015), all flies with the eye‐specific expression of the disease‐causing gene showed rough eye phenotypes with some variation in the severity of the phenotype (Fig 4).

Four of the 12 tested common modifier proteins (Akt1, InR, Pdk1, and sgg (GSK3β)) changed the eye phenotypes in all four ND models when down‐regulated by RNAi. Interestingly, three of these (*Akt1*, *InR*, and *Pdk1*) are directly involved in insulin signaling and one of them (*sgg*) acts downstream of the insulin signaling pathway (Fig 3C). As a negative control, we also evaluated the effect of two randomly selected low‐ranked genes (*Cyp6a18* and *CG34372*) and found them to have little to no impact across the four *Drosophila* ND models (Fig 4). To validate the top 12 ranked proteins further, we searched the literature for evidence that supports their impact in specific NDs (Dataset EV5) and, indeed, could find evidence across diseases for many of these proteins.

Given the positive testing of all four genes related to the insulin pathway, we decided to assess the impact of another insulin pathway protein on the disease phenotypes. We evaluated Pi3K92E (PI3K in Fig 3C) because it is within the top 20 of the predicted common modifiers (Dataset EV3). Moreover, PI3K is of particular interest because it is one of the key mediators of the insulin pathway's impact on brain plasticity and neurogenesis. For instance, PI3‐kinase is essential for glutamate receptor insertion at plasma membranes during synaptic plasticity (Man *et al*, 2003). Downregulation of Pi3k92E changed the eye phenotype in all four ND models (Appendix Fig S13), confirming the significance of insulin signaling for the model phenotypes investigated here.

### Experimental validation of Akt1 in AD cell and mouse models

Motivated by these findings, we aimed to test the disease‐modifying impact of insulin signaling in mammalian models of ND. Since decreased activity of or resistance in insulin signaling is commonly found in the patients of AD, we hypothesized that activation of the insulin signaling pathway could alleviate neurodegenerative phenotypes. We chose *Akt1* as a target for insulin signaling modulation because of its central position in this pathway. Akt1 is not ranked very high in the disease‐specific modifier lists, with the exception of SCA1 (Dataset EV5), but is second in the final ranking of common modifiers due to its interaction with many proteins that are themselves disease modifiers, i.e., partners that are highly ranked in the disease‐specific modifier lists of module 1 (see Appendix Text S1, Appendix Fig S14, and Datasets EV7 and EV8 for details). Moreover, the availability of an activator of this kinase enables induction of downstream insulin signaling (Jo *et al*, 2012).

**Figure 5:**
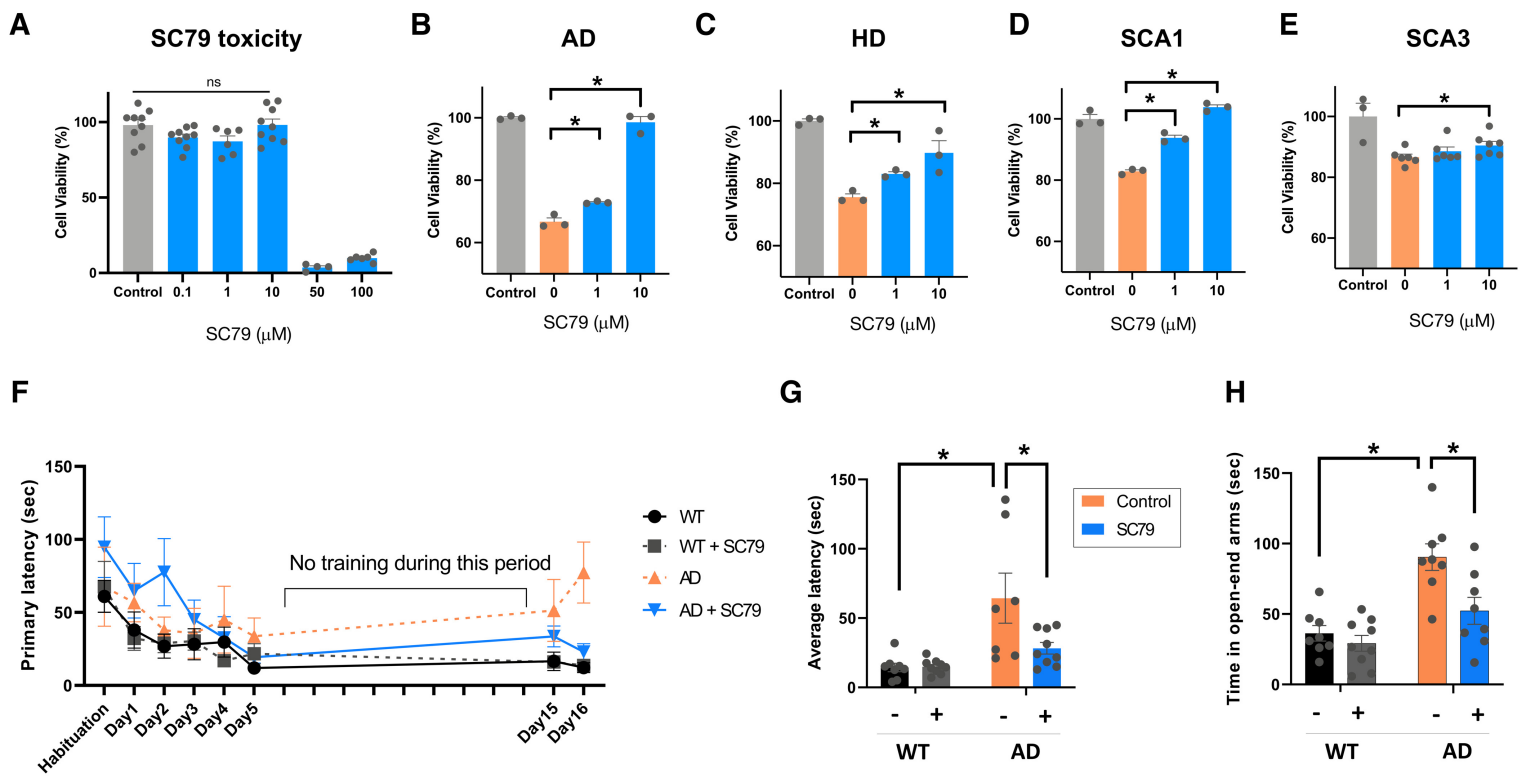
Effect of Akt1 activation on disease symptoms in mammalian disease models ACell viability at the indicated concentrations of SC79 (0.1–100 μM), an Akt1 activator, in HEK293 cells. Data are the mean and SEM of biological replicates (*n* = 4–10). * denotes *P*‐value < 0.05 (Student's *t*‐test).B–EDetermination of the alleviating effect of SC79 (1 or 10 μM) on cell death in human cell‐based models for AD (B), HD (C), SCA1 (D), and SCA3 (E). Data are the mean and SEM of biological replicates (*n* = 3–7). * denotes *P*‐value < 0.05 (Student's *t*‐test).F, GSC79 was administered to 7‐month‐old AD mice for 1 month, and their memory remedy was investigated by Barnes Maze Test. For 5 days, the time to find a target hole decreased due to learning. The mice were tested on day 15 and 16 after a blank period to investigate long‐term memory. The time to find a target hole on tested days (F) and the average of latencies on days 15 and 16 (G) are shown. Data are the mean and SEM of three biological replicates. * denotes *P*‐value < 0.05 (Student's *t*‐test).HThe dysregulated anxiety levels in AD mice were investigated using the elevated plus maze test. In (G) and (H), + and – denote the treatment and nontreatment with SC79, respectively. Bar graphs were drawn from at least three independent experiments (biological replicates) and represent mean and SEM. * denotes *P*‐value < 0.05 (Student's *t*‐test). Source data are available online for this figure.

In a first test, we constructed human cell‐based models for AD, HD, SCA1, and SCA3 by expressing ND‐causing genes (Aβ_1–42_, Htt‐Q74, Atx1‐Q52, and Atx3‐Q84) in HEK293 cells and evaluated the impact of Akt1 activation on cell phenotypes. HEK293 are generally not affected by the wild‐type forms of the disease genes. However, their viability is affected by the gene products of variants that have an increased likelihood of aggregation (e.g., polyQ repeats in HD, SCA1, and SCA3‐related genes), especially when expressed at high levels. Thus, cell viability assays with HEK293 cells have extensively been used to study disease mechanisms and test small compounds for their impact on aggregation and cell viability (Wang *et al*, 2006, 2019; Bartley *et al*, 2012; Pierzynowska *et al*, 2018; Shentu *et al*, 2019; Hart *et al*, 2022; Niu *et al*, 2022). The Akt1 activator SC79 that we used prevents the inhibitory intramolecular interaction between the plecktrin homology (PH) and catalytic domain (Warrick *et al*, 1998; Gabbouj *et al*, 2019). To activate Akt1 signaling in the disease cell models, we treated cells with 1 or 10 μM of SC79. 10 μM was the highest concentration of SC79 that showed no significant toxicity (Fig 5A). As shown in Fig 5B–E, the treatment of the cells with 10 μM of SC79 significantly increased cell viability in all models when compared with the viability of cells that were not treated with SC79.

In a second test, we investigated whether the activation of Akt1 can alleviate the symptoms in an AD mouse model. To this end, we tested the impact of SC79 in the 5xFAD mouse model. 5xFAD transgenic mice overexpress mutant human APP with the K670N, M671L, I716V, V717I familial AD mutations and human PS1 harboring the two mutations M146L and L286V. We fed cohorts of 7‐month‐old WT and 5xFAD mice with SC79 for 1 month, while control WT and 5xFAD mice received no treatment. We then used a Barnes maze test and an elevated plus‐maze test to investigate memory deficits and anxiety levels, respectively (Fig 5F–H). In the Barnes maze test, mice are exposed to the test of finding a target hole for 5 days. As shown in Fig 5F and G, the elapsed time finding the hole (primary latency) decreased due to learning and memorizing. There is no significant difference between the WT and AD groups, which suggests that the AD mice do not show any defect in short‐term memory. After a period of 10 days, during which we did not test the mice, we restarted to evaluate their ability to find the hole on days 15 and 16. Interestingly, AD mice spent significantly more time to find the hole than SC79‐treated AD mice, which found the hole as quickly as WT mice (Fig 5G).

AD patients also display dysregulated anxiety and 5xFAD transgenic mice show reduced anxiety levels (Jawhar *et al*, 2012; Belaya *et al*, 2020). Therefore, we employed an elevated plus‐maze test to examine the effect of SC79 on anxiety level. In this test, an increased residence time in open‐end arms indicates a lower level of anxiety, which the 5xFAD transgenic mice have (Fig 5H). SC79‐treated AD mice spent significantly less time in open‐end arms than non‐treated 5xFAD mice. The time spent in open‐end arms by treated AD mice is similar to the one of WT mice. Overall, these tests with 5xFAD transgenic mice suggest that activation of Akt1 in AD mice can recover long‐term memory deficits and attenuate dysregulated anxiety levels.

## Discussion

In this study, we introduce a computational model that predicts modifier proteins common to multiple related diseases. Our approach uses ideas from prioritization and network biology in order to be able to integrate genomic, transcriptomic and proteomic data. Various methods for gene and protein prioritization have been developed before (Aerts *et al*, 2006; Tranchevent *et al*, 2016; Zolotareva & Kleine, 2019; Ruan & Wang, 2021). Indeed, previous studies have integrated genotype–phenotype association data with gene annotations available in the public domain such as GO and knowledge from biomolecular interaction networks to predict new associations. The rationale for this approach is that genetic variations that are associated with a specific disease should cluster in subnetworks of physically and functionally interacting proteins (Califano *et al*, 2012). Proof‐of‐principal for this approach has been provided in the successful prediction of oncogenes for B‐cell lymphomas (Basso *et al*, 2005) or genes that increase susceptibility for obesity (Yang *et al*, 2009b). The idea of combining gene annotation and PPI information has further been exploited in prioritization methods, such as GeneMania and Endeavour, in order to predict functions and phenotypes of non‐annotated genes. However, none of the existing methods was specifically developed to identify proteins and genes that may have broader pathophysiological relevance for an entire disease group. Thus, MLnet is unique in that it creates different disease layers and identifies those proteins as common modifiers that are most connected across the different layers. The basic idea behind this approach is that common modifiers are proteins that are at the cross‐roads of pathways playing a role in the pathogenesis of the different diseases.

Multiple robustness tests that we carried out suggest that MLnet provides a consistent prediction ranking of common modifier proteins with top‐ranked proteins that reappear independent of the detail of data integration: independent from the order in which disease layer data is integrated or the use of high‐confidence, experimentally validated, or predicted disease‐specific modifiers as inputs to module 2. Disease‐specific modifiers that are highly ranked initially are not necessary among the highest ranked common modifiers and those ranked low for a specific disease may become highly ranked across diseases because the encoded proteins connect modifiers across multiple layers. For example, Akt1 was originally tested as modifier in HD and SCA1 models, but not AD and SCA3 models. In addition, the predicted ranks of Akt1 in the respective NDs were 113rd (AD), 383rd (HD), 15th (SCA1), and 260th (SCA3). Our benchmarking also demonstrates that there is no strong correlation between the network degree of a protein and its rank in the common modifier prediction. Proteins with high degree are often highly studied with links to many diseases and, therefore, appear high in rankings generated by guilt‐by‐association approaches independent of the disease in question (Gillis & Pavlidis, 2011). Most importantly, MLnet performs consistently better than classical gene prioritization and the established methods GeneMania (Warde‐Farley *et al*, 2010) and Endeavour (Tranchevent *et al*, 2016) in the identification of protein modifiers common to different ND combinations, highlighting the validity of the implemented new approach.

GO and KEGG enrichment analyses of MLnet predictions for four NDs revealed that top‐ranked modifiers are significantly associated with cellular mechanisms and pathways well‐known to modulate neurodegeneration such as autophagy and mitophagy. Most prominent among them is the insulin signaling pathway and its constituents. This finding is consistent with numerous studies from the last two decades that have demonstrated the relevance of insulin and its signaling in the pathophysiology of NDs and aging (de la Monte & Wands, 2008; van Heemst, 2010; Akintola & van Heemst, 2015; Caberlotto *et al*, 2019; Akhtar & Sah, 2020; Shaughness *et al*, 2020). To validate individual predictions, we tested the effect of the 12 top‐ranked predicted modifiers in *D. melanogaster* models for AD, HD, SCA1, and SCA3. While four of these top 12 are involved in the extended insulin pathway, five others are part of the proteostasis machinery (Droj2 and Hsc70cb are chaperones, UBA1 is the E1 ubiquitin‐activating enzyme, Agt1 an autophagy regulating Ser/Thr‐kinase, and Mi‐2 a chromatin remodeler required for heat shock gene expression), and the three remaining genes are involved in microtubule function (par‐1 and Lk6 – two kinases involved in microtubule organization – and zip (zipper) a microtubule‐binding protein). Most of these proteins have previously been found associated with ND pathology (Nishimura *et al*, 2004; Ambegaokar & Jackson, 2011; Kuo *et al*, 2013; Blázquez *et al*, 2014; Groen & Gillingwater, 2015; Zhang *et al*, 2015; Kim *et al*, 2017; Pomytkin *et al*, 2018; Shaughness *et al*, 2020; Burillo *et al*, 2021; Yakubu & Morano, 2021; Ring *et al*, 2022; Nowell *et al*, 2023). Consistent with these previous studies, we find that all 12 top‐ranked proteins, when suppressed in expression, modulate disease phenotype in at least two of the tested models. However, only proteins that are part of the insulin pathway affect phenotypes in all of the tested *D. melanogaster* disease models. Interestingly, supressing the expression of these modifiers enhances the phenotype in some ND models, while it reduces it in others. These observations are consistent with previous studies where overexpression of the same gene can have opposing effects on the phenotypic of different NDs when tested in fly models (Branco *et al*, 2008). These differences are explained by the fact that the impact of genetic modulation on ND phenotypes is highly dependent on the method and level of modulation and the complexity of the pathophysiology of the individual ND (Na *et al*, 2013). Therefore, the results of the *D. melanogaster* experiments (Fig 4) should be interpreted as evidence for the ability of the positively tested genes to act as disease modifiers rather than enhancers or suppressors.

Our finding suggests that members of the insulin pathway may have pathophysiological relevance for proteinopathies in general. This hypothesis is consistent with growing evidence in the association of insulin signaling with the pathophysiology of NDs (Blázquez *et al*, 2014; Pomytkin *et al*, 2018; Shaughness *et al*, 2020; Burillo *et al*, 2021; Nowell *et al*, 2023). Insulin and insulin‐like growth factor 1 (IGF‐1) play metabolic and neuroprotective roles in the brain (Pomytkin *et al*, 2018; Burillo *et al*, 2021). Specifically, insulin regulates glucose homeostasis and maintains energy requirements for different neuronal functions. It is vital for neuronal growth and differentiation as well as neuroprotection by modulating autophagy, mitochondrial function, ER stress, and apoptosis (Pomytkin *et al*, 2018; Burillo *et al*, 2021). Thus, dysfunction of insulin signaling makes neuronal cells vulnerable to metabolic and cellular stresses (Kim & Feldman, 2015). Moreover, the insulin signaling pathway plays key roles in brain plasticity, impacting cognitive functions such as learning and memory (Spinelli *et al*, 2019). In the hippocampus, for instance, insulin positively impacts synaptic and structural plasticity. Recently, eight genes have been associated with human adult cognitive function through rare coding variants with large effects. Four of these eight genes had previously been shown to affect insulin and the insulin pathway, although this link has been established with peripheral and not cerebral insulin (Giovannone *et al*, 2003; Hamming *et al*, 2010; Backe *et al*, 2019; González *et al*, 2022; Chen *et al*, 2023). Resistance to insulin compromises many of these regulatory aspects, which is believed to promote the development of NDs. This disease mechanism has been extensively investigated in the context of AD, where epidemiologic studies have shown that type 2 diabetes and prediabetic states of insulin resistance are risk factors for AD (Arvanitakis *et al*, 2006).

Insulin exerts its regulatory role on cellular metabolism, nutrient homeostasis and cognition mainly via the PI3K/Akt signaling cascade and the downstream effectors, FoxO and mTOR (Fig 3). FoxO impacts cell differentiation and proliferation, while mTOR regulates fatty acid and protein synthesis, as well as mitochondrial metabolism (Du & Zheng, 2021; Maiese, 2021; Querfurth & Lee, 2021). The link between insulin and mitochondrial metabolism appears to play a central role in ND pathology (Galizzi *et al*, 2021; Schell *et al*, 2021; Galizzi & Di Carlo, 2022). Indeed, mitochondrial dysfunction is a common feature of NDs, which results in ATP deficiency, oxidative stress, inflammation, and consequently apoptotic cell death (Galizzi & Di Carlo, 2022). It has been shown that reduced Akt1 signaling, which occurs in insulin resistance conditions, reduces mitochondrial respiration and increases in mitochondrial fission, eventually increasing oxidative stress (Miyamoto *et al*, 2008; Yang *et al*, 2009a). In addition to its impact on mitochondrial metabolism and stress response, altered insulin signaling can directly influence cognition. Consistent with these important roles of Akt1 in insulin signaling, our experiments demonstrate that activation of Akt1 with the small molecule SC79 increases viability of HEK293 cell expressing ND‐causing genes, and enhances long‐term memory and ameliorates dysregulated anxiety levels in AD mice.

Interestingly, insulin signaling is Janus‐faced: while it promotes cell survival, it also represses autophagy via the activation of an autophagy‐inhibiting enzyme (mTOR) and inhibition of an autophagy‐promoting enzyme (FoxO) (Fig 3). Autophagy impairment is a hallmark of NDs characterized by the cellular accumulation of protein aggregates (Subramanian *et al*, 2022). Many studies have reported that recovery of autophagy activity by boosting a metabolite (NAD) or suppressing autophagy‐inhibiting enzymes such as mTOR rescues the viability of neuronal cells (Spilman *et al*, 2010; Heras‐Sandoval *et al*, 2014; Sun *et al*, 2023). When we investigated the levels of amyloid‐β in the brain of AD mice treated with or without SC79, there were no significant changes in amyloid‐β levels whether insulin signaling was activated or not (Appendix Fig S15). As activated insulin signaling improved cell viability in *in‐vitro* assays (Fig 5B‐E), this may suggest that activation of Akt1 in our experiments may have enhanced anti‐apoptotic effects while impacting autophagy to a lesser extent. Insulin actually experts anti‐apoptotic effects via Akt1, which reduces the mitochondrial release of cytochrome c (Kang *et al*, 2003; Li *et al*, 2009). Alternatively, activation of Akt1 may be beneficial via its regulatory impact on cognitive functions (Spinelli *et al*, 2019). In any case, experiments carried out in this study are primarily meant to provide evidence for the cross‐disease relevance of MLnet‐predicted modifiers and not to elucidate the detailed molecular mechanism that confer that relevance. Moreover, although our experiments suggest a modulatory role of Akt1 for multiple NDs, it may not be an ideal target for ND treatment because of its involvement in numerous cellular processes and the fact that its enhanced activation can lead to cancerous cell transformation (Wang *et al*, 2017), which would require very close monitoring for tumorigenic effects when activated via a therapeutic agent. Other proteins in the insulin pathway such as the downstream effector GSK3β are already actively targeted for ND therapy development. GSK3β inhibitors showed positive improvement in animal models, but unfortunately failed in AD patients (Rippin & Eldar‐Finkelman, 2021; Arciniegas Ruiz & Eldar‐Finkelman, 2022). Moreover, direct insulin administration to healthy individuals and AD patients improved memory performance in small studies, but mixed results were reported for larger clinical trials (Morris & Burns, 2012; Hallschmid, 2021). It is clear that more research is required to fully understand the roles of insulin signaling in NDs and whether activation of specific elements of this signaling pathway may benefit patients.

In summary, we introduce and benchmark MLnet, as a computational model that can predict modifiers common to multiple diseases. When used on genetic modifiers of NDs, MLnet identifies the insulin signaling pathway and its constituents as potential elements that have broader relevance for proteinopathies. MLnet has limitations as it depends on third party data. Most importantly, the network expansion approach relies on accurate protein interaction data and a good coverage of the “real” network present in cells. In addition, the protein interaction network varies between cell types and tissues, which will affect MLnet's output. However, efforts are under way to map cell‐ and tissue‐specific interactomes (Huttlin *et al*, 2021; Skinnider *et al*, 2021; Holguin‐Cruz *et al*, 2022), which will provide more relevant data that can be used in the future.

## Materials and Methods

### Reagents and Tools table

**Table:** 

Reagents/Resource	Reference of source	Identifier or catalog number
***Drosophila* disease models**		
AD model	Bloomington Drosophila Stock Center	BL33769
HD model	Bloomington Drosophila Stock Center	BL33808
SCA1 model	Bloomington Drosophila Stock Center	BL39740
SCA3 model	Bloomington Drosophila Stock Center	BL8150
***Drosophila* RNAi lines**		
Droj2	Bloomington Drosophila Stock Center	BL36089
Akt1	Bloomington Drosophila Stock Center	BL31701
Atg1	Bloomington Drosophila Stock Center	BL26731
Uba1	Bloomington Drosophila Stock Center	BL36307
InR	Bloomington Drosophila Stock Center	BL31037
Par‐1	Bloomington Drosophila Stock Center	BL32410
Pdk1	Bloomington Drosophila Stock Center	BL27725
Mi‐2	Bloomington Drosophila Stock Center	BL33419
Lk6	Bloomington Drosophila Stock Center	BL28357
Hsc70Cb	Bloomington Drosophila Stock Center	BL33742
Zip	Bloomington Drosophila Stock Center	BL36727
sgg	Bloomington Drosophila Stock Center	BL35364
Cyp6a18	Bloomington Drosophila Stock Center	BL42824
CG34372	Bloomington Drosophila Stock Center	BL51472
Pi3k92E	Bloomington Drosophila Stock Center	BL61182 (AD, HD, SCA1) and BL35798 (SCA3) since BL61182 was lethal in SCA3.
***Drosophila* overexpression lines**		
Akt1	Bloomington Drosophila Stock Center	BL8191
**Plasmid DNAs**		
pEGFP‐C1‐Aβ1‐42 constructed from pCAX‐FLAG‐APP	Addgene	#30154
pEGFP‐Htt‐exon1‐Q74	Addgene	#40262
pEGFP‐Ataxin1‐52Q	Addgene	#32492
pEGFP‐C1‐Ataxin3‐Q84	Addgene	#22123
**Human cell line**		
HEK293	ATCC	CRL‐1573
**Animals**		
5xFAD mice	The Jackson Laboratory	034848‐JAX
**Reagents**		
SC79	Sigma‐Aldrich	SML0749
**Databases**		
NeuroGeM	https://neurogem.msl.ubc.ca/	
GeneOntology	http://geneontology.org/	
KEGG	https://www.genome.jp/kegg	
InterPro	https://www.ebi.ac.uk/interpro/	
Gene Regulation	http://droidb.org/	
GEO	https://www.ncbi.nlm.nih.gov/geo/	
UniProt	https://www.uniprot.org/	
STRING	https://string‐db.org/	
Neurocarta	https://gemma.msl.ubc.ca/phenotypes.html	
GeneMania	https://genemania.org/	
**Tools**		
DAVID	https://david.ncifcrf.gov/	

### Methods and Protocols

#### Data preparation

Due to the complex pathophysiology of NDs and the use of very diverse mutant genes, e.g., different lengths of polyQ in HD, SCA1, and SCA3, there are significant inconsistencies in the experimental results of different ND studies (Na *et al*, 2013). These inconsistencies are particularly prominent when studies for genetic modifier identification are compared. This fact motivated us to develop a confidence score that considered different experimental results and provided a metric of the likelihood of a gene to be a modifier or non‐modifier. The following confidence score was calculated for and assigned to each genetic modifier obtained from NeuroGeM (Na *et al*, 2013):

(1) $$S=S_{m}-S_{n}=\left(1-\Pi_{i}\left(1-r_{m,i}\right)\right)-\left(1-\Pi_{j}\left(1-r_{n,j}\right)\right)$$

*S*_*m*_ and *S*_*n*_ denote the confidence scores for being a modifier or non‐modifier, respectively. To provide a single confidence score, *S* was defined as *S* = *S*_*m*_ – *S*_*n*_. *S* is in the range of −1 to +1. A positive value of *S* indicates that the gene is likely to be a modifier, while a negative value indicates that a gene is likely to be a non‐modifier. The larger the magnitude of the score *S*, the larger is the confidence. *i* and *j* denote experiments that identify genes as modifiers or non‐modifiers, respectively, and *r*_*m*,*i*_ and *r*_*n*,*j*_ denote the reliabilities of modifier and non‐modifier, respectively. Individual results could have different *r*_*m*,*i*_ and *r*_*n*,*j*_ values depending on the specifics of experiment *i* and *j* such as the scale (primary high‐throughput screening (HTS), secondary HTS, and low‐throughput screening (LTH)) or the method used to alter gene expression (siRNA‐based interference, knockout, overexpression, etc.). Since the details of the experimental differences and their impact on the reliability of the findings are hard to quantify, we approximated reliabilities r_*m*,*i*_ and r_*n*,*j*_ by assessing how reproducible, respectively, consistent specific experimental findings are. To do so, we compared findings from different experiments with each other and assessed consistency (Appendix Fig S1). Specifically, we compared primary HTS results with LTS results. If a gene was consistently identified as a modifier or non‐modifier in both HTS and LTS, it was counted as consistent. Otherwise, it was counted as inconsistent. If LTS data was not available, HTS results were compared with secondary HTS results to calculate the consistency. Likewise, secondary HTS results were compared with LTS results to calculate the consistency of secondary HTS results. LTS results were compared with other LTS results if two or more experiments were available. As a result, we obtained the following values for both r_*m*,*i*_ and r_*n*,*j*_: 0.194 for primary HTS, 0.594 for secondary HTS, and 0.737 for LTH. The scores indicate that LTS scale experiments are more consistent than HTS (Appendix Fig S1). We calculated confidence scores (S) for all genes deposited in NeuroGeM (Na *et al*, 2013) and used the genes that had a positive confidence score as modifiers for this study. Consequently, 111 modifiers of AD, 209 modifiers of HD, 36 modifiers of SCA1, and 59 modifiers of SCA3 were obtained.

#### Prediction of disease‐specific modifiers

The statistical approach for GO, KEGG pathways, InterPro domains, and transcription regulations (transcription factor – target genes) is identical. For a query gene, we calculated the *P*‐value for the association of annotations to known genetic modifiers (those with positive confidence scores) using a hypergeometric test. We then calculated the score by summing the −log_10_ (*P*‐value) of the terms annotated to this gene. To determine the *z*‐score of a query gene, we calculated the expected score and the standard deviation for randomly selected genes in 10,000 iterations. Based on the *z*‐scores of all genes, we obtained a ranked list of potential disease‐specific modifiers.

Regarding GO, we only used GO leaf nodes from the three categories (biological processes, cellular components, and molecular functions) for the score calculation. Identical to GO annotations, we used KEGG pathway information to predict new genetic modifiers. To use protein domain information, which represents protein functions, the domains of genetic modifiers were analyzed with InterPro. The regulation relationships of transcription factors and their target genes were also used to predict new genetic modifiers. The relationships were obtained from DroID (Murali *et al*, 2010).

To use gene expression correlation as a feature, *Drosophila* microarray data was obtained from GEO (Edgar *et al*, 2002). With these data, the sum of absolute values of Pearson's correlation coefficients between a query gene and known modifiers were calculated. This summed score was then converted to a *z*‐score using the scores obtained from random models as done for GO and others.

For the use of sequence similarity, protein sequences were compared with those of known modifiers by using USearch (Edgar, 2010), a faster algorithm than Blast. The highest bit score between a query gene and known modifiers was used as a score, and then the score was also converted to a *z*‐score as done for other features.

Finally, from each dataset, we ranked genes by their *z*‐scores. We then converted the resulting six ranks to rank ratios (0 < rank ratio ≤ 1) and used the rank ratios to calculate *P*‐values based on order statistics. We prioritized potential disease‐specific genetic modifiers then by their *P*‐values.

#### Prediction of common modifiers

Once disease‐specific modifiers were predicted, we used the top *N* proteins encoded by the disease‐specific modifier genes as seeds in MLnet for the prediction of common modifiers. The optimal number of seeds (*N*) was determined before use. We selected the top *N* proteins and used their rank as their seed scores *s* = 1‐(rank‐1)/*N*. We mapped seeds for each disease on PPI networks. Specifically, we obtained PPI data from STRING (Franceschini *et al*, 2012) and constructed layered networks, each layer representing a particular disease. We then mapped disease‐specific seed modifiers onto the layered PPI networks. To illustrate this and how common modifiers are calculated in MLnet in the next steps, we provide fictitious networks in Appendix Fig S2. The score function is provided in Appendix Fig S2A, and the stepwise procedure shown in Appendix Fig S2B–E.

In the first round of MLnet calculations (Appendix Fig S2B), proteins that are connected to one or more seed modifiers from each layer are identified. In Appendix Fig S2B, there is only one protein (*p*_*1*_, green) that is linked to a seed modifier (*p*_*2*_, red) in the AD layer and another seed modifier (*p*_*3*_, blue) in the HD layer. Based on this topology, its score (*c*, common modifier score) is calculated as provided below.

(2) $$c\left(p_{k}\right)=\frac{1}{W\left(p_{k}\right)}\prod_{d}\left[\sum_{i}\frac{w\left(p_{i,k}\right)}{W\left(p_{i}\right)}\times q\left(p_{i},d\right)\right]$$

$$q\left(p_{i},d\right)=\left\{\begin{array}{c} c\left(p_{i}\right)\\ s\left(p_{i},d\right) \end{array}\begin{array}{c} \text{if}\;c\left(p_{i}\right)\;\text{is available}\\ \;\text{else if}\;s\left(p_{i},d\right)\text{is available} \end{array}\right.$$

$w\left(p_{i,k}\right)$: interaction reliability of proteins *p*_*i*_ and *p*_*k*_ (0 < *w* < 1). $w\left(p_{i,i}\right)$=1. $W\left(p_{i}\right)$: sum of interaction reliabilities of protein *p*_*i*_. $c\left(p_{i}\right)$: common modifier score of protein *p*_*i*_. $s\left(p_{i},d\right)$: seed score of protein *p*_*i*_ in disease *d*.

The red‐boxed equation in Appendix Fig S2B is for the contribution of protein *p*_*2*_ in the AD disease layer and the blue‐boxed equation is for protein *p*_*3*_ in the HD layer. An important factor in the calculation of *c*(*p*_*k*_) is *W(p*_*k*_ *)* that is the sum of interaction reliability values *w*(*p*_*i*,*k*_) for all interactions a protein has. This term is included to normalize by the number of interactions a protein has and, thereby avoid predictions heavily biased toward hub proteins, i.e., proteins with lots of interaction partners that have a higher likelihood to interact with seeds/modifiers. Interaction reliability values are obtained from the STRING database (Franceschini *et al*, 2012). In the given example, the calculation in this first step results in a score of 0.00284 for *p*_*1*_, and *p*_*1*_ is then marked as a potential common modifier in both AD and HD layers. The first step is terminated with the calculation of *p*_*1*_, since there are no more proteins that are connected to seeds in both layers. The next round of calculation begins.

In the next round, new proteins linked to a disease‐specific modifier or common modifier from each layer are identified and their scores are calculated. In Appendix Fig S2C, there are four proteins (*p*_*2*_, *p*_*3*_, *p*_*4*_, and *p*_*5*_) that are linked to a seed from each layer and/or a common modifier (by default in each later). For example, protein *p*_*2*_ (red) can be selected because it is a (seed) modifier (self‐interaction) in the AD layer and is connected to a potential common modifier (*p*_*1*_) in the HD layer (as well as to a potential common modifier (*p*_*1*_) in the AD layer). Thus, *p*_*2*_ could be another common modifier, and so its score is calculated as highlighted in Appendix Fig S2C. Similarly, other proteins (*p*_*3*_, *p*_*4*_, and *p*_*5*_) are connected to at least one modifier from each layer and their scores are calculated (black arrows in Appendix Fig S2C). In the next step, there is only one protein left that can be selected as a common modifier (*p*_*6*_) because it is linked to a common modifier (*p*_*5*_) in the AD and HD layers. Its common modifier score is then calculated as shown in Appendix Fig S2D. As there are no more proteins to be selected, the calculation round is terminated. Consequently, each protein has a score, and they are ranked by their common modifier score (Appendix Fig S2E). In the given example, though *p*_*6*_ (cyan) was last selected, it has the highest score due to its high seed score in HD. Thus, *p*_*6*_ is the most promising candidate common modifier across two diseases of our fictitious example.

#### *Drosophila* **eye models for various NDs**

To generate *Drosophila* eye models for AD, HD, SCA1, and SCA3 using the *GAL4*/*UAS* transactivation system (Brand & Perrimon, 1993), the *GMR*‐*GAL4* driver line was crossed to the *UAS*‐transgene lines being analyzed: *UAS‐Aβ42* (BL33769) for AD, *UAS‐HTT‐128Q* (BL33808) for HD, *UAS‐ATX1‐82Q* (BL39740) for SCA1 (Fernandez‐Funez *et al*, 2000), and *UAS‐MJDtr‐78Q* (BL8150) for SCA3 (Warrick *et al*, 1998). All fly lines were obtained from Bloomington *Drosophila* Stock Center (BDSC). All stocks and crosses were reared on standard cornmeal/agar media under noncrowded conditions at 25°C unless otherwise stated.

#### Evaluation of candidate modifier proteins for AD, HD, SCA1, and SCA3

To confirm MLnet‐predicted candidates for common modifier proteins, the conditional knockdown or overexpression of specific proteins was achieved with the *GAL4*/*UAS* system (Brand & Perrimon, 1993) in the *Drosophila* models for AD, HD, SCA1, and SCA3 at 25°C (for SCA3) or 29°C (for AD, HD, and SCA1), where an enhanced activity of the *GAL4*/*UAS* system is exerted (Seroude *et al*, 2002). The following RNAi lines were used in this study: *UAS‐Droj2‐RNAi* ^*TRiP*^ (BL36089), *UAS‐Akt1‐RNAi* ^*TRiP*^ (BL31701), *UAS‐Atg1‐RNAi* ^*TRiP*^ (BL26731), *UAS‐Uba1‐RNAi* ^*TRiP*^ (BL36307), *UAS‐InR‐RNAi* ^*TRiP*^ (BL31037), *UAS‐par‐1‐RNAi* ^*TRiP*^ (BL32410), *UAS‐Pdk1‐RNAi* ^*TRiP*^ (BL27725), *UAS‐Mi‐2‐RNAi* ^*TRiP*^ (BL33419), *UAS‐Lk6‐RNAi* ^*TRiP*^ (BL28357), *UAS‐Hsc70Cb‐RNAi* ^*TRiP*^ (BL33742), *UAS‐Zip‐RNAi* ^*TRiP*^ (BL36727), and *UAS‐sgg‐RNAi* ^*TRiP*^ (BL35364). Other lines included *Canton‐S* (BL64349) as a wild‐type control, and *UAS‐mCherry‐RNAi* (BL35785) and *UAS‐mCherry* (BL35787), which were used as controls for RNAi and overexpression, respectively. All fly stocks were obtained from BDSC. Flies with misexpression of *UAS‐Aβ42*, *UAS‐HTT‐128Q*, *UAS‐ATX1‐82Q*, or *UAS‐MJDtr‐78Q* under the control of the *GMR‐GAL4* driver were crossed with flies with the *UAS*‐RNAi or *UAS*‐overexpression transgene being analyzed. After anesthetizing 5‐day‐old F1 females with CO_2_, eye images were acquired using a stereomicroscope (Olympus) and a microscopic camera (Sentech America). The fly eyes were photographed under the same adjustment setting of I‐MEASURE software for capturing images. The *Drosophila* experiments were performed in a blinded manner.

#### Viability assays with HEK293 cells

HEK293 cells for viability assays were cultured in plating medium (Dulbecco's modified Eagle's medium (DMEM, Welgene, South Korea) with 10% fetal bovine serum (FBS, Welgene, South Korea) and 50 μg/ml gentamycin (Duchefa, Netherlands) in a 5% CO_2_ humidified atmosphere at 37°C.

HEK293 cells having 70–80% cell density were transiently transfected with pEGFP‐C1‐Aβ_1‐42_, pEGFP‐Htt‐exon1‐Q74 (Addgene #40262), pEGFP‐Ataxin1‐52Q (Addgene #32492), or pEGFP‐C1‐Ataxin3‐Q84 (Addgene #22123) DNA using Lipofectamine 2000 (Invitrogen, CA, USA) following the manufacturer's instructions. The pEGFP‐C1‐Aβ_1–42_ plasmid was constructed from pCAX‐FLAG‐APP (Addgene #30154).

Before drug treatment, HEK293 cells were washed with treating medium (Minimum essential medium (MEM, Gibco, MD, USA) with 1% FBS) and then treated with the indicated concentration of SC79 (0.1–100 μM) (SML0749, Sigma‐Aldrich) for 24 h.

Cell death was measured using Cell Counting Kit‐8 (CK04, Dojindo, MD, USA), which was performed according to the manufacturer's instructions. The optical density (OD) of each well was measured using a microplate reader at 450 nm (Molecular Devices, CA, USA), and the OD values were reported as % cell viability (mean ± SEM, *n* = 4–8 per group). The *in vitro* assays were performed in a blinded manner.

#### AD mice

Female and male 5xFAD mice overexpressing the mutant human APP (K670N, M671L, I716V, V717I) and PS1 (M146L and L286V) (The Jackson Laboratory, Stock No. 034848‐JAX) were treated with SC79 from 1 month before the behavioral test (7‐month‐old). Wild‐type (WT) littermates served as age‐matched control animals. Mice were separated by sex and genotype and housed in polyethylene cages (25 cm × 30 cm × 22 cm) with aspen shaving bedding (DBL, Korea), 4–5 each. They were classified into four groups (WT, WT + SC79, AD, and AD + SC79). SC79 groups were administered with SC79 dissolved in 8.5% DMSO (D2438, Sigma‐Aldrich) in corn oil 5 days per week via oral gavage (1.5 mg/kg/day in 100 μl). Body weight was measured every week. All groups were kept in standard condition (23 ± 2°C, humidity 50 ± 5%, and 12 h light/dark cycle, and light turned on from 9:00 am to 9:00 pm). Mice had *ad libitum* access to food (NIH‐31) and sterile water. All procedures were performed in accordance with Sejong University Institutional Animal Care and Use Committee.

#### Barnes maze test

Barnes maze test was performed to elucidate the effect of SC79 treatment on cognitive deficits in learning and memory as described (Patil *et al*, 2009) with slight modifications. Barnes maze apparatus is a white acrylic circular disk, 92 cm in diameter, with 20 spaced 5 cm in diameter holes. The escape chamber was placed under one of the holes, defined as the target hole. Because mice may sometimes lack entering the escape chamber motivation, mice explore the maze after finding the target hole without descending into it (Harrison *et al*, 2006). To motivate mice to enter the escape chamber, the escape chamber contained some plastic steps, aspen shavings, and six standard feeds. Other holes were closed with matte black plates. Mice were placed in a square styrofoam box (20 cm × 20 cm) covered with an opaque lid for the 20 s to specify the starting direction randomly. Each trial, lasting 3 min, was started after lifting the box. If the mice do not find the escape chamber within 3 min, they were gently guided to the escape chamber and allow the 20 s to pass before being returned to the waiting cage. The escape cage is maintained at a fixed location for all trials. On days 15^th^ and 16^th^, the mice once again received the test trial for 3 min to check long‐term retention memory.

Primary latency was defined when a mouse first poked its nose into the target hole. Mice were not tested during the period between the 6^th^ and 15^th^ day. All trials were recorded and analyzed by ANY‐maze 6.0 Software. All behavioral tests were conducted in a blinded manner and the ANY‐maze software was used to avoid any bias in behavior analysis. All behavior data are expressed as means ± SEM. Statistical significance was calculated by Student's *t*‐test.

#### Elevated plus maze test

An elevated plus maze test was performed to evaluate the anxiety‐like behavior. The apparatus was comprised of two closed arms with high walls (30 cm × 5 cm × 16 cm), two open arms with small walls (30 cm × 5 cm × 0.5 cm), and a center platform (5 cm × 5 cm). Each arm had a 10 cm end zone from the end of the arms. The apparatus was 40 cm above the floor. Mice were placed at the center facing a closed arm. Mice were allowed to move freely for 5 min. The time in the open arms was measured and recorded by Any‐maze 6.0 software. All behavioral tests were conducted in a blinded manner.

#### Amyloid‐β western blot

Half of the mouse brain samples were homogenized in RIPA buffer with a protease inhibitor cocktail (Thermo Scientific, USA). Homogenized samples were centrifuged at 20,000 × *g* for 10 min at 4°C, and the supernatant was collected and stored at −80°C until use. According to the manufacturer's instructions, protein concentrations were quantified by a Bradford assay (Bio‐Rad, USA).

Protein samples were loaded onto sodium dodecyl sulfate‐polyacrylamide gel electrophoresis (SDS‐PAGE) gel and transferred to polyvinylidene difluoride (PVDF) membranes. PVDF membranes were blocked in 5% non‐fat dry milk in tris‐buffered saline with 0.1% Tween 20 detergent (TBST). PVDF membranes were washed and incubated at 4°C overnight with primary anti‐Amyloid‐β antibody (BioLegend, USA) and anti‐β‐actin antibody (Cell signaling, USA). Membranes were washed and incubated with horseradish peroxidase (HRP)‐conjugated secondary antibodies (Abcam, USA) for 2 h at room temperature. Protein bands were detected by using Fusion Solo (Vilber Lourmat, France) with Miracle‐Star™ Western Blot Detection System (iNtRON Bio, Korea). The intensity of the protein bands was normalized against β‐actin and quantified using the ImageJ software (National Institutes of Health, USA).

## Author contributions

**Jörg Gsponer:** Conceptualization; software; formal analysis; supervision; funding acquisition; investigation; methodology; writing – original draft; project administration; writing – review and editing. **Dokyun Na:** Conceptualization; software; formal analysis; funding acquisition; investigation; methodology; writing – original draft; writing – review and editing. **Do‐Hwan Lim:** Conceptualization; formal analysis; investigation; visualization; methodology; writing – original draft. **Jae‐Sang Hong:** Formal analysis; investigation; visualization; methodology; writing – original draft. **Hyang‐Mi Lee:** Resources; data curation; software; formal analysis; investigation; visualization. **Daeahn Cho:** Software; formal analysis. **Myeong-Sang Yu:** Software; formal analysis; investigation; visualization. **Bilal Shaker:** Software; formal analysis; visualization. **Jun Ren:** Resources; software; formal analysis; visualization. **Bomi Lee:** Formal analysis; investigation; visualization. **Jae Gwang Song:** Formal analysis; investigation; visualization. **Yuna Oh:** Formal analysis; investigation; visualization. **Kyungeun Lee:** Formal analysis; investigation; visualization. **Kwang‐Seok Oh:** Formal analysis; investigation; visualization. **Mi Young Lee:** Formal analysis; investigation; visualization. **Min‐Seok Choi:** Formal analysis; investigation; visualization. **Han Saem Choi:** Formal analysis; investigation; visualization. **Yang‐Hee Kim:** Formal analysis; investigation; visualization. **Jennifer M Bui:** Conceptualization; formal analysis; investigation; methodology. **Kangseok Lee:** Resources; formal analysis; investigation. **Hyung Wook Kim:** Data curation; formal analysis; investigation; visualization; writing – original draft. **Young Sik Lee:** Formal analysis; supervision; funding acquisition; investigation; writing – original draft; writing – review and editing.

## Disclosure and competing interests statement

The authors declare that they have no conflict of interest.

## Supporting information



Appendix

Dataset EV1

Dataset EV2

Dataset EV3

Dataset EV4

Dataset EV5

Dataset EV6

Dataset EV7

Dataset EV8

Source Data for Appendix

Source Data for Figure 4

Source Data for Figure 5

## Data Availability

Due to the complexity of the codes and dependent dataset, the developed model was implemented as a web server for easy access and use, http://ssbio.cau.ac.kr/software/mlnet. Full source codes and datasets are available at http://ssbio.cau.ac.kr/software/mlnet and source codes are also available at https://github.com/blisszen/mlnet.
