# Supplementary material for: A multi‐layered network model identifies Akt1 as a common modulator of neurodegeneration
Source: Mol Syst Biol. 2023 Nov 20;19(12):e11801. doi: 10.15252/msb.202311801 (PMC10698508; doi:10.15252/msb.202311801)
Supplement: Supplementary file 1 — Appendix [file MSB-19-e11801-s002.pdf]

Appendix

Genome-wide multi-layered network model identifies Akt1 as a common disease modulator across multiple neurodegenerative diseases

Dokyun Na, Do-Hwan Lim, Jae-Sang Hong, Hyang-Mi Lee, Daeahn Cho, Myung-Sang Yu, Bilal Shaker, Jun Ren, Bomi Lee, Jae Gwang Song, Yuna Oh, Kyungeun Lee, Kwang-Seok Oh, Mi Young Lee, Min-Seok Choi, Han Saem Choi, Yang-Hee Kim, Jennifer M. Bui, Kangseok Lee Hyung-Wook Kim, Young Sik Lee, and Jörg Gsponer

Appendix Text

**Appendix Text S1:**	Stepwise analysis of how Akt1 acquires a top rank	………	3

Appendix Figures

**Appendix Figure S1:**	Consistency in experimental results	………	4
**Appendix Figure S2:**	An example of MLnet's score calculation	………	5
**Appendix Figure S3:**	Robustness analysis of MLnet	………	6
**Appendix Figure S4:**	Robustness analysis of MLnet	………	7
**Appendix Figure S5:**	Robustness analysis of MLnet	………	8
**Appendix Figure S6:**	Performances of MLnet with and without randomization	………	9
**Appendix Figure S7:**	Pearson's correlation coefficients between rank and degree of predicted top 100 and top 1,000 common modifiers	………	10
**Appendix Figure S8:**	Comparison of MLnet with other methods using Areas under the Precision-Recall Curves		11
**Appendix Figure S9:**	Rank comparison of predicted disease-specific modifiers and common modifiers	………	12
**Appendix Figure S10:**	Pathway enrichment analysis results for the top 100 predicted disease-specific modifiers.	………	13
**Appendix Figure S11:**	Pathway enrichment analysis result for predicted common modifiers of human AD and HD	………	14
**Appendix Figure S12:**	Pathway enrichment analysis results for genes predicted by MLnet to be commonly associated with gastroenteritis, hepatitis and dermatitis	………	15
**Appendix Figure S13:**	Effect of Pi3K92E knockdown on *Drosophila* eye phenotype in four ND models.		16
**Appendix Figure S14:**	Histogram of interaction partner ranks.	………	17
**Appendix Figure S15:**	Amyloid-ß in SC79-treated and non-treated AD mouse brain samples	………	18

Appendix Text 1: ***Stepwise analysis of how Akt1 acquires a top rank***

We selected Akt1 as a target for insulin signaling modulation and use it here as a showcase to demonstrate how this protein reaches a top rank.

Akt1 has been tested as modifier in models of HD and SCA1, but not of AD and SCA3 (**Table EV7**). Akt1's assigned confidence score for the experimental results in HD and SCA1 models are 0.60 and 0.72, respectively. The high confidence scores compared with other modifiers (half of HD modifiers have a confidence score lower than 0.2) are due to the fact that Akt1 was identified as a modifier in several low-throughput experiments.

Among disease-specific modifiers predicted by the first module of MLnet, Akt1 ranks only within the top 100 in SCA1 (**Table EV8**). However, Akt1 ranks second among common modifiers (**Table EV3**), which shows that the multi-layered networks approach of the second module of MLnet significantly changes its rank. For comparison, we provide two additional *Drosophila* genes (CG7470 and CG10749) that are ranked very low in the individual diseases (not tested experimentally), don't get a high common modifier ranking, but have a similar number of protein interaction partners as Akt1 in the network (**Table EV8**). In MLnet, the common modifier score (and, thus, the rank) of a protein generated in module 2 depends on its own seed score (from module 1) as well as the seed scores (from module 1) and the updated common modifier score of its interaction partners in the network (see **Appendix Figure Fig S2**). As Akt1 acquires a high common modifier score but is initially not top-ranked in individual diseases, it can be assumed that it gets this high final score because the scores of Akt1's interaction partners are high across the different diseases, higher than those of proteins that don't get pushed to the top of the common modifier list by module 2. Therefore, we investigated the distribution of the ranks of interacting partners of Akt1 and the two *Drosophila* genes CG7470 and CG10749 that are not ranked high among predicted common modifiers (they are ranked 1102 and 4827, respectively). **Appendix Figure Fig S14** shows the histogram of the ranks of interaction partner proteins of these proteins in the individual diseases. As only top 100 proteins from each disease get a seed score, only the number of interaction partners that are part of these 100 seeds are shown. As can be seen from **Appendix Figure Fig S14**, Akt1 has, consistently across all diseases, more interaction partners that are top ranked, i.e., that are part of the 100 seed proteins. Consequently, Akt1's rank in the list of predicted common modifiers is high in contrast to two other genes that have a similar number of interaction partners as Akt1 but much fewer interaction partners that are top ranked in individual disease and used as seeds.

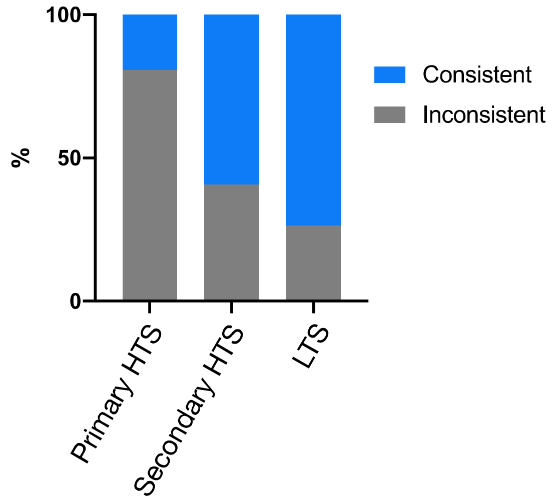

Appendix Figure S1. Consistency in experimental results.

Percentages (y-axis) denote how many genes were consistently identified as a modifier or non-modifier when compared with other results obtained from smaller-scale experiments. The confidence scores of primary HTS, secondary HTS, and LTS are 0.194, 0.594, and 0.737, respectively.

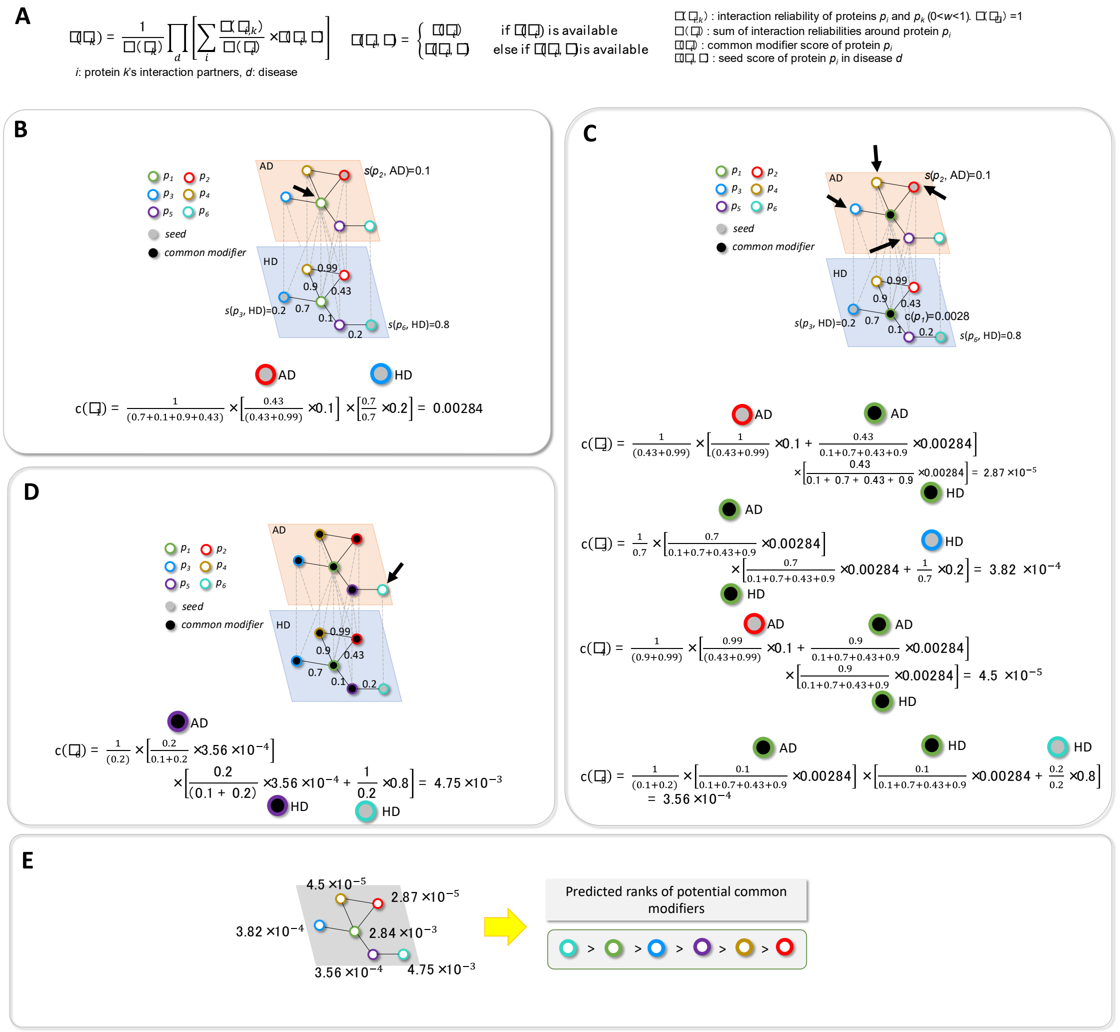

Appendix Figure S2. An example of MLnet's score calculation

A Common modifier score formula.

B The first step in the calculation of a common modifier score. Protein interaction networks of two disease layers (AD highlighted in light red and HD in light blue) are shown. Different proteins, represented as nodes, are color-coded and connected by edges with interaction partners. The reliability values $w(p_{i,k})$ for all interactions are provided in the HD network (but are identical for the AD network). Seed nodes are additionally colored in grey (p_2 for AD and p_3 for HD). The protein (p_1), for which the common modifier score is calculated in the first step, is identified by the black arrow. p_1 is chosen because it is the only node that is connected with a seed in each disease layer. The contributions of each protein to the calculation are highlighted in the color code of that protein. Indicated is also the disease (layer) from which the contributions originate.

C-D. Second and third steps of the common modifier selection and score calculation. Procedures are explained as for A.

E The final common modifier scores of the proteins.

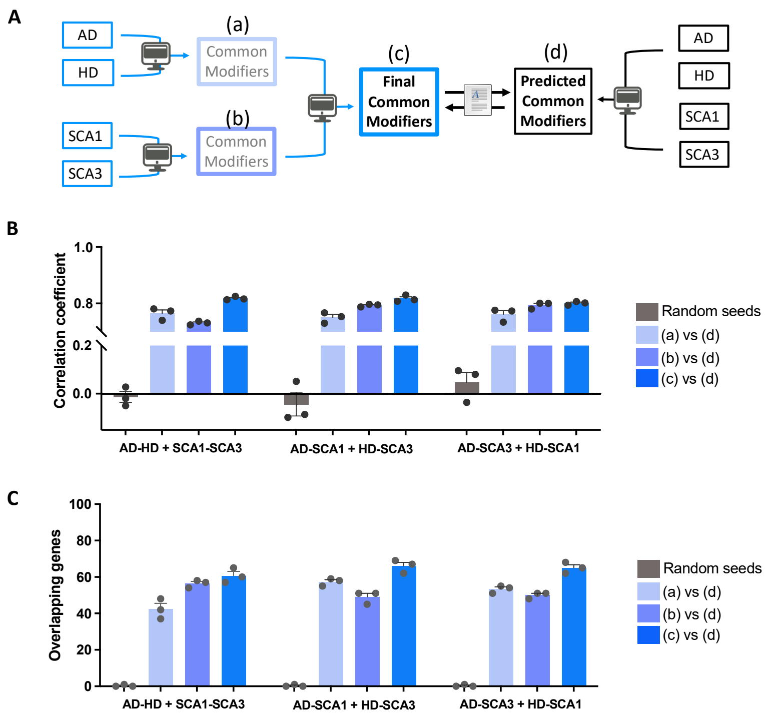

Appendix Figure S3. Robustness analysis of MLnet

A Modifiers common to pairs of diseases were predicted first with MLnet (e.g., for AD and HD), which were then used as seeds to predict common modifiers of all diseases with MLnet. The computer picture indicates when MLnet was used. The intermediate predictions (a) and (b) as well as the combined ones (c) were then compared with the standard approach (d).

B The Spearman's correlation coefficients between the common modifiers predicted by the different approaches.

C The number of common modifiers found within the top 100 predicted common modifiers by the different approaches. The bars represent an average with a standard error ($n = 3$).

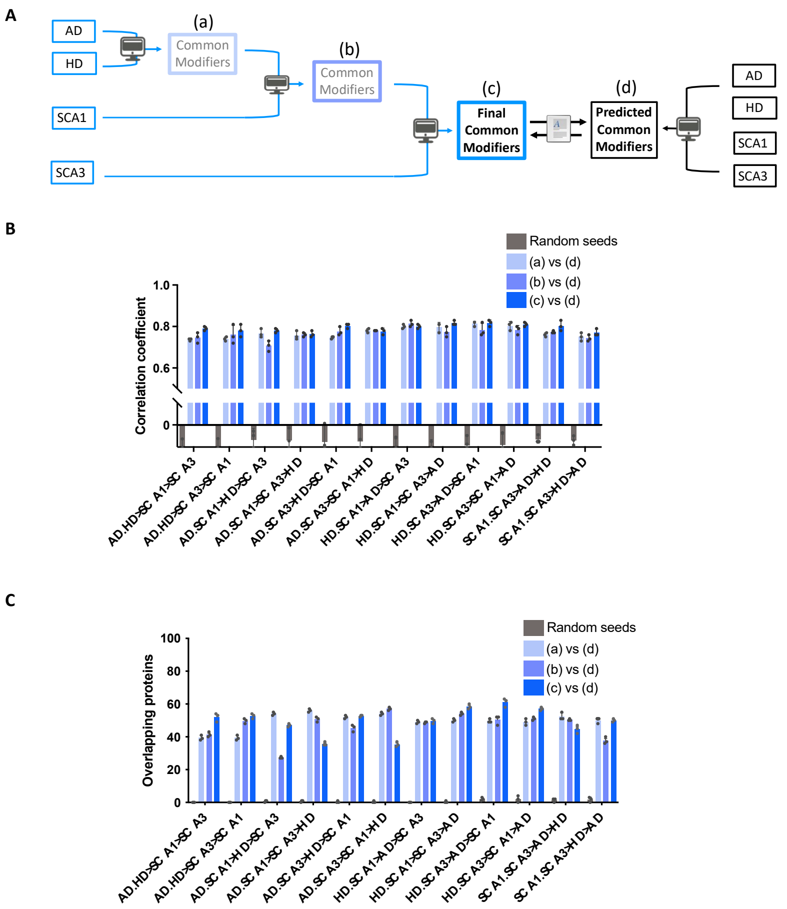

Appendix Figure S4. Robustness analysis of MLnet

A Common modifiers were predicted with MLnet via gradual disease layer integration. The computer picture indicates when MLnet was used. The intermediate predictions (a) and (b) as well as the combined ones (c) were then compared with the standard approach (d).

B The Spearman's correlation coefficients between the common modifiers predicted along the data integration (a, b, and c) and the standard MLnet method (d). Disease layer information was integrated in different orders (provided on the x-axis).

C The number of common modifiers found within the top 100 predicted common modifiers by the data integration approaches. The bars represent an average with standard error ($n = 3$).

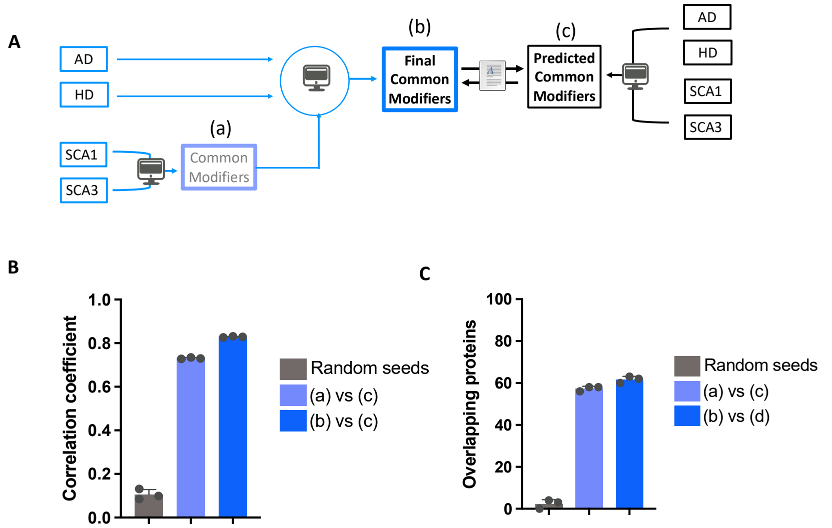

Appendix Figure S5. Robustness analysis of MLnet

A Common modifiers were predicted by MLnet using as seeds known AD and HD-specific modifiers as well as common modifiers predicted with MLnet for SCA1 and SCA3. The final common modifiers predicted with this approach (b) were compared with the common modifiers directly predicted with the four diseases using MLnet's standard method (c). For completeness, common modifiers predicted for SCA1 and SCA3 (a) were also compared with (c).

B The Spearman's correlation coefficients between the common modifiers predicted by the different approaches.

C The number of overlapping common modifiers found within the top 100 predicted common modifiers by the different approaches. The bars represent an average with standard error (n = 3).

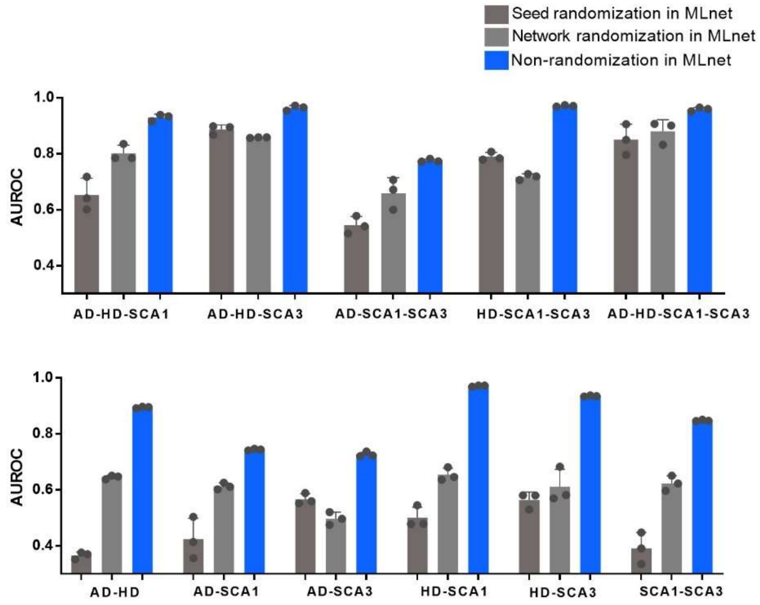

Appendix Figure S6. Performances of MLnet with and without randomization.

The AUROCs of the standard MLnet model (non-randomization), a model in which randomly chosen seeds were used to calculate z-scores (seed randomization), and a model in which randomized networks were used to calculate z-scores (network randomization). For seed randomization approach, seed proteins with the same interaction degree as the query seeds were randomly selected, and z-scores of all proteins in the network were calculated with 10,000 iterations. For network randomization approach, protein-protein interactions were newly linked while maintaining their interaction degrees and z-scores of all proteins in the network were calculated with 10,000 iterations. AUROC was calculated by leave-one-out-cross-validation and the bars are mean ± standard error (n = 3).

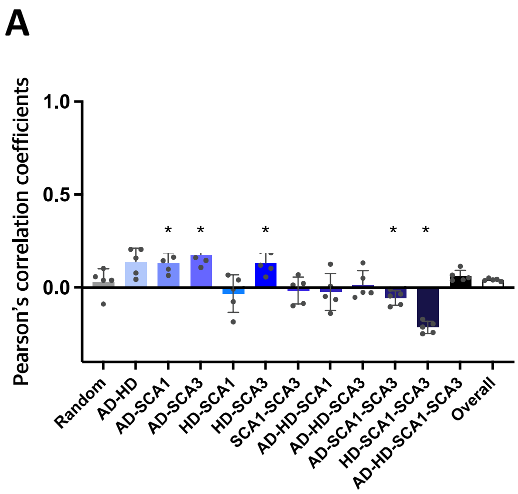

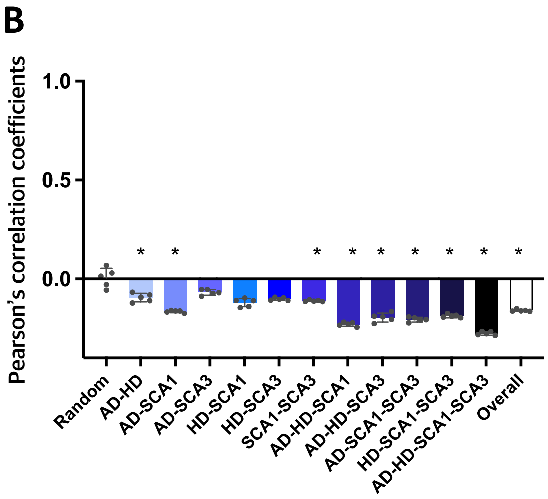

Appendix Figure S7. Pearson's correlation coefficients between rank and degree of predicted top 100 and top 1,000 common modifiers.

A-B Pearson's correlation coefficients of the top 100 (**A**) and 1,000 (**B**) predicted common modifiers in terms of their rank and degree (number of interacting partners). The correlation coefficients were calculated in various pairs of diseases. (Random) As a reference, 100 and 1,000 proteins were randomly selected from the PPI network, and their correlation was calculated. (Overall) The averaged correlations of predicted common modifiers from the various pairs of diseases. The bars are mean ± standard error ($n = 5$). Asterisk (*) denotes p-value < 0.001 when compared with randomly selected proteins.

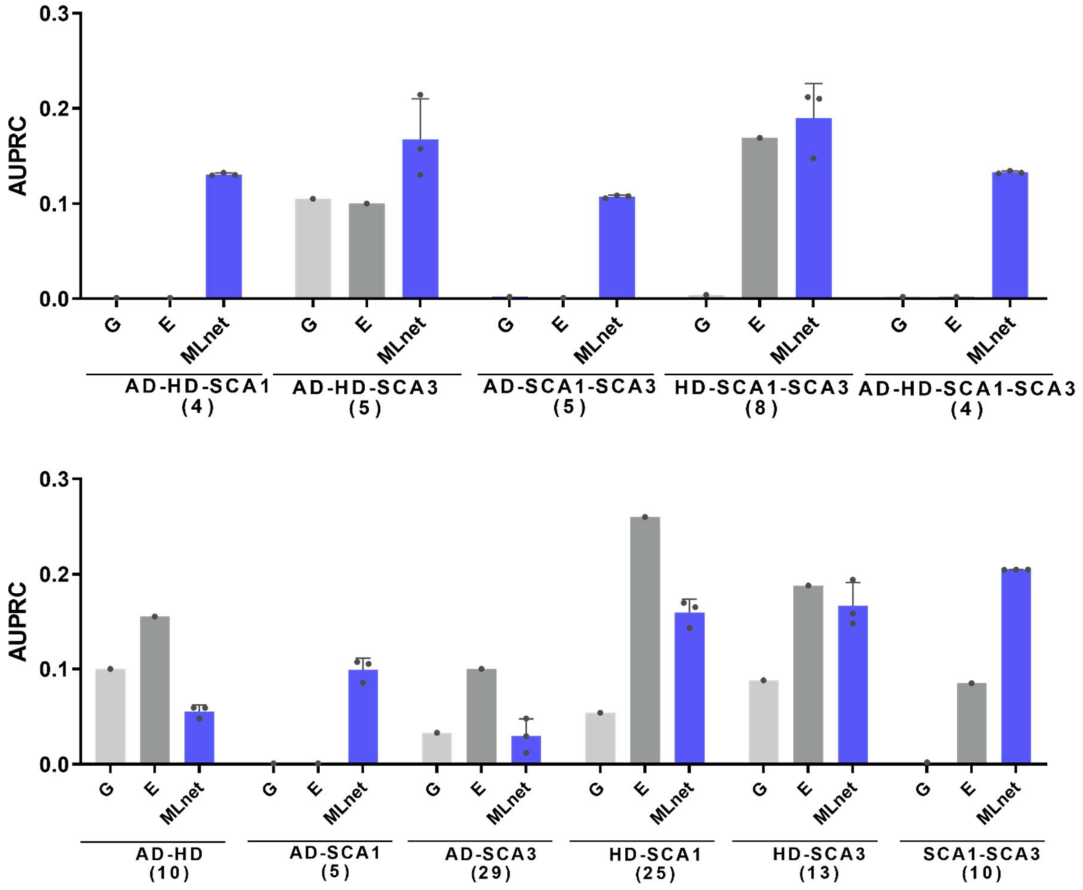

Appendix Figure S8. Comparison of MLnet with other methods using Areas under the Precision-Recall Curve

AUPRCs of MLnet, GeneMania (G), and Endeavour (E). The performances of MLnet in the prediction of modifiers common to different disease groups was assessed using the optimal seed number (100). The number of experimentally identified common modifiers for each group is given in parenthesis.

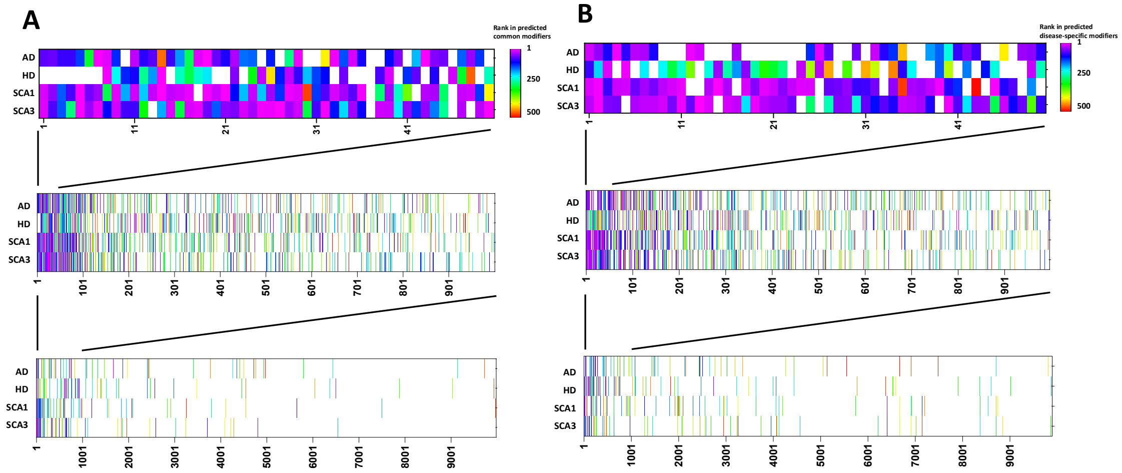

Appendix Figure S9. Rank comparison of predicted disease-specific modifiers and common modifiers.

A Disease-specific modifiers listed from left to right by their predicted ranks (*x*-axis). Color denotes the rank of these disease-specific modifiers within the top 500 of MLnet-predicted common modifiers. White color denotes that a protein was outside the top 500.

B Common modifiers listed by their predicted ranks (*x*-axis). Color denotes the rank of these common modifiers within the top 500 disease-specific modifiers. White color denotes that a protein was outside the top 500.

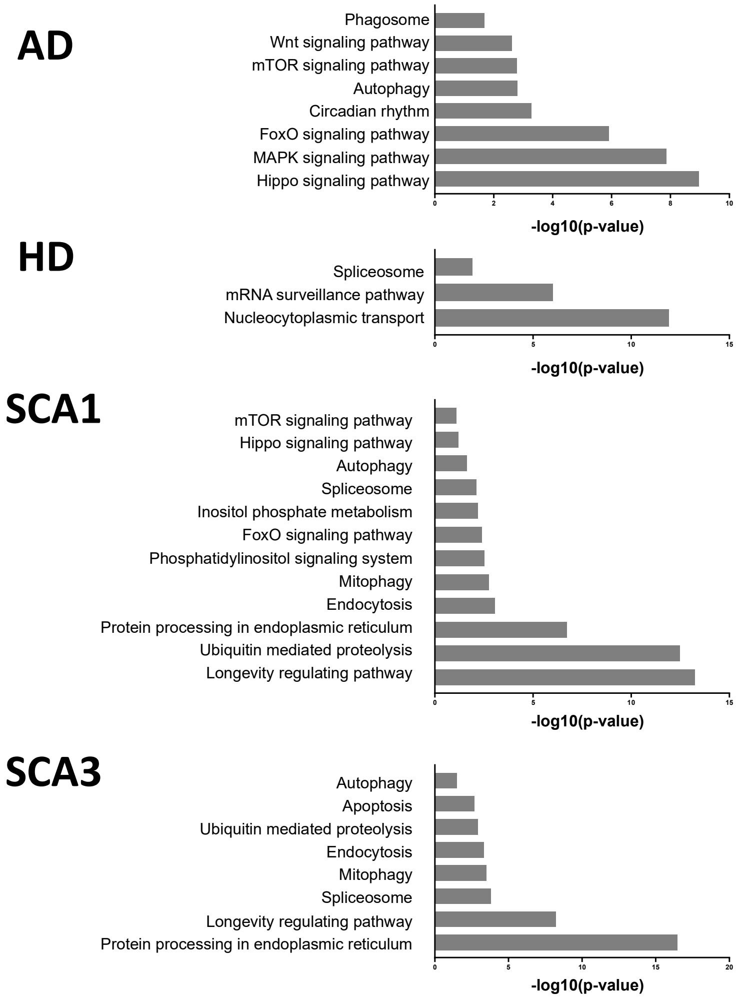

Appendix Figure S10. Pathway enrichment analysis results for the top 100 predicted disease-specific modifiers. Results are displayed as in Figure 3.

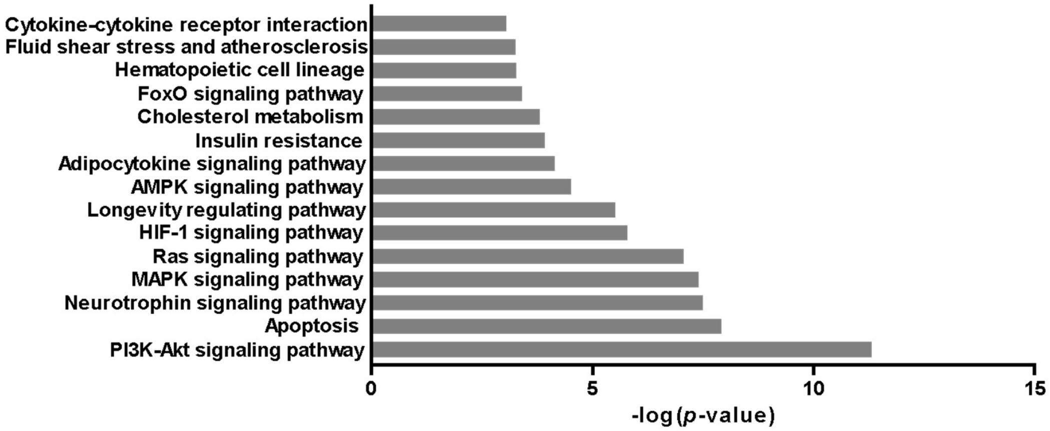

Appendix Figure S11. Pathway enrichment analysis results for predicted common modifiers of human AD and HD. Results are displayed as in Figure 3.

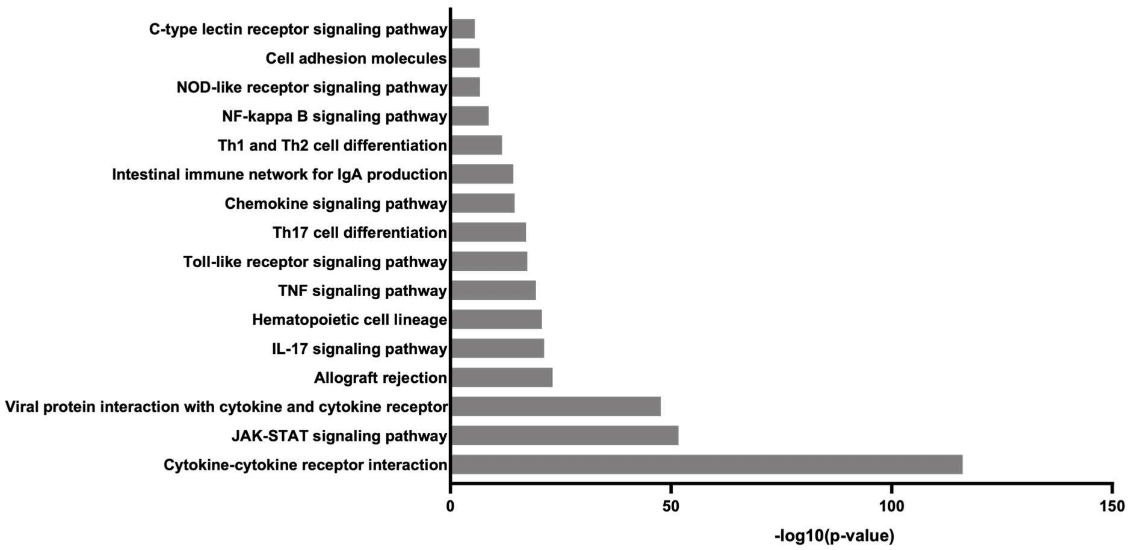

Appendix Figure S12. Pathway enrichment analysis results for genes predicted by MLnet to be commonly associated with gastroenteritis, hepatitis and dermatitis

Results are displayed as in Figure 3. The disease-related genes and predicted common ones are listed in **Table S6**.

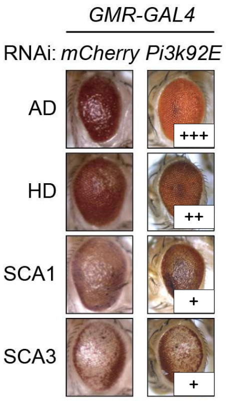

Appendix Figure S13. Effect of Pi3K92E knockdown on *Drosophila* eye phenotype in four ND models. Results are shown as in Figure 4.

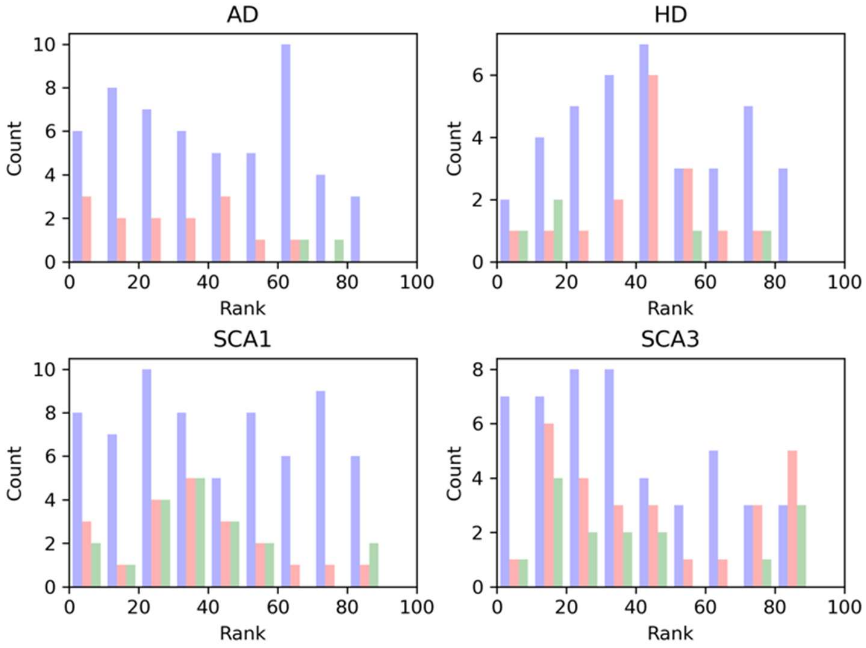

Appendix Figure S14. **Histogram of interaction partner ranks.**
Interaction partners of Akt1 (blue), CG7470 (red), and CG10749 (green) in the predicted disease-specific modifier lists are binned according to their ranks and the count per bin is provided. Ten bins are used for the top 100 ranks of each ND.

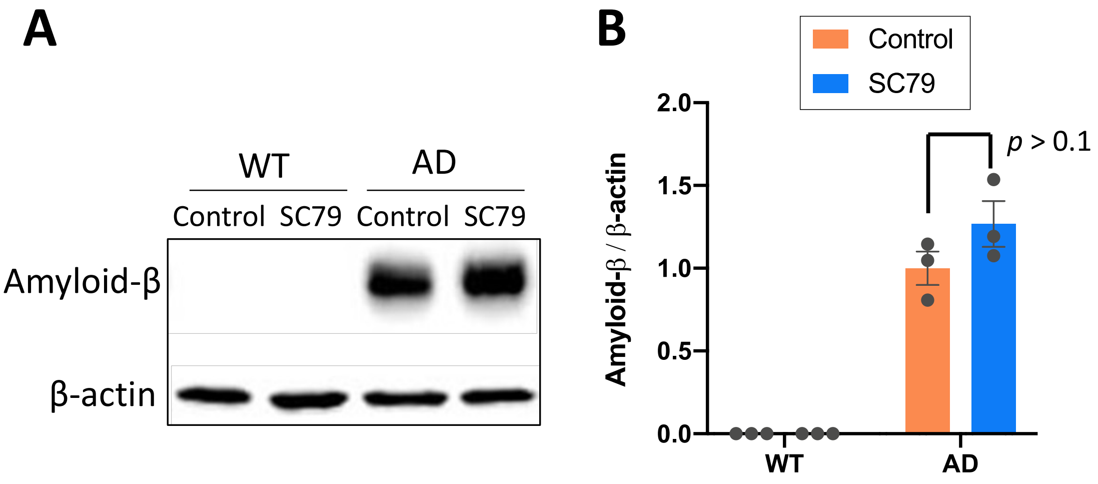

Appendix Figure S15. Amyloid-β in SC79-treated and non-treated AD mouse brain samples

A Western blot image of amyloid-β in mice brain.

B Quantified ratio of amyloid-β/β-actin. Student's *t*-test.
