# Supplementary figures and images for: A multi‐layered network model identifies Akt1 as a common modulator of neurodegeneration
Source: Mol Syst Biol. 2023 Nov 20;19(12):e11801. doi: 10.15252/msb.202311801 (PMC10698508; doi:10.15252/msb.202311801)

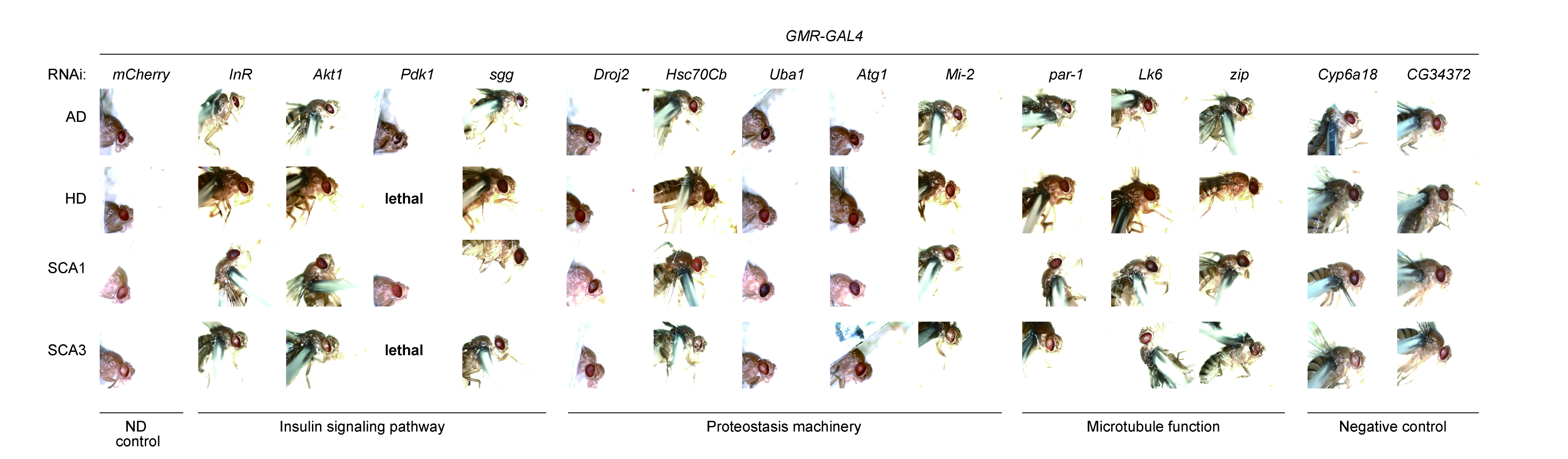

Supplement: Supplementary file 11 — Source Data for Figure 4 [file MSB-19-e11801-s012.zip › Figure 4/4A/4A.tif]
