# Supplementary material for: A multi‐layered network model identifies Akt1 as a common modulator of neurodegeneration
Source: Mol Syst Biol. 2023 Nov 20;19(12):e11801. doi: 10.15252/msb.202311801 (PMC10698508; doi:10.15252/msb.202311801)
Supplement: Supplementary file 13 [file MSB-19-e11801-s010.pdf]

molecular systems biology

Multi-layered network expansion model identifies Akt1 as common modulator of neurodegeneration

Joerg Gsponer, Dokyun Na, Do-Hwan Lim, Jae-Sang Hong, Hyang-Mi Lee, Daeahn Cho, Myung-Sang Yu, Bilal Shaker, Jun Ren, Bomi Lee, Jae Gwang Song, Yuna Oh, Kyung Eun Lee, Kwang-Seok Oh, Mi Young Lee, Min-Seok Choi, Han Saem Choi, Yang-Hee Kim, Jennifer Bui, Kangseok Lee, Hyung-Wook Kim, and Young Sik Lee
DOI: 10.15252/msb.202311801

Corresponding author(s): Joerg Gsponer (gsponer@msl.ubc.ca) , Young Sik Lee (ys-lee@korea.ac.kr)

Review Timeline:

Submission Date:	5th Jun 23
Editorial Decision:	13th Jul 23
Revision Received:	9th Oct 23
Editorial Decision:	23rd Oct 23
Revision Received:	25th Oct 23
Accepted:	27th Oct 23

Editor: Maria Polychronidou

Transaction Report:

(Note: With the exception of the correction of typographical or spelling errors that could be a source of ambiguity, letters and reports are not edited. Depending on transfer agreements, referee reports obtained elsewhere may or may not be included in this compilation. Referee reports are anonymous unless the Referee chooses to sign their reports.)

14th Jul 2023

Manuscript Number: MSB-2023-11801
Title: Multi-layered network expansion model identifies Akt1 as common modulator of neurodegeneration

Dear Dr Gsponer,

Thank you again for submitting your work to Molecular Systems Biology. We have now heard back from the three reviewers who agreed to evaluate your study. As you will see below the reviewers raise substantial concerns, which preclude the publication of the manuscript in its current form. Given that the reviewers did have positive words about the potential relevance of the approach, we have decided to offer you a chance to address the issues raised in a major revision.

Without repeating all the points listed below, some of the more fundamental issues are the following:

- The methodological advance and the performance of the method need to be better demonstrated.

- The biological conclusions need to be strengthened by including further experimental and computational analyses.

All issues raised by the reviewers need to be satisfactorily addressed. As you may already know, our editorial policy allows in principle a single round of major revision, so it is essential to provide responses to the reviewers' comments that are as complete as possible. I understand that the required revisions are substantive. Please feel free to contact me in case you would like to discuss in further detail any of the issues raised or if you would like to share your revision plan with me. I would be happy to schedule a call.

On a more editorial level, we would ask you to address the following points:

- Please provide a .doc version of the manuscript text (including legends for the main figures) and individual production quality figure files for the main Figures (one file per figure).

- The keywords need to be reduced to 5.

- Please provide a "standfirst text" summarizing the study in one or two sentences (approximately 250 characters), three to four "bullet points" highlighting the main findings and a "synopsis image" (550px width and max 400px height, jpeg format) to highlight the paper on our homepage.

- All Materials and Methods need to be described in the main text. We would ask you to use 'Structured Methods', our new Materials and Methods format, which is mandatory for Methods and Articles with a strong methodological focus. According to this format, the Materials and Methods section should include a Reagents and Tools Table (listing key reagents, experimental models, software and relevant equipment and including their sources and relevant identifiers) followed by a Methods and Protocols section in which we encourage the authors to describe their methods using a step-by-step protocol format with bullet points, to facilitate the adoption of the methodologies across labs. More information on how to adhere to this format as well as downloadable templates (.doc or .xls) for the Reagents and Tools Table can be found in our author guidelines: . An example of a Method paper with Structured Methods can be found here: .

- Please include a "Disclosure and Competing Interests Statement" in the main text.

- The code and the datasets should be made available (we do not allow data available upon request).

- Please include a Data availability section describing how the data, code etc. have been made available. This section needs to be formatted according to the example below:
The datasets and computer code produced in this study are available in the following databases:
- Chip-Seq data: Gene Expression Omnibus GSE46748 (https://www.ncbi.nlm.nih.gov/geo/query/acc.cgi?acc=GSE46748)
- Modeling computer scripts: GitHub (https://github.com/SysBioChalmers/GECKO/releases/tag/v1.0)
- [data type]: [full name of the resource] [accession number/identifier] ([doi or URL or identifiers.org/DATABASE:ACCESSION])

- For data quantification: please specify the name of the statistical test used to generate error bars and P values, the number (n) of independent experiments (specify technical or biological replicates) underlying each data point and the test used to calculate p-values in each figure legend. The figure legends should contain a basic description of n, P and the test applied. Graphs must include a description of the bars and the error bars (s.d., s.e.m.).

- Molecular Systems Biology supports formal data citations in the Reference list, to cite previously published datasets. In addition

to citing the original papers that reported the data, we encourage you to also cite the relevant datasets directly in the Reference list. In the text, references to datasets are included as "Data ref: Smith et al, 2001" or "Data ref: NCBI Sequence Read Archive PRJNA342805, 2017". In the Reference list, data citations are very similar to normal literature references but must be labeled with "[DATASET]" at the end of the reference. For detailed instructions please refer to our Author Guidelines .

- When you resubmit your manuscript, please download our CHECKLIST (https://bit.ly/EMBOPressAuthorChecklist) and include the completed form in your submission.
Please note that the Author Checklist will be published alongside the paper as part of the transparent process (https://www.embopress.org/page/journal/17444292/authorguide#transparentprocess).

If you feel you can satisfactorily deal with these points and those listed by the referees, you may wish to submit a revised version of your manuscript. Please attach a covering letter giving details of the way in which you have handled each of the points raised by the referees. A revised manuscript will be once again subject to review and you probably understand that we can give you no guarantee at this stage that the eventual outcome will be favorable.

Yours sincerely,

Maria Polychronidou, PhD
Senior Editor
Molecular Systems Biology

We realize that it is difficult to revise to a specific deadline. In the interest of protecting the conceptual advance provided by the work, we recommend a revision within 3 months (11th Oct 2023). Please discuss the revision progress ahead of this time with the editor if you require more time to complete the revisions. Use the link below to submit your revision:

https://msb.msubmit.net/cgi-bin/main.plex

IMPORTANT: When you send your revision, we will require the following items:
1. the manuscript text in LaTeX, RTF or MS Word format
2. a letter with a detailed description of the changes made in response to the referees. Please specify clearly the exact places in the text (pages and paragraphs) where each change has been made in response to each specific comment given
3. three to four 'bullet points' highlighting the main findings of your study
4. a short 'blurb' text summarizing in two sentences the study (max. 250 characters)
5. a 'thumbnail image' (550px width and max 400px height, Illustrator, PowerPoint or jpeg format), which can be used as 'visual title' for the synopsis section of your paper.
6. Please include an author contributions statement after the Acknowledgements section (see https://www.embopress.org/page/journal/17444292/authorguide)
7. Please complete the CHECKLIST available at (https://bit.ly/EMBOPressAuthorChecklist).
Please note that the Author Checklist will be published alongside the paper as part of the transparent process (https://www.embopress.org/page/journal/17444292/authorguide#transparentprocess).
8. When assembling figures, please refer to our figure preparation guideline in order to ensure proper formatting and readability in print as well as on screen:
https://bit.ly/EMBOPressFigurePreparationGuideline
See also figure legend guidelines: https://www.embopress.org/page/journal/17444292/authorguide#figureformat
9. Please note that corresponding authors are required to supply an ORCID ID for their name upon submission of a revised manuscript (EMBO Press signed a joint statement to encourage ORCID adoption).
(https://www.embopress.org/page/journal/17444292/authorguide#editorialprocess)
Currently, our records indicate that there is no ORCID associated with your account.

Please click the link below to provide an ORCID:
Link Not Available

10. At EMBO Press we ask authors to provide source data for the main manuscript figures. Our source data coordinator will contact you to discuss which figure panels we would need source data for and will also provide you with helpful tips on how to upload and organize the files.

The system will prompt you to fill in your funding and payment information. This will allow Wiley to send you a quote for the article processing charge (APC) in case of acceptance. This quote takes into account any reduction or fee waivers that you may be eligible for. Authors do not need to pay any fees before their manuscript is accepted and transferred to the publisher.

EMBO Press participates in many Publish and Read agreements that allow authors to publish Open Access with reduced/no publication charges. Check your eligibility: https://authorservices.wiley.com/author-resources/Journal-Authors/open-

access/affiliation-policies-payments/index.html

As a matter of course, please make sure that you have correctly followed the instructions for authors as given on the submission website.

*** PLEASE NOTE *** As part of the EMBO Press transparent editorial process initiative (see our Editorial at https://dx.doi.org/10.1038/msb.2010.72), Molecular Systems Biology publishes online a Review Process File with each accepted manuscripts. This file will be published in conjunction with your paper and will include the anonymous referee reports, your point-by-point response and all pertinent correspondence relating to the manuscript. If you do NOT want this File to be published, please inform the editorial office at msb@embo.org within 14 days upon receipt of the present letter.

Reviewer #1:

This is potentially an interesting paper where the authors have generated a new program, based on biological interaction data, to try to predict common modifiers of neurodegenerative disease. My background is in general biology, not bioinformatics, so my suggestions pertain to making the findings more translatable for general biologists.

Overall, it seems this could be a nice program. They demonstrate that it seems superior to other existing programs, and provide functional data of success. There is certainly a great need for this approach and using more global data to get experimental directions. It is also nice to see integration of model organism results to help predict pathways.

My suggestions have to do with clarity for a general biologist.

1- In many cases they discuss specific genes, but they do not include supplemental tables that list them. This would be most transparent and helpful, especially for others looking for interactions. For example, page 6 bottom they list the number of modifier genes they used for the different diseases, but don't include specifically what they are. They include reference to what they are, but it would be more helpful to include the specific list they used here. Similarly for top of page 7. Can provide a supplemental table of the top 100 seeds? I think more transparency will lead to great adoption of this approach. There are other examples of this, that they could include.

2- For the Drosophila studies, can they organize the genes by biology as they discuss them in the discussion top of page 15? This would make the data easier to see trends.

3- With these fly genetic interaction data, one wonders if one selected 4-6 random genes NOT on the list, would one also see interactions? If they could do such an experiment, it would be highly compelling for the power of their approach.

4- It is striking that the direction of effect in the fly is rather random given the disease. Also, they select an activator for the Akt pathway to use in mammalian cells (and then there it gave the same phenotype for everything, unlike in Drosophila). Can they include more rationale for choosing an activator rather than knocking the pathway down.

5- Were the fly, mouse and cell studies performed in blinded manner? They should have been and it would be more compelling if they were.

6- It might be nice to give an example where they following one gene all the way through their analysis and where it is and what its PPI are, etc. This may allow more clarity for basic biologists.

Reviewer #2:

Na & Lim et al. investigates the common gene modifiers across four different neurodegenerative diseases (NDs): Alzheimer's disease (AD), Huntington's disease, and Spinocerebellar ataxia types 1 and 3. The authors used a model they developed, the multi-layered network expansion (MLnet), to predict protein modifiers common to these diseases, identifying several members of the insulin pathway, including PDK1, Akt1, InR, and GSK-3β. Validation was carried out using Drosophila ND models and human cell-based ND models. They showed that the activation of Akt1 signaling by a small molecule, SC79, increased cell viability and improved memory and anxiety levels in AD model mice. Overall the study nicely combined computational, Drosophila, and in vivo evidence and was well-formulated/written.

General Remarks

The paper provides some compelling results and insights about the common molecular mechanisms across neurodegenerative

diseases, particularly pointing towards the insulin pathway. It appears to place the work in the broader context of investigating common proteinopathies, although the reviewer is not entirely convinced that the identified modifier mediates these diseases through proteinopathy-related pathways (could it be general cognition mediated through the insulin pathway?)

Overall, this study could be seen as a significant advance, specifically with the validation of the MLnet tool for computational biologists-the reviewer applauds the authors' efforts to create the webtool & think the authors should highlight it in the Abstract to increase impact. This research might also be of interest to neurobiologist albeit the reviewer does not have much expertise to judge from that front.

Major Points

• The work is predicated on the accuracy and validity of the MLnet model to generate good disease modifiers, which based on the authors assessment seem robust. But garbage-in-garbage-out, and the reviewers would like the authors to include lists of seed genes used. Also do you get better modifiers if you include just for example GWAS genes?

• There are implications that the insulin pathway could be a common pathway for these NDs, yet the mechanistic link between the insulin pathway and protein aggregation in these etiologies remains vague. Is it possible that the insulin pathway is linked to cognitive functions in general? This connection should be further explored and clarified both computationally and in experiments.

Minor Points

The effectiveness of SC79 is intriguing, particularly with in vitro and in vivo data. The reviewers caution that in cancer the PI3K-akt signaling is often activated and the authors will likely want to monitor the possible tumorigenic effects. Even if the compound can be made brain-specific, glioblastoma could be a concern as well.

Reviewer #3:

MSB-2023-11801 "Multi-layered network expansion model identifies Akt1 as common modulator of neurodegeneration" by Na et al. In this m/s, the authors present MLnet, a new methodology (based on a multi-layered network expansion strategy) to identify protein modifiers common to several neurodegenerative diseases. In particular, the authors apply it to Alzheimer´s (AD) and Huntington (HD) diseases as well as spinocerebellar ataxias type 1 and 3 (SC1, 3). The authors tested the 12 top-scoring proteins on fly disease models for the AD, HD, SC1 and SC3, finding that 4 of them showed a phenotypic effect. They also found that these genes were related to the insulin pathway, and selected Akt1 (as a central gene in this pathway) for further validation on HEK293 cells and the 5xFAD mouse model. The m/s covers an interesting area of research, and it is well-structured and clearly written. However, there are a few conceptual and methodological aspects that need to be clarified and/or corrected. Without these clarifications, it is difficult a assess the added value of the MLnet method and the novelty of the results presented in the paper. In its present forms, I fail to see the rationale behind the experiment and the advance (methodologic and/or scientific) provided by the m/s. Please, find below some specific comments that might help the authors to improve the quality and clarity of their m/s.

I understand the appeal of finding a common mechanism (modulator) for all neurodegenerative diseases, but I fail to see any support for this hypothesis. May be the authors could elaborate a bit more on the evidence leading to this research. I also fail to see the rationale, or advantage, in identifying a common drug target. Do these diseases manifest together? And, in case that there are common mechanisms/modulators, are these expected to be unique for neurodegenerative diseases, or are also shared in other complex diseases?

To report the MLnet performance results, the authors always refer to the area under the curve (AUC), but they do not specify to which curve they refer. Given the high values reported, I assume it is the AUROC curve, but they should clarify it. If this is correct, ROC curves are seriously affected by data imbalance (i.e. if the number of positives is small, you can get high AUROC values predicting that everything is a negative). The authors should clearly describe the ratio between modulators and non-modulators in every set, and also report individual values of precision and recall (or the AUPRC).

It is also not clear how the MLnet results compare to just using the common gene set identified for each individual ND. I understand that the network propagation might find more common genes, but it is unclear whether these would indeed be relevant (i.e. real modulators) for each disease. The authors should also compare if the MLnet identified common ND modulators have been reported to be associated to the individual diseases. Without this, it is difficult to assess the added value of the methodology.

The common ND modifiers identified by MLnet are enriched in apoptosis, autophagy and mitophagy. How about the seed modifiers used for each disease? If they are not, this is likely to be an artifact of just identifying very well connected general biological processes. To avoid this potential bias, the authors should also use randomized networks, preserving the connection degree of the proteins and shuffling the edges. Calculating enrichments using only the number of genes in a process identified

might not be enough. It would also be interesting to see if these general processes are somehow specific for NDs, or they are found in other unrelated complex diseases which, again, could just be the result of their centrality.

4 out of the 12 potential modulator proteins tested in flies gave a signal. However, it is unclear what is the added value of MLnet in identifying common modulators. How close to the top are these 4 (or 12) proteins in the individual disease models? What would be the result if the authors would pick the top proteins in each individual ND and test them in all four fly models? Is the enrichment in the insulin pathway detected in the individual diseases (see point above)? What about other genes in the pathway not selected as common modifiers, would they show an effect?

I do not really understand the experiment in HEK293 cells. Why are cells expressing ND-related genes expected to be less viable? Specific ND-related genes do not affect cell viability (and certainly not in HEK293 cells). The authors should clarify this experiment and the interpretation of the results.

In the last section, the authors kind of drop all the previous results and only focus on testing the effect of the insulin pathway in Alzheimer´s disease which, as they state, has been clearly established! I fail to see the added value of re-reporting that the modulation of the insulin pathway has an effect on the 5xFAD mouse model. If anything, they should test the effect of this modulation of models other that AD (i.e. HD or the SCs). The authors also say that GSK3b is the only identified AD-HD modifier not directly involved in the insulin pathway, but there is an infinity of literature relating GSK3b and insulin levels. Moreover, GSK3b inhibitors have been extensively tested to treat AD, showing very promising results in mice models, but failing the clinical trials.

Finally, the number of animals used in the Barnes maze test (4/5 mice) seems insufficient. The learning curve for the AD model is not really consistent in the days 3,4 and 5. The same effect is observed in the test days (15 and 16). Although, Fig 5G shows a mild significance when comparing days 5 and 16 between AD and AD+SC79, this difference should be supported by a robust learning curve, or any small change (i.e. adding one more animal or changing the days compared) could wipe it out.

Point-by-point responses

First of all, we would like to thank the reviewers for their encouraging comments and valuable suggestions. We believe that, by addressing the reviewers' concerns, a considerably stronger manuscript has emerged. Please find our point-by-point responses below.

Responses to Reviewer #1

1- In many cases they discuss specific genes, but they do not include supplemental tables that list them. This would be most transparent and helpful, especially for others looking for interactions. For example, page 6 bottom they list the number of modifier genes they used for the different diseases, but don't include specifically what they are. They include reference to what they are, but it would be more helpful to include the specific list they used here. Similarly for top of page 7. Can provide a supplemental table of the top 100 seeds? I think more transparency will lead to great adoption of this approach. There are other examples of this, that they could include.

We appreciate this valuable suggestion. We have now added three supplementary tables (**Tables S1**, **S2 and S3**) to the manuscript. **Table S1** includes the genetic modifiers that we obtained from the NeuroGeM database and used to predict disease-specific modifiers in the first module. **Table S2** includes the top 100 predicted disease-specific modifiers, i.e., seed genes used for the second module of MLnet. **Table S3** contains the top100 common modifiers predicted by MLnet.

2- For the Drosophila studies, can they organize the genes by biology as they discuss them in the discussion top of page 15? This would make the data easier to see trends.

We thank the reviewer for this great suggestion. We have rearranged the eye phenotype images in the revised **Fig 4**, grouping them according to their biological function.

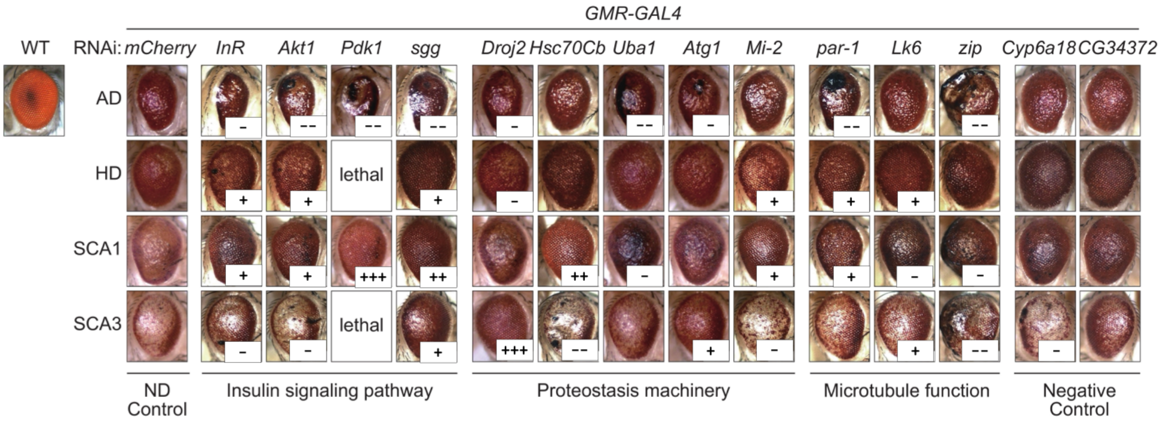

Figure 4. Changes in the rough eye phenotype of *Drosophila* models for AD, HD, SCA1, and SCA3 due to knockdown of predicted common modifiers. Representative bright-field microscope images of fly eyes with *GMR-GAL4*-driven misexpression of the ND-causing genes, along with *GMR-GAL4*-driven RNAi against each of the indicated genes in which *mCherry* served as a control. Compared with the wild-type (WT) compound eye with the ordered structure of ommatidia, flies with misexpression of individual ND-causing genes and RNAi against *mCherry* under the control of the *GMR-GAL4* driver had rough eyes with the variation in phenotypic severity. Suppression or enhancement of these rough eye phenotypes caused by RNAi-mediated knockdown of the predicted common modifiers is indicated with + or -, respectively. As a negative control, two genes (Cyp6a18 and CG34372) randomly selected among low-ranked genes were also tested.

3- With these fly genetic interaction data, one wonders if one selected 4-6 random genes NOT on the list, would one also see interactions? If they could do such an experiment, it would be highly compelling for the power of their approach.

This is an excellent suggestion. We agree that evaluating low-ranked genes as negative controls can provide further support for the power of our approach. Following this reviewer's suggestion, we selected two genes randomly from the bottom end of the ranking list (MLnet scores) and assessed their impact on the eye phenotype in the different ND models. As expected, these low-ranked genes did exhibit minimal to no modifier capability when altered in expression. We have included these results in **Fig 4** and the ***Results*** section of the revised manuscript.

(Page 14)

Four of the 12 tested common modifier proteins (Akt1, InR, Pdk1, and sgg (GSK3β)) changed the eye phenotypes in all four ND models when down-regulated by RNAi. Interestingly, three of these (*Akt1*, *InR*, and *Pdk1*) are directly involved in insulin signaling and one of them (*sgg*) acts downstream of the insulin signaling pathway (**Fig 3C**). As a negative control, we also evaluated the effect of two randomly selected low-ranked genes (*Cyp6a18* and *CG34372*) and found them to have little to no impact across the four *Drosophila* ND models (**Fig 4**). To validate the top 12 ranked proteins further, we searched the literature for evidence that supports the impact of them in specific NDs (**Table S5)** and, indeed, we could find evidence across diseases for many of these proteins.

4- It is striking that the direction of effect in the fly is rather random given the disease. Also, they select an activator for the Akt pathway to use in mammalian cells (and then there it gave the same phenotype for everything, unlike in Drosophila). Can they include more rationale for choosing an activator rather than knocking the pathway down.

The varying effects of modifiers on the eye phenotype is, indeed, an interesting finding. However, the *Drosophila* eye phenotype is determined by complex cellular processes, which are regulated differently in various contexts (diseases) due to distinct gene expression profiles and interactions. As we discuss in the manuscript (**page 18**), there exist multiple previous studies in which opposing effects of the same gene were reported. Effects on a specific phenotype (e.g., eye morphology, cell viability, etc.) can vary depending on the method and level of gene expression modulation and the disease under investigation. In our previous study in which we assembled a database of genetic modifiers of neurodegenerative diseases and analyzed the collected data, we found many instances in which opposite effects had been reported for the same gene (Na et al, 2013). Importantly, the consensus among experts who use genetic screens (particularly in *D. melanogaster*) is that it is hard to decide based on the screen results whether a gene is an enhancer or suppressor. Therefore, the results of the *D. melanogaster* experiments (**Fig 4**) should be interpreted as evidence for the ability of the positively-tested genes to act as disease modifiers rather than enhancers or suppressors.

Regarding Akt1, there is increasing evidence that increased activity of insulin signaling can enhance the protection of neuronal cells. Since decreased activity of or resistance in insulin signaling is often found in the patients of AD, there has been clinical attempts/trials to treat AD with nasal administration of insulin. Therefore, we hypothesized that activation of the insulin signaling pathway could alleviate neurodegenerative phenotypes. As there is an activator for Akt1, we chose Akt1 as a target to modulate. We updated the main text to reflect our motivation in choosing an Akt1 activator.

Reference:
Na, D., Rouf, M., O'Kane, C. J., Rubinsztein, D. C. & Gsponer, J. NeuroGeM, a knowledgebase of genetic modifiers in neurodegenerative diseases. *BMC Med. Genomics* **6**, 52 (2013).

(Page 18)
Consistent with these previous studies, we find that all 12 top-ranked proteins, when suppressed in expression, modulate disease phenotype in at least two of the tested models. However, only proteins that are part of the insulin pathway affect phenotypes in all of the tested *D. melanogaster* disease models. Interestingly, supressing the expression of these modifiers enhances the phenotype in some ND models, while it reduces it in others. These observations are consistent with previous studies where overexpression of the same gene can have opposing effects on the phenotypic of different ND when tested in fly models (Branco *et al*, 2008). These differences are explained by the fact that the impact of genetic modulation on ND phenotypes is highly dependent on the method and level of modulation and the complexity of the pathophysiology of the individual ND (Na *et al*, 2013). Therefore, the results of the *D. melanogaster* experiments (**Fig 4)** should be interpreted as evidence for the ability of the positively-tested genes to act as disease modifiers rather than enhancers or suppressors.

(Page 15)
Motivated by these findings, we aimed to test the disease-modifying impact of insulin signaling in mammalian models of ND. Since decreased activity of or resistance in insulin signaling is commonly found in the patients of AD, we hypothesized that activation of the insulin signaling pathway could alleviate neurodegenerative phenotypes. We chose *Akt1* as a target for insulin signaling modulation because of its central position in this

pathway. Akt1 is not ranked very high in the disease-specific modifier lists, with the exception of SCA1 (**Table S5**), but is second in the final ranking of common modifiers due to its interaction with many proteins that are themselves disease modifiers, i.e. partners that are highly ranked in the disease-specific modifier lists of module 1 (see **Appendix text** for details). Moreover, the availability of an activator of this kinase enables induction of downstream insulin signaling (Jo *et al*, 2012).

5- Were the fly, mouse and cell studies performed in blinded manner? They should have been and it would be more compelling if they were.

The *Drosophila* experiments were performed in a blinded manner. Following the acquisition of eye images, the levels of rough eyes were scored without knowledge of the modifiers' names. For cell and mice experiments, all *in vitro* assays and behavioral tests were also conducted in a blinded manner. In addition, the mouse behavior was analyzed with the ANY-maze software, a behavioral test analysis program that aims to reduce the risk of bias. This is now mentioned in *Materials and Methods*. We thank the reviewer for this comment.

(Page 28)
The fly eyes were photographed under the same adjustment setting of I-MEASURE software for capturing images. The *Drosophila* experiments were performed in a blinded manner.

(Page 29)
The optical density (OD) of each well was measured using a microplate reader at 450 nm (Molecular Devices, CA, USA), and the OD values were reported as % cell viability (mean ± standard error, $n = 4 - 8$ per group). The *in vitro* assays were performed in a blinded manner.

(Page 30)
All trials were recorded and analyzed by ANY-maze 6.0 Software. All behavioral tests were conducted in a blinded manner and the ANY-maze software was used to avoid any bias in the analysis.

6- It might be nice to give an example where they following one gene all the way through their analysis and where it is and what its PPI are, etc. This may allow more clarity for basic biologists.

We thank the reviewer for this great suggestion. We now provide stepwise “evidence” for how Akt1 acquires a top rank compared to two other proteins in the **Appendix Text S1**. Specifically, we provide Akt1’s data on experimental testing, its rank as disease-specific modifier and information on how its interaction partners are ranked in individual diseases and thereby contribute to its high overall rank as common modifier:

1. Modification of Akt1 in NDs

Akt1 has been tested in models of HD and SCA1, but not of AD and SCA3 (**Table S7**, below). Akt1's assigned confidence score for the experimental results in HD and SCA1 models are 0.60 and 0.72, respectively. The high confidence scores compared with other modifiers (half of HD modifiers have a confidence score lower than 0.2) are due to the fact that Akt1 was identified as a modifier in several low-throughput experiments.

Table S7. Akt1 as a modifier and its confidence score in NDs

	**AD**	**HD**	**SCA1**	**SCA3**
**Known modifier?**	No	Yes	Yes	No
**Confidence score**	0	0.60	0.72	0

2. Ranks of Akt1 in the predicted disease-specific modifiers

Among disease-specific modifiers predicted by the first module of MLnet, Akt1 ranks only within the top 100 in SCA1 (**Table S8**, see below). However, Akt1 ranks second among common modifiers, which shows that the multi-layered networks approach of the second module of MLnet significantly changes the ranks. For comparison, two additional Drosophila genes are listed that are ranked very low in the individual diseases (not tested experimentally), don't get a high common modifier ranking and have a similar number of protein interaction partners as Akt1 in the network.

Table S8. Ranks of Akt1, CG7470, and CG10749 within predicted disease-specific modifiers

	**AD**	**HD**	**SCA1**	**SCA3**
**Akt1**	113	383	15	260
**CG7470**	10,504	13,498	10,186	9,602
**CG10749**	8,344	6,762	11,144	6,600

3. *Ranks of Akt1's interaction partners in the predicted disease-specific modifiers*.

In MLnet, the common modifier score (and, thus, the rank) of a protein generated in module 2 depends on its own seed score (from module 1) as well as the seed scores (from module 1) and the updated common modifier score of its interaction partners in the network (see **Fig S2**). As Akt1 acquires a high common modifier score but is initially not top ranked in individual diseases, it can be assumed that it gets this high final score because the scores of Akt1's interaction partners are high across the different diseases, higher than those of proteins that don't get pushed to the top of the common modifier list by module 2. Therefore, we investigated the distribution of the ranks of interacting partners of Akt1 and two *Drosophila* genes (CG7470 and CG10749) that are not ranked high among predicted common modifiers (they are ranked 1102 and 4827, respectively). **Fig S14** shows the histogram of the ranks of interaction partners of these proteins in the individual diseases. As only top 100 proteins from each disease get a seed score, only the number of interaction partners that are part of these 100 seeds are shown. As can be seen from **Fig S14**, Akt1 has, consistently across all diseases, more interaction partners that are top ranked, i.e., that are part of the 100 seed proteins.

Consequently, Akt1's rank in the list of predicted common modifiers is high in contrast to two other genes that have a similar number of interaction partners as Akt1 but much fewer interaction partners that are top ranked in individual disease and used as seeds.

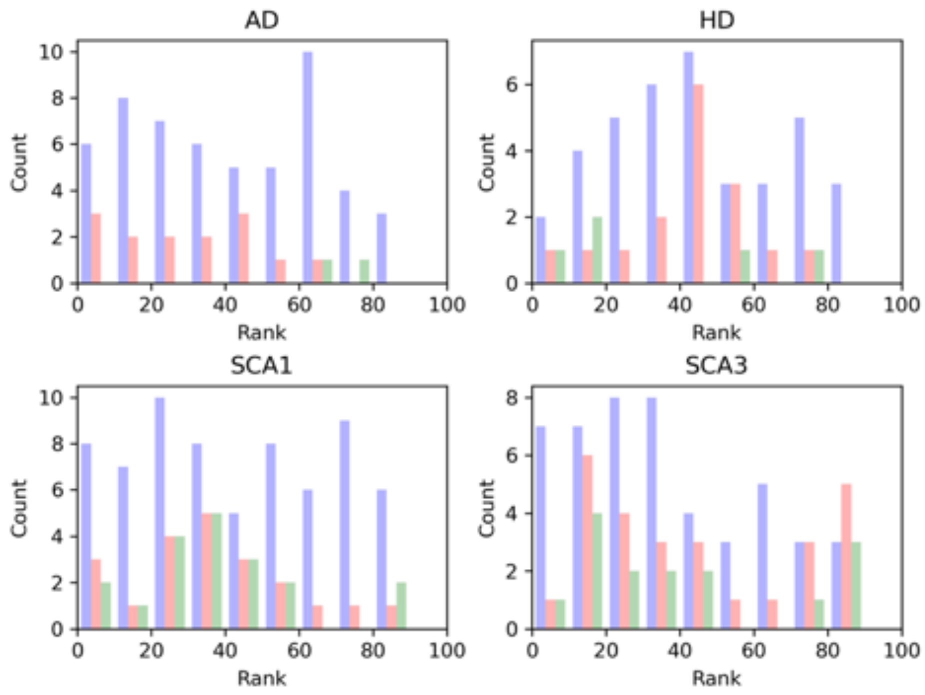

Appendix Figure S14. Histogram of interaction partner ranks.
Interaction partners of Akt1 (blue), CG7470 (red), and CG10749 (green) in the predicted disease-specific modifier lists are binned according to their ranks and the count per bin is provided. Ten bins are used for the top 100 ranks of each ND.

Please **Appendix Text S1** for the updated information.

Responses to Reviewer #2:

Overall, this study could be seen as a significant advance, specifically with the validation of the MLnet tool for computational biologists-the reviewer applauds the authors' efforts to create the webtool & think the authors should highlight it in the Abstract to increase impact. This research might also be of interest to neurobiologist albeit the reviewer does not have much expertise to judge from that front.

We appreciate the reviewer's encouraging comment. As suggested, we revised the *Abstract* to emphasize the availability of the new web-tool.

Abstract

The accumulation of misfolded and aggregated proteins is a hallmark of neurodegenerative proteinopathies. Although multiple genetic loci have been associated with specific neurodegenerative diseases (NDs), molecular mechanisms that may have a broader relevance for most or all proteinopathies remain poorly resolved. In this study, we developed a multi-layered network expansion (MLnet) model to predict protein modifiers that are common to a group of diseases and, therefore, may have broader pathophysiological relevance for that group. When applied to the four NDs, Alzheimer's disease (AD), Huntington's disease, and Spinocerebellar ataxia types 1 and 3, we predicted multiple members of the insulin pathway, including PDK1, Akt1, InR, and sgg (GSK-3β), as common modifiers. We validated these modifiers with the help of four *Drosophila* ND models. Further evaluation of Akt1 in human cell-based ND models revealed that activation of Akt1 signaling by the small molecule SC79 increased cell viability in all models. Moreover, treatment of AD model mice with SC79 enhanced their long-term memory and ameliorated dysregulated anxiety levels, which are common symptoms of AD patients. These findings validate MLnet as a valuable tool to uncover molecular pathways and proteins involved in the pathophysiology of entire disease groups and identify potential therapeutic targets that have relevance cross disease boundaries. MLnet can be used for any group of diseases, and is available as a web tool at http://ssbio.cau.ac.kr/software/mlnet.

Major Points

• The work is predicated on the accuracy and validity of the MLnet model to generate good disease modifiers, which based on the authors assessment seem robust. But garbage-in-garbage-out, and the reviewers would like the authors to include lists of seed genes used.

We agree with the reviewer's concern about the reliability of the input data. We collected the known genetic modifiers from the NeuroGeM database that we have built previously, which contained a manually curated list of experimentally confirmed genetic modifiers of *Drosophila*. Importantly, we developed a confidence score that is based on the type and number of experiments showing that a specific gene is a genetic modifier of a specific neurodegenerative disease. We only used high-confidence modifiers as input to MLnet. We added three supplementary tables (**Tables S1**, **S2 and S3**) to the manuscript. **Table S1** provides the list of known, high-confidence genetic modifiers of the four NDs obtained from the NeuroGeM and used as initial input to MLnet. **Table S2** provides the 100 top-ranked predicted disease-specific modifiers from module 1 of MLnet that were used as seed genes

for the prediction of common genetic modifiers in module 2. **Table S3** contains the top100 common modifiers predicted by MLnet.

• Also do you get better modifiers if you include just for example GWAS genes?

This is a great suggestion. We actually used GWAS genes when testing MLnet on human data. We have predicted human modifier genes common to AD and HD using the genes deposited in Neurocarta. Neurocarta compiles not only genes identified using animal and cell-based models but also GWAS genes associated with neurological disorders. As shown in **Fig S11**, the PI3K-Akt signaling (i.e., the insulin signaling) is most highly enriched within the predicted human AD/HD common genes. Notably, GWAS genes should be carefully included because of the high number of false positives.

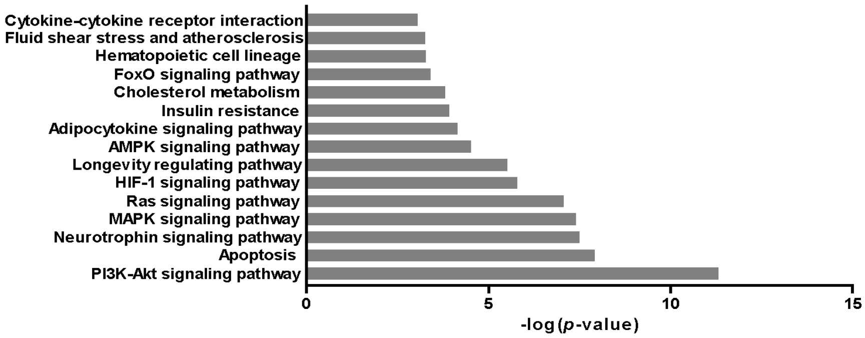

Appendix Figure S11. Pathway enrichment analysis result for predicted common modifiers of human AD and HD.

• There are implications that the insulin pathway could be a common pathway for these NDs, yet the mechanistic link between the insulin pathway and protein aggregation in these etiologies remains vague. Is it possible that the insulin pathway is linked to cognitive functions in general? This connection should be further explored and clarified both computationally and in experiments.

We thank the reviewer for this very important comment. Indeed, insulin plays a key role in brain plasticity and neurogenesis, both impacting cognitive functions such as learning and memory (Spinelli et al, 2019). In the hippocampus, for instance, insulin positively impacts synaptic and structural plasticity. Some of these effects of insulin are mediated by members of the insulin signaling pathway like PI3-kinase and Akt1 or downstream effectors such as GSK3β and mTORC1. For instance, PI3-kinase is essential for glutamate receptor insertion at plasma membranes during synaptic plasticity (Man et al, 2003). We additionally tested the *Drosophila* homolog of PI3-kinase, Pi3K92E, for its ability to modulate the

neurodegenerative phenotype in the four *Drosophila* models. The newly added results confirm that it is a common modifier.

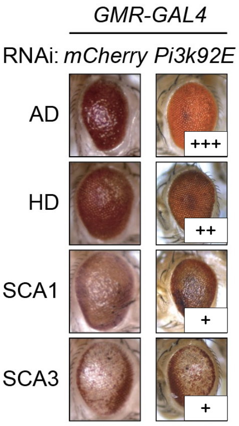

Appendix Figure S13. Effect of Pi3K92E knockdown on *Drosophila* eye phenotype in four ND models.

We now discuss the involvement of insulin in brain plasticity and cognition in much more detail. We highlight our finding that amyloid-β in the brain of AD mice treated with SC79 is not decreased compared to mice that were not treated, which may suggest that, besides reduced apoptosis, insulin pathway-mediated effects on brain plasticity contribute to the improved cognitive test results seen for the SC79-treated mice. To provide further support to this idea, we also analysed genes that have recently been associated with human adult cognitive function (Chen et al, 2023). Of the eight genes that have been associated with human adult cognitive function through rare coding variants with large effects, four had previously been shown to affect insulin and the insulin pathway, although this links has been established with peripheral and not cerebral insulin (Backe et al, 2019; Giovannone et al, 2003; Hamming et al, 2010; Gonzalez et al, 2022).

Concluding, we now discuss the possibility of changes in cognition in more detail and provide additional data in support of this idea. However, as much as we are curious to know more about this possible explanation for the importance of the insulin pathway, as is this reviewer, we feel that more experiments in that direction are outside the scope of this study, because the main focus is on introducing MLnet and not on revealing the intricate molecular details of the interplay between insulin signaling, brain plasticity and cognitive decline in neurodegeneration.

References:

Backe MB, Jin C, Andreone L, Sankar A, Agger K, Helin K, Madsen AN, Poulsen SS, Bysani M, Bacos K, et al (2019) The lysine demethylase KDM5B regulates islet function and glucose homeostasis. Journal of Diabetes Research 2019: e5451038

Chen C-Y, Tian R, Ge T, Lam M, Sanchez-Andrade G, Singh T, Urpa L, Liu JZ, Sanderson M, Rowley C, et al (2023) The impact of rare protein coding genetic variation on adult cognitive function. Nat Genet 55: 927–938

Giovannone B, Lee E, Laviola L, Giorgino F, Cleveland KA & Smith RJ (2003) Two novel proteins that are linked to insulin-like growth factor (IGF-I) receptors by the Grb10 adapter and modulate IGF-I signaling. Journal of Biological Chemistry 278: 31564–31573

González BJ, Zhao H, Niu J, Williams DJ, Lee J, Goulbourne CN, Xing Y, Wang Y, Oberholzer J, Blumenkrantz MH, et al (2022) Reduced calcium levels and accumulation of abnormal insulin granules in stem cell models of HNF1A deficiency. Commun Biol 5: 779

Hamming KSC, Soliman D, Webster NJ, Searle GJ, Matemisz LC, Liknes DA, Dai X-Q, Pulinilkunnil T, Riedel MJ, Dyck JRB, et al (2010) Inhibition of β-cell sodium-calcium exchange enhances glucose-dependent elevations in cytoplasmic calcium and insulin secretion. Diabetes 59: 1686–1693

Man H-Y, Wang Q, Lu W-Y, Ju W, Ahmadian G, Liu L, D'Souza S, Wong TP, Taghibiglou C, Lu J, et al (2003) Activation of PI3-kinase is required for AMPA receptor insertion during LTP of mEPSCs in cultured hippocampal neurons. Neuron 38: 611–624

Spinelli M, Fusco S & Grassi C (2019) Brain insulin resistance and hippocampal plasticity: Mechanisms and biomarkers of cognitive decline. Front Neurosci 13: 788

We updated the text accordingly:

(Page 15)

Given the positive testing of all four genes related to the insulin pathway, we decided to assess the impact of another insulin pathway protein on the disease phenotypes. We evaluated Pi3K92E (PI3K in **Fig 3C**) because it is within the top 20 of the predicted common modifiers. Moreover, PI3K is of particular interest because it is one of the key mediators of the insulin pathway's impact on brain plasticity and neurogenesis. For instance, PI3-kinase is essential for glutamate receptor insertion at plasma membranes during synaptic plasticity (Man *et al*, 2003). Downregulation of Pi3k92E changed the eye phenotype in all four ND models (**Fig S13**), confirming the significance of insulin signaling for the model phenotypes investigated here.

(Page 19)

Specifically, insulin regulates glucose homeostasis and maintains energy requirements for different neuronal functions. It is vital for neuronal growth and differentiation as well as neuroprotection by modulating autophagy, mitochondrial function, ER stress, and apoptosis (Pomytkin *et al*, 2018; Burillo *et al*, 2021). Thus, dysfunction of insulin signaling makes neuronal cells vulnerable to metabolic and cellular stresses (Kim & Feldman, 2015). Moreover, the insulin signaling pathway plays key roles in brain plasticity, impacting cognitive functions such as learning and memory (Spinelli et al, 2019). In the hippocampus, for instance, insulin positively impacts synaptic and structural plasticity. Recently, eight genes have been associated with human adult cognitive function through rare coding variants with large effects. Four of these eight genes had previously been shown to affect insulin and the insulin pathway, although this links has been established with peripheral and not cerebral insulin (Chen et al, 2023; Backe et al, 2019; Giovannone et al, 2003; Hamming et al, 2010; González et al, 2022).

(Page 20)

When we investigated the levels of amyloid-β in the brain of AD mice treated with or without SC79, there were no significant changes in amyloid-β levels whether insulin signaling was activated or not (**Fig S15**). As activated insulin signaling improved cell viability in *in-vitro* assays (**Fig 5B-E**), this may suggest that activation of Akt1 in our experiments may have enhanced anti-apoptotic effects while impacting autophagy to a lesser extent. Insulin actually experts anti-apoptotic effects *via* Akt1, which reduces the mitochondrial release of cytochrome c (Li *et al*, 2009; Kang *et al*, 2003). Alternatively, activation of Akt1 may be beneficial *via* its regulatory impact on cognitive functions (Spinelli *et al*, 2019).

Minor Points

The effectiveness of SC79 is intriguing, particularly with in vitro and in vivo data. The reviewers caution that in cancer the PI3K-akt signaling is often activated and the authors will likely want to monitor the possible tumorigenic effects. Even if the compound can be made brain-specific, glioblastoma could be a concern as well.

We fully agree with the reviewer. We already mentioned this caveat in the original manuscript. There are actually Akt1 inhibitors in clinical trials for the treatment of various cancers. Intriguingly though, there exists an inverse correlation between AD and cancer incidence (Ospina-Romero et al, 2020). AD patients are less prone to cancer. Notably, this inverse relationship between AD and cancer incidence becomes more pronounced with age. As cerebral insulin and PI3K-Akt activities have commonly been found reduced in AD patients, one may speculate that there exists a therapeutic window for controlled activation, if a brain-specific Akt1 activator could be developed.

Ospina-Romero M, Glymour MM, Hayes-Larson E, Mayeda ER, Graff RE, Brenowitz WD, Ackley SF, Witte JS & Kobayashi LC (2020) Association between Alzheimer disease and cancer with evaluation of study biases: A systematic review and meta-analysis. *JAMA Netw Open* 3: e2025515

In any case, we now make the potential danger clearer:

(Page 20)
Moreover, although our experiments suggest a modulatory role of Akt1 for multiple NDs, it may not be an ideal target for ND treatment development because of its involvement in numerous cellular processes and the fact that its enhanced activation can lead to cancerous cell transformation (Wang *et al*, 2017), which would require very close monitoring for tumorigenic effects when activated *via* a therapeutic agent.

Responses to Reviewer #3

1. I understand the appeal of finding a common mechanism (modulator) for all neurodegenerative diseases, but I fail to see any support for this hypothesis. May be the authors could elaborate a bit more on the evidence leading to this research. I also fail to see the rationale, or advantage, in identifying a common drug target. Do these diseases manifest together?

We are sorry that we did not make this point clear enough in the initial submission. Intracellular protein misfolding and aggregation are features that are *common* to essentially all late-onset neurodegenerative diseases, called proteinopathies. These include Alzheimer's disease, Parkinson's disease, tauopathies, forms of motor neuron disease (e.g., TDP-43 mutations), and the nine polyglutamine expansion diseases exemplified by Huntington's disease (HD), spinocerebellar ataxia type 1 (SCA1), and SCA3. Protein homeostasis (proteostasis) is crucial to the prevention of protein aggregation and has been demonstrated to decline with age and in proteinopathies. In this context, it is important to note that only a minority of proteinopathies occur as familial forms, i.e., they are caused by disease-causing mutations of high penetrance that are familial and can be directly linked to the proteins that misfold and aggregate. The majority of these diseases are idiopathic in nature. Even for the case of familial proteinopathies, genetic variation has been shown to affect the phenotype. Indeed, only between 40 and 70 % of the variance in the age of onset of HD and SCA can be accounted for by CAG repeat number in the disease-causing allele. Thus, significant research has been carried out to identify genes that increase the likelihood of proteinopathies, lower the age of onset and enhance the disease severity (called modifiers), predominantly via genetic screen in model organisms. Maybe not surprisingly, functional enrichment analyses of identified modifiers revealed that many of them are associate with proteostasis, i.e., defects in proteostasis may enhance generally the likelihood for protein aggregation. Importantly, modifiers of one proteinopathy can influence another - e.g., a significant fraction of SCA3 modifiers in Drosophila had similar effects in tau (Alzheimer) models (please check Figure 2). Therefore, we hypothesised that there is a subset of genetic modifiers that affects the severity of one of these diseases that has broader relevance and may modify several other or even all proteinopathies. One may expect that at least some of these *common* modifiers have functions associated with proteostasis, given the importance of proteostasis in preventing aggregation.

We believe that identifying such an "Achilles heel" is not only relevant for a better understanding of the pathophysiology of proteinopathies but may also be useful from a disease monitoring and therapeutic points of view. Monitoring activity of common modifiers may serve as biomarkers of proteostatic or other relevant cellular activity and as indicators for disease risk across multiple proteinopathies. Moreover, altering the activity of the common modifiers directly or indirectly may slow disease progression or delay the age of disease onset independent of the type of proteinopathy.

We revised our manuscript to clarify this important point:

(Page 5)

NDs belong to the ever-growing group of diseases called proteinopathies (Hipp *et al*, 2014), because intracellular protein misfolding and aggregation are common to these diseases. Protein homeostasis (proteostasis) is crucial to the prevention of protein aggregation and has been demonstrated to decline with age

and in proteinopathies. (Labbadia & Morimoto, 2015; Balch *et al*, 2008; Hipp *et al*, 2019). Given the fact that protein misfolding and aggregation is common to proteinopathies and modifiers of one proteinopathy can influence another, e.g., a significant fraction of SCA3 modifiers in Drosophila had similar effects in Alzheimer models, we hypothesized that there may exist a subset of genetic modifiers that has broader relevance and may modify several or even all proteinopathies. Such common or generic modifiers may be central hubs in proteostatic control or key regulators of the cellular stress response. A bioinformatics analysis that we carried out on existing modifier sets previously revealed, however, only a small and incoherent set of modifiers that were identified in multiple ND models (Na *et al*, 2013), which may be due to the limited power and coverage of high-throughput screens for modifiers. Therefore, we set out to develop a robust computational framework that, with the help of data integration, predicts protein modifiers common to multiple diseases. We believe that identifying modifiers is not only relevant for a better understanding of the pathophysiology of proteinopathies but may also be useful from a disease monitoring and therapeutic points of view. Common modifiers may serve as biomarkers, and monitoring their activity indicate disease risk across multiple proteinopathies. Moreover, altering the activity of the common modifiers directly or indirectly may slow disease progression or delay the age of disease onset independent of the type of proteinopathy.

There are several reviewer comments that relate to the same or related issues, so we merged them.

2-1. And, in case that there are common mechanisms/modulators, are these expected to be unique for neurodegenerative diseases, or are also shared in other complex diseases?

2-2. Calculating enrichments using only the number of genes in a process identified might not be enough. It would also be interesting to see if these general processes are somehow specific for NDs, or they are found in other unrelated complex diseases which, again, could just be the result of their centrality.

2-3. Is the enrichment in the insulin pathway detected in the individual diseases (see point above)?

We appreciate these important comments. Our MLnet does not show any significant bias toward network hub proteins, which are known to play roles across multiple diseases (please refer to our response to **Point 5**). Nevertheless, to investigate this point further and follow the reviewer's suggestion, we investigated whether the insulin signaling pathway would automatically come up as common to another disease group because of its centrality in cellular metabolism. To the end, we collected disease-related genes of three inflammatory diseases (gastroenteritis, hepatitis, and dermatitis). We hypothesized that inflammatory diseases have a set of commonly associated genes that are distinct from the common modifiers of proteinopathies and, more importantly, that these genes have different functions. Consistent with this hypothesis, we find inflammation- and immune response-related pathways enriched among the predicted common modulators of inflammatory diseases and no enrichment for the insulin or related pathways.

Regarding the enrichment of insulin pathway genes as modifiers of individual disease, such an enrichment may not generally be found for all NDs. It should be so if the genetic screens carried out for different disease cover the same set of genes. However, this is generally not the case. If only few insulin pathway-related genes were tested in screens of a specific disease, it is less likely that a GO enrichment analysis will show the insulin pathway as significant. This difference in data coverage is one of the reasons why we developed MLnet. Integrating the data across disease should help reduce those imbalances and help reweight genes that were not or only rarely tested in some diseases.

(Page 13-14)

As the insulin signaling pathway plays a central role in metabolism and the proteins that are part of it interact with many partners, we investigated whether this pathway would automatically come up in our network-based approach even when using genes associated with diseases not related to neurodegeneration. To this end, we collected disease-related genes of three inflammatory diseases (gastroenteritis, hepatitis, and dermatitis) and tested for annotations enriched among the top-ranked proteins predicted by MLnet to be common to these diseases. Specifically, we collected 265, 146 and 442 genes associated with gastroenteritis, hepatitis, and dermatitis, respectively, from Neurocarta (Portales-Casamar *et al*, 2013) and submitted them to MLnet. Among the top 100 proteins predicted to be associated with all three inflammatory diseases, pathways related to the immune response and inflammation are significantly enriched (**Fig S12** and **Table S6**), but not insulin-related or other annotations found enriched among the top 100 common ND modifiers.

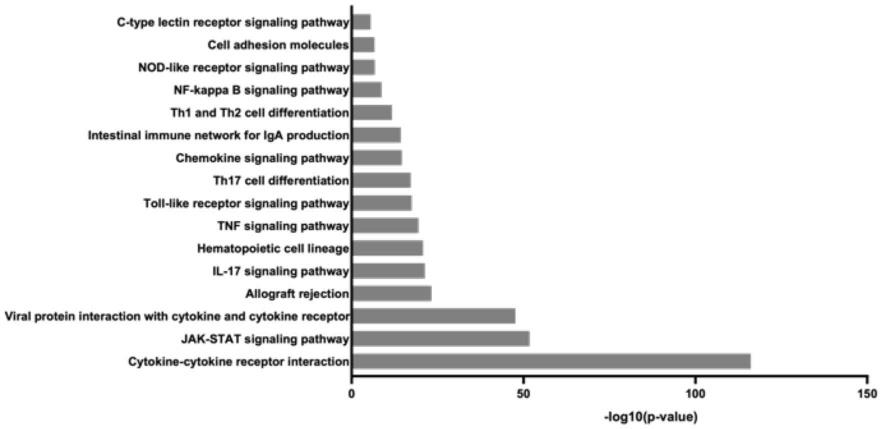

Appendix Figure S12. Pathway enrichment analysis results for genes predicted by MLnet to be commonly associated with gastroenteritis, hepatitis and dermatitis

The disease-related genes and predicted common ones are listed in **Table S6**.

3. To report the MLnet performance results, the authors always refer to the area under the curve (AUC), but they do not specify to which curve they refer. Given the high values reported, I assume it is the AUROC curve, but they should clarify it. If this is correct, ROC curves are seriously affected by data imbalance (i.e. if the number of positives is small, you can get high AUROC values predicting that everything is a negative). The authors should clearly describe the ratio between modulators and non-modulators in every set, and also report individual values of precision and recall (or the AUPRC).

We thank the reviewer for this comment. We changed AUC to AUROC in the manuscript for clarity. The numbers of disease-specific and common modifiers are mentioned in the main text and figures, but we made this clearer in the new submission by adding the total number of modifier genes also to the caption of **Fig 2**. The numbers for the experimentally determined disease-specific genetic modifiers (page 8) are: 113 AD modifiers, 209 HD modifiers, 36 SCA1 modifiers, and 59 SCA3 modifiers. The numbers of common modifiers for different combinations of these diseases are shown in **Fig 2**. For example, there are 113

modifiers for AD and 209 modifiers for HD, of which 10 are common shared between AD and HD (the numbers in parenthesis in **Fig 2**).

Regarding AUPRC, we fully agree with the reviewer and now added a figure with the requested AUPRC values for the predictions made by MLnet, GeneMania, and Endeavour. Though AUPRCs are lower than AUROCs, as expected, due to the small number of common modifiers, MLnet mostly outperforms other methods and specifically predicts common modifiers better when common modifiers across more than two diseases are predicted (**Fig S8**). These new data confirm that MLnet is capable of predicting common modifiers better than other simple gene prioritization methods.

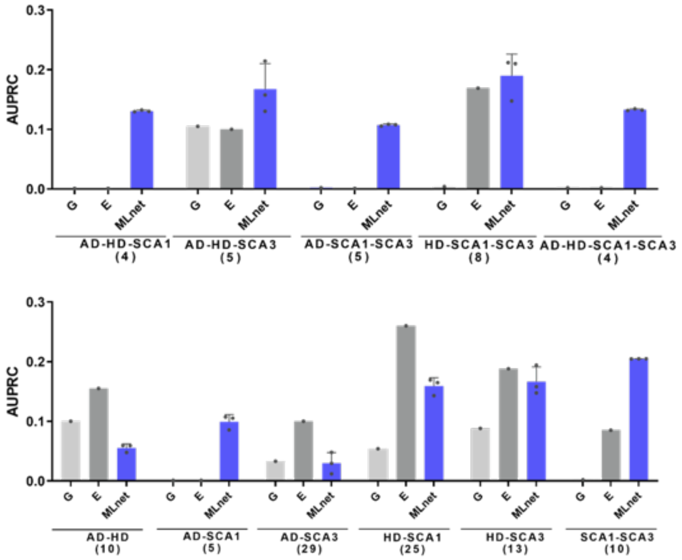

Appendix Figure S8. Comparison of MLnet with other methods using Areas under the Precision-Recall Curve

AUPRCs of MLnet, GeneMania (G), and Endeavour (E). The performances of MLnet in the prediction of modifiers common to different disease groups was assessed using the optimal seed number (100). The number of experimentally identified common modifiers for each group is given in parenthesis.

We also updated the text as follows:

(Page 8)

As shown in **Fig 2**, using the top 100 seeds showed consistently the highest performance (Area Under the Receiver Operating Characteristics, AUROC) in predicting experimentally validated modifiers that are common to different NDs, and, thus, 100 seeds (predicted disease-specific modifiers) were used to run the second module.

(Page 11-12)

To compare MLnet's performance further, we used GeneMania (Warde-Farley *et al*, 2010) and Endeavour (Tranchevent *et al*, 2016). Specifically, we used them to predict disease-associated proteins for each ND and identified the overlapping proteins as common modifiers. The resulting AUROCs are shown in **Fig 2** (*G* and *E* in the graphs). The AUROCs achieved in this way are always lower than those of MLnet. To confirm the advance provided by MLnet further, we also calculated Areas under the Precision-Recall Curve (AUPRC) for the prediction of modifiers common to different disease combinations using the optimal number of seeds (**Fig S8**). AUPRC are lower than AUROC due to the small number of common modifiers. More importantly, MLnet mostly outperforms the other methods, specifically when common modifiers across more than two diseases are predicted. In addition, AUPRCs of GeneMania and Endevour are very low in 4-6 cases, while MLnet shows consistent performances.

(Page 41)

Figure 2. Performances evaluation of MLnet.

The performances of MLnet in the prediction of modifiers common to different disease groups was assessed using different numbers of seed proteins. Diseases were grouped as indicated and common modifiers for that group were predicted with MLnet. As ground truth served the intersection of high-confidence genetic modifiers that were identified experimentally for each disease in that group. The total number of high-confidence modifiers of each ND are: 113 for AD, 209 for HD, 36 for SCA1, and 59 for SCA3. The number of experimentally found common modifiers for each group is given in parenthesis. AUROC was calculated by leave-one-out-cross-validation and the bars are mean ± standard error ($n = 3$). The results for different numbers of seeds are shown as blue bars. As controls, we also assessed the performance of a simple gene prioritization approach (N), GeneMania (G), and Endeavour (E).

4. It is also not clear how the MLnet results compare to just using the common gene set identified for each individual ND. I understand that the network propagation might find more common genes, but it is unclear whether these would indeed be relevant (i.e. real modulators) for each disease. The authors should also compare if the MLnet identified common ND modulators have been reported to be associated to the individual diseases. Without this, it is difficult to assess the added value of the methodology.

We appreciate this comment. There are several aspects to unpack here. There are the disease-specific modifiers that were identified experimentally and those that we predict with the first module of MLnet.

Regarding the experimentally determined ones, we compared them when we assembled the NeuroGeM database (Na et al. 2013) and found very little overlap across multiple NDs. Indeed, we found only four genes that were identified as modifiers across five NDs and these genes are involved in very different cellular processes (some related to proteostasis). One key reason for this limited overlap is that, as alluded to in the response to question 2, the set of genes screened in different experiments for different diseases are quite different and never cover the entire genome. In other words, not all genes have been tested for all NDs, often only a subset of the entire genome. This is one of the main reasons why we developed module 1 of MLnet, which gives a disease-specific modifier score to all genes.

Na D, Rouf M, O'Kane CJ, Rubinsztein DC & Gsponer J (2013) NeuroGeM, a knowledgebase of genetic modifiers in neurodegenerative diseases. BMC Med Genomics 6: 52

Regarding predicted disease-specific modifiers, one can, as suggested by the reviewer, just use a prioritization approach as in module 1 to predict disease-specific modifiers and then search for overlap across diseases. We did that in **Fig 2** (column N). This analysis revealed that this approach is less effective than the network-expansion approach implemented in module 2 of MLnet. To illustrate this aspect further, we traced the disease-specific ranks of the top 12 common modifiers shown in **Fig 4**. As revealed in the table below (**Table S5**), all of these genes are ranked much lower in some of the specific NDs. Thus, taking just the top

ranked 200 genes of each disease and select common ones would not provide the result we achieved with MLnet.

We agree with the reviewer that it is important to provide orthogonal evidence for the validity of predicted common modifiers in each disease; that is why we tested them in the *Drosophila* models. In addition, we now searched the literature for evidence that supports the impact of individual genes in specific diseases (see references for each gene and disease in the table below; **Table S5**). We could find evidence across diseases for many of the genes. However, it needs to be stressed that not all genes may have been assessed for their impact on models of each of the diseases in the focus here. Again, this is a reason why we developed MLnet.

Table S5. Ranks of top 12 predicted common modifiers in predicted disease-specific modifiers and their disease association evidence*

-	AD	HD	SCA1	SCA3
**InR**	**130** [33515428, clinical trials]	**737** [24347322, mouse model]	**89**	**482**
**Akt1**	**113** [34386944, cell and mouse models]	**383** [17984172, fly model]	**15** [12757707, fly model]	**260** [12757707, SCA1 fly model]
**Pdk1**	**59** [36062157, brain tissue]	**431** [14522959, cell model]	**72** [24134140, SCA14 cell model]	**248** [24134140, SCA14 cell model]
**sgg**	**13** [32006534, fly model]	**751** [33753290, cell model]	**145** [27466200, cell model]	**509** [23812869, fly model]
Droj2	**3605** [36448030, yeast and fly models]	**375** [27660272, cell and mouse models]	**82** [9620770, Patient tissue]	**3** [23139745, fly model]
Hsc70Cb	**91** [17309878, fly model]	**14** [27660272, cell and mouse models]	**80** [21625540, cell model]	**48** [20100940, fly model]
Uba1	**30** [17309878, fly model]	**135** [20100940, fly model]	**1149**	**22** [17309878, fly model]
Atg1	**18** [31039426, cell model]	**677** [25723488, cell model]	**169** [23592174, SCA7 cell model]	**199** [23592174, SCA7 cell model]
Mi-2	**88** [21949350, fly model]	**232**	**634**	**78**
par-1	**31** [21949350, fly model]	**557**	**167**	**819**
Lk6	**79**	**608**	**187**	**290**
zip	**386** [26126179, GWAS]	**749** [28900136, yeast model]	**1584**	**2280**

* Predicted ranks and their evidence (PubMed IDs) for disease association are shown. Green color, experimentally discovered modifiers; Grey color, potential association; white color, no evidence.

We updated the text as follows:

(Page 12)
Second, we traced the disease-specific ranks of the top 12 common modifiers predicted by MLnet, which we will discuss and experimentally validate in the following sections. Most of these genes are not ranked in top 200 of at least one of the four NDs (**Table S5**). Thus, taking just the top ranked 200 genes of each disease and select common ones would not provide the result we achieve with MLnet.

(Page 14)

Four of the 12 tested common modifier proteins (Akt1, InR, Pdk1, and sgg (GSK3β)) changed the eye phenotypes in all four ND models when down-regulated by RNAi. Interestingly, three of these (*Akt1*, *InR*, and *Pdk1*) are directly involved in insulin signaling and one of them (*sgg*) acts downstream of the insulin signaling pathway (**Fig 3C**). As a negative control, we also evaluated the effect of two randomly selected low-ranked genes (*Cyp6a18* and *CG34372*) and found them to have little to no impact across the four *Drosophila* ND models (**Fig 4**). To validate the top 12 ranked proteins further, we searched the literature for evidence that supports the impact of them in specific NDs (**Table S5)** and, indeed, we could find evidence across diseases for many of these proteins.

5. The common ND modifiers identified by MLnet are enriched in apoptosis, autophagy and mitophagy. How about the seed modifiers used for each disease? If they are not, this is likely to be an artifact of just identifying very well connected general biological processes. To avoid this potential bias, the authors should also use randomized networks, preserving the connection degree of the proteins and shuffling the edges.

We agree with the reviewer that these terms should appear enriched in at least some of the lists of disease-specific modifiers. If they appear "*de novo*" that would be of concern. We analysed disease-specific modifiers that are generated by module 1. Specifically, we performed KEGG pathway enrichment analysis with the disease-specific modifiers used as seeds (**Fig S10**). As can be seen from the **Fig S10** (see also below), the terms autophagy, mitophagy, and effectors of the insulin pathway (FoxO, mTOR) are all found enriched in at least two diseases. They do not appear in each singe disease, maybe because the corresponding or related genes had not been tested in models for each disease. Importantly, as mentioned in response to point 1 of this reviewer, our central hypothesis is that there is a subset of genetic modifiers **that affect the severity of one of these diseases** that have broader relevance and which may modify several other or even all proteinopathies. In other words, there may be genes of a specific pathway that have been tested positive in models of one disease but for which the relevance in other diseases may not yet be known. We designed MLnet to reweight individual genes so that such candidates are prioritized.

We also agree with the reviewer that a protein interaction network-based approach may suffer from artifacts related to highly connected proteins (hubs). We include a normalization factor into the MLnet calculation to account for protein "hubiness". We show in **Fig S7** that MLnet does not suffer from a strong correlation between prediction rank and protein degree ("hubiness"). It is well established that highly connected and annotated proteins/genes rank

often high in prioritization/gene-prediction approaches, just as a result of their involvement in and connection to numerous cellular processes. Thus, a sort of normalization is necessary. However, one should not *a priori* exclude these highly connected/annotated genes/proteins because they are still relevant to many processes. So, what is the right approach? In the original submission, we tested two methods with the one implemented in MLnet coming out on top. We now followed the reviewer's suggestion and also tested randomized networks for normalization. Specifically, we randomized the interaction network while maintaining interaction degree, used randomized network data for normalization and assessed the ability of this approach to predict experimentally established modifiers common to different disease combinations. As shown in **Fig S6** (see below), though the network randomization approach showed a better performance than seed randomization, MLnet outperformed the other approaches for all disease combinations.

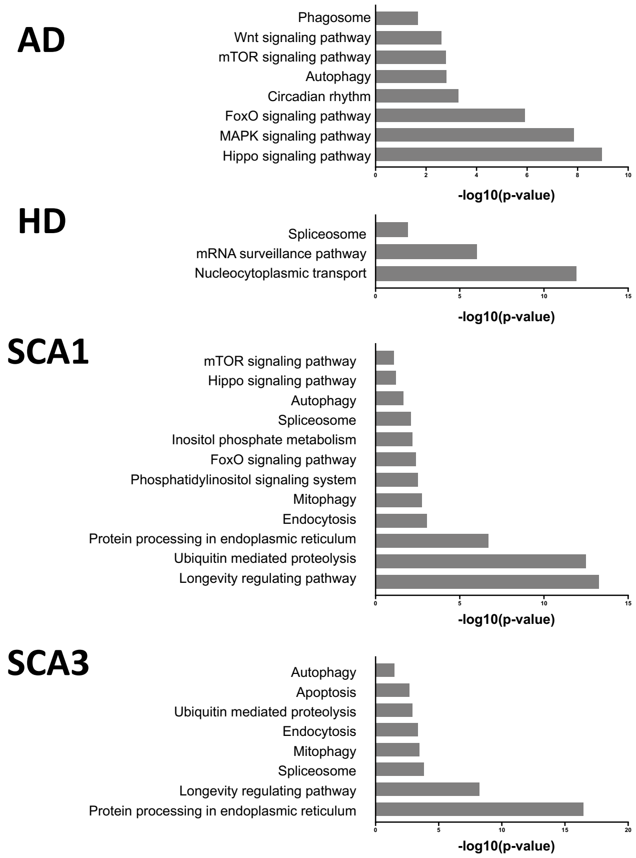

Appendix Figure S10. Pathway enrichment analysis results for the top 100 predicted disease-specific modifiers.

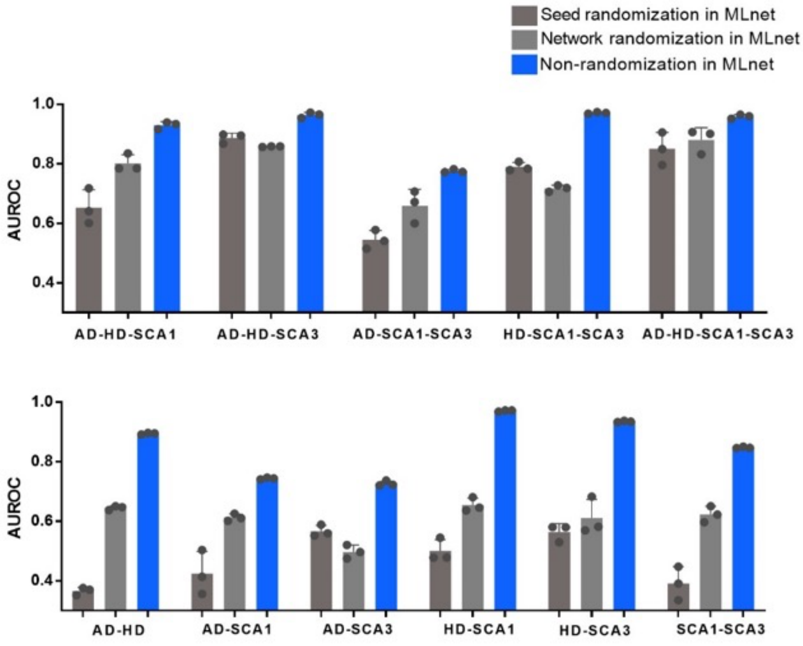

Appendix Figure S6. Performances of MLnet with and without randomization.
The AUROCs of the standard MLnet model (non-randomization), a model in which randomly chosen seeds were used to calculate z-scores (seed randomization), and a model in which randomized networks were used to calculate z-scores (network randomization). For seed randomization approach, seed proteins with the same interaction degree as the query seeds were randomly selected, and z-scores of all proteins in the network were calculated with 10,000 iterations. For network randomization approach, protein-protein interactions were newly linked while maintaining their interaction degrees and z-scores of all proteins in the network were calculated with 10,000 iterations. AUROC was calculated by leave-one-out-cross-validation and the bars are mean ± standard error (*n* = 3).

(Page 13)
The insulin signaling pathway plays a pivotal role in cell survival, cell growth, autophagy, and cytoskeleton organization by regulating downstream factors such as BAD, mTOR, FoxO, and GSK-3β (**Fig 3C**) and has been linked to NDs in numerous studies (Shaughness *et al*, 2020; Akhtar & Sah, 2020; de la Monte & Wands, 2008; Caberlotto *et al*, 2019). Importantly, enrichment in these key annotations does not appear "*de novo*", meaning that annotations related to downstream pathways of insulin signaling, autophagy, and apoptosis are also enriched among predicted disease-specific modifiers (**Fig S10**).

(Page 10)
Robustness analysis in terms of bias toward hub proteins
MLnet uses the number of interactions that a query protein has to normalize the common modifier score *c* (Equation 2) in order to reduce a potential bias toward hub proteins that are more connected in the PPI network. It needs to be stressed that the normalization aims to reduce a potential bias but not prevent hub proteins from being scored high. We tested whether alternative normalizations in module 2 would prevent any heavy bias toward interaction hubs as common modifiers and provide better prediction results. To this end, we modified the MLnet code and calculated *z*-scores for each query protein. For the *z*-score, we randomly selected seed proteins with the same degree as the original seed proteins (predicted disease-specific modifiers) in 10,000 iterations (Seed randomization in **Fig S6**). Alternatively, we randomized the protein-protein interactions while maintaining their interaction degrees (Network randomization in **Fig S6**). As shown in **Fig S6**, the integration of the alternative normalizations using seed randomization or network randomization did not improve performance when using a leave-one-out-cross-validation.

6. 4 out of the 12 potential modulator proteins tested in flies gave a signal. However, it is unclear what is the added value of MLnet in identifying common modulators. How close to the top are these 4 (or 12) proteins in the individual disease models?

We have provided the ranks of the top 12 common modifiers in the table under question 4 of this reviewer. As can be seen, they are generally not top ranked. For example, the rank of Akt1 in SCA1 is 15th, but Akt1 is not included in the top 100 of AD, HD, and SCA3. Furthermore, there are no overlapping modifiers across the top 50 disease-specific modifiers of the four diseases used here (**Fig S9**). For clarity, we newly added the lists of top 100 predicted disease-specific modifiers in **Table S2**.

7. What would be the result if the authors would pick the top proteins in each individual ND and test them in all four fly models?

We see where the reviewer is coming from. The issue here may again be that experimental screens for individual diseases have limited coverage and, thus, disease-specific predictions, based on those screen results, are also limited due to the guilt-by-association approach. The network-based data integration of MLnet is intended to overcome this limitation and "connect" predictions from different diseases. As said, MLnet reweights individual genes so that candidates with higher likelihood of broader relevance are prioritized.

Nevertheless, to test the idea of the reviewer, we carried out a computational experiment. We identified experimentally established modifiers common to different disease combination sets (as done before and shown in **Fig 2**) and then asked how these modifiers are ranked in the other disease(s) not included in the set. As shown below (**Table S4)**, these experimentally established common modifiers of different disease combinations are generally not top ranked in the predicted lists of the other diseases. Thus, common modifiers are not necessarily highly ranked in the disease-specific modifier lists, providing support to the utility of MLnet.

Table S4. Average rank of known common modifiers within predicted disease-specific modifier lists (mean ± standard error).

Disease sets	AD	HD	SCA1	SCA3
AD-HD (10)*	-	-	680 ± 258	627 ± 298
AD-SCA1 (5)	-	2162 ± 1592	-	37 ± 10
AD-SCA3 (29)	-	3041 ± 590	2005 ± 527	-
HD-SCA1 (25)	1058 ± 337	-	-	803 ± 275
HD-SCA3 (13)	532 ± 187	-	827 ± 328	-
SCA1-SCA3 (10)	373 ± 159	1614 ± 1183	-	-
AD-HD-SCA1 (4)	-	-	-	29 ± 9
AD-HD-SCA3 (5)	-	-	248 ± 201	-
AD-SCA1-SCA3 (5)	-	2162 ± 1592	-	-
HD-SCA1-SCA3 (8)	246 ± 125	-	-	-

* Number of known common modifiers of the given disease group

We updated the main text accordingly:
(Page 12)
To investigate the added value of module 2 in common modifier identification further, we carried out additional tests. First, we assessed whether experimentally-identified modifiers common to different sets of disease combinations are highly ranked in the prediction lists of the other diseases (module 1) not included in the set. As shown in **Table S4**, these experimentally established common modifiers of different disease combinations are generally not top ranked in the predicted lists of the other diseases.

8. What about other genes in the pathway not selected as common modifiers, would they show an effect?

We thank the reviewer for this important suggestion. We decided to test a gene in that pathway that allows us also to address part of the third concern of reviewer 2. We selected to test Pi3k92E because it is part of the insulin signaling pathway (**Fig 3C**) and known to mediate insulin's contribution to the regulation of synaptic plasticity. Specifically, we carried out additional experiments in which we knockdown Pi3k92E in the four ND *Drosophila* models (**Fig S13**). Consistent with the other *Drosophila* results, modulation of Pi3k92E resulted in the change in eye phenotype in four NDs, which emphasizes the importance of insulin signaling in NDs as well as the validity of our model.

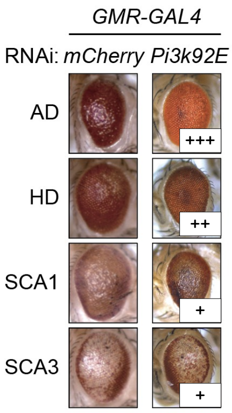

Appendix Figure S13. Effect of Pi3K92E knockdown on *Drosophila* eye phenotype in four NDs

(Page 15)

Given the positive testing of all four genes related to the insulin pathway, we decided to assess the impact of another insulin pathway protein on the disease phenotypes. We evaluated Pi3K92E (PI3K in **Fig 3C**) because it is within the top 20 of the predicted common modifiers. Moreover, PI3K is of particular interest because it is one of the key mediators of the insulin pathway's impact on brain plasticity and neurogenesis. For instance, PI3-kinase is essential for glutamate receptor insertion at plasma membranes during synaptic plasticity (Man *et al*, 2003). Downregulation of Pi3k92E changed the eye phenotype in all four ND models (**Fig S13**), confirming the significance of insulin signaling for the model phenotypes investigated here.

9. I do not really understand the experiment in HEK293 cells. Why are cells expressing ND-related genes expected to be less viable? Specific ND-related genes do not affect cell viability

(and certainly not in HEK293 cells). The authors should clarify this experiment and the interpretation of the results.

We may not have made that clear enough in the initial submission. We agree that these cells are not affected by the wild-type genes. However, it does not mean that the viability of HEK293 is not affected by mutated genes. Cell viability can be affected by the aggregation of the gene product. The likelihood of aggregation can be enhanced via mutations (e.g., the repeats of polyQ in HD, SCA1, and SCA3-related genes, or aggregation-promoting mutations of Aβ for AD models) as well as protein expression levels. As shown in **Figs 5B-E**, the viability of HEK293 is in the range of 60 - 90% when mutated ND genes are expressed. If a mutant gene is expressed at a higher level or a more aggregation-prone mutant gene is expressed, the viability would decrease accordingly. Thus, cell viability assays using HEK293 have been used to study disease processes and to test potential drugs to treat NDs (Hart et al, 2022; Niu et al, 2022; Pierzynowska et al, 2018; Shentu et al, 2019; Wang et al, 2006, 2019). We revised our manuscript (page 16).

References:

Hart, H. S. et al. The cytoprotective role of GM1 ganglioside in Huntington disease cells. Mol Biol Rep 49, 12253–12258 (2022).
Niu, T.-T. et al. Protective Effects of Ginkgolide on a Cellular Model of Alzheimer's Disease via Suppression of the NF-κB Signaling Pathway. Appl Biochem Biotechnol 194, 2448–2464 (2022).
Pierzynowska, K. et al. Correction of Huntington's Disease Phenotype by Genistein-Induced Autophagy in the Cellular Model. Neuromolecular Med 20, 112–123 (2018).
Shentu, Y.-P. et al. Genistein Decreases APP/tau Phosphorylation and Ameliorates Aβ Overproduction Through Inhibiting CIP2A. Curr Alzheimer Res 16, 732–740 (2019).
Wang, C.-F. et. al. Protective effects of melatonin on mitochondrial biogenesis and mitochondrial structure and function in the HEK293-APPswe cell model of Alzheimer's disease. Eur Rev Med Pharmacol Sci 23 (8): 3542-3550 (2019)
Wang, H. et al. Suppression of polyglutamine-induced toxicity in cell and animal models of Huntington's disease by ubiquilin. Hum Mol Genet 15, 1025–1041 (2006).

We updated the main text to make this point clearer

(Page 16)

In a first test, we constructed human cell-based models for AD, HD, SCA1, and SCA3 by expressing ND-causing genes ($Aβ_{1-42}$, Htt-Q74, Atx1-Q52, and Atx3-Q84) in HEK293 cells and evaluated the impact of Akt1 activation on cell phenotypes. HEK293 are generally not affected by the wild-type forms of the disease genes. However, their viability is affected by the gene products of variants that have an increased likelihood of aggregation (e.g., polyQ repeats in HD, SCA1, and SCA3-related genes), especially when expressed at high levels. Thus, cell viability assays with HEK293 cells have extensively been used to study disease mechanisms and test small compounds for their impact on aggregation and cell viability (Bartley *et al*, 2012; Hart *et al*, 2022; Niu *et al*, 2022; Shentu *et al*, 2019; Wang *et al*, 2006, 2019; Pierzynowska *et al*, 2018).

10. In the last section, the authors kind of drop all the previous results and only focus on testing the effect of the insulin pathway in Alzheimer´s disease which, as they state, has been clearly established! I fail to see the added value of re-reporting that the modulation of the insulin pathway has an effect on the 5xFAD mouse model. If anything, they should test the effect of this modulation of models other that AD (i.e. HD or the SCs).

We appreciate this pertinent objection by the reviewer.

We tested the activation of Akt1 initially in four ND cell models (**Fig 5B-E**). As our resources are limited, we were not able to test activation of Akt1 in mice models of all four NDs in the focus here, which would be ideal. We decided to go for AD models for two main reasons. (1) AD is the ND that is the most common among the four studied here and the one with the biggest socio-economical burden. (2) We agree that the importance of the insulin pathway is much better elucidated in Alzheimer than in the other ND pathologies. However, scientific results can be conflicting and not always easily reproducible. For example, treatment for restoring the insulin and PI3K-Akt signaling pathways, which is reduced in AD patients, showed mixed results in clinical trials. Specifically, as this reviewer is certainly aware of, administration of insulin to healthy individuals and AD patients improved memory function in small-scale studies, but in large scale clinical trials the results were mixed (Hallschmid, 2021; Morris & Burns, 2012). Furthermore, GSK3β inhibitors showed positive improvement in animal models, but not in clinical studies (Rippin & Eldar-Finkelman, 2021; Arciniegas Ruiz & Eldar-Finkelman, 2022). These contradictory results might be due to the fact that insulin is involved in a variety of cellular processes including cell growth, survival, and metabolism, as well as a variety of tissues including the brain and pancreatic tissues. Thus, we wanted to test insulin activation in a mouse AD model by ourselves because further clarification in this area is needed. In addition, previous studies revealed that activated Akt1 level are decreased in mouse AD models, but its level is up-regulated in AD patients (Griffin et al., 2005; Kommaddi et al., 2023; Rickle et al., 2004). Therefore, we wanted to test the effect of an Akt1 activator.

In hindside, we agree that testing in a HD mouse model may have been more impactful. This said, the focus of this paper is on the introduction of a novel computational method, and we already validate predictions in *Drosophila* and cell models of four NDs.

References:

Arciniegas Ruiz SM & Eldar-Finkelman H (2022) Glycogen synthase kinase-3 inhibitors: Preclinical and clinical focus on CNS-A decade onward. Front Mol Neurosci 14: 792364

Griffin RJ, Moloney A, Kelliher M, Johnston JA, Ravid R, Dockery P, O'Connor R & O'Neill C (2005) Activation of Akt/PKB, increased phosphorylation of Akt substrates and loss and altered distribution of Akt and PTEN are features of Alzheimer's disease pathology. Journal of Neurochemistry 93: 105–117

Hallschmid M (2021) Intranasal insulin for Alzheimer's disease. CNS Drugs 35: 21–37

Morris JK & Burns JM (2012) Insulin: An emerging treatment for Alzheimer's disease dementia? Curr Neurol Neurosci Rep 12: 520–527

Kommaddi RP, Gowaikar R, Singh K, P A H & Ravindranath V (2023) Down regulation of Akt/mTOR signaling pathway proteins in hippocampus of Alzheimer's disease mouse model. Alzheimer's & Dementia 19: e064101

Rickle A, Bogdanovic N, Volkman I, Winblad B, Ravid R & Cowburn RF (2004) Akt activity in Alzheimer's disease and other neurodegenerative disorders. Neuroreport 15: 955–959

Rippin I & Eldar-Finkelman H (2021) Mechanisms and therapeutic implications of GSK-3 in treating neurodegeneration. Cells 10: 262

11. The authors also say that GSK3b is the only identified AD-HD modifier not directly involved in the insulin pathway, but there is an infinity of literature relating GSK3b and insulin levels. Moreover, GSK3b inhibitors have been extensively tested to treat AD, showing very promising results in mice models, but failing the clinical trials.

We thank the reviewer for these clarifying remarks. We were not expressing things clearly in our initial submission. We have now clarified these points and highlight that GSK3β is involved in the extended insulin signaling pathway as shown in **Fig 3C (see below)**. We now discuss that insulin and GSK3b inhibitors have been tested to treat AD but failed or showed mixed results in large clinical trials (see below).

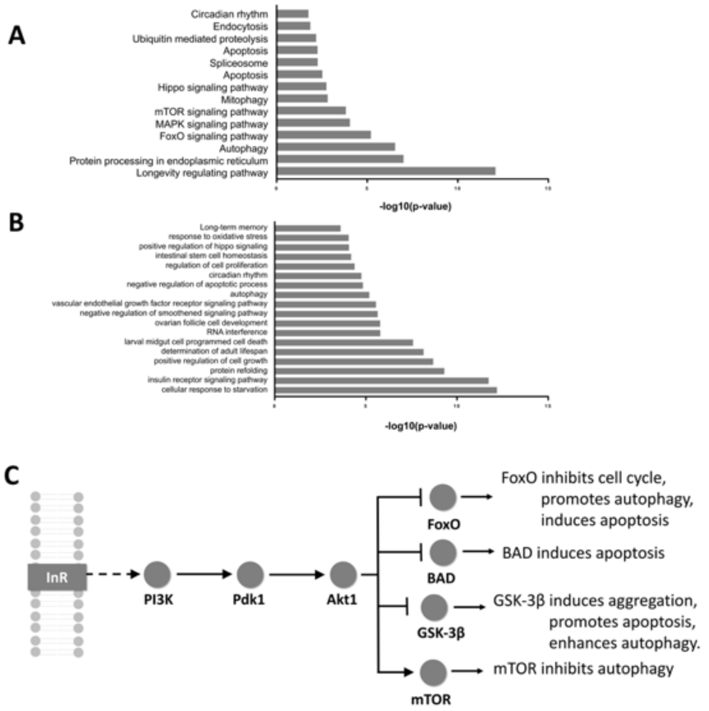

Figure 3. **KEGG pathway and GO enrichment analyses with predicted common modifiers**.

A KEGG pathway enrichment analysis of the top 100 predicted common modifiers.

B GO enrichment analysis of the top 100 predicted common modifiers.

C A simplified schematic diagram of the insulin signaling pathway and downstream functions.

(Page 14)
Four of the 12 tested common modifier proteins (Akt1, InR, Pdk1, and sgg (GSK3β)) changed the eye phenotypes in all four ND models. Interestingly, three of these (*Akt1*, *InR*, and *Pdk1*) are directly involved in insulin signaling and one of them (*sgg*) acts downstream of the insulin signaling pathway (**Fig 3C**).

(Page 20-21)

Moreover, although our experiments suggest a modulatory role of Akt1 for multiple NDs, it may not be an ideal target for ND treatment development because of its involvement in numerous cellular processes and the fact that its enhanced activation can lead to cancerous cell transformation (Wang *et al*, 2017), which would require very close monitoring for tumorigenic effects when activated via a therapeutic agent. Other proteins in the insulin pathway such as the downstream effector GSK3β are already actively targeted for ND therapy development. GSK3β inhibitors showed positive improvement in animal models, but unfortunately failed in AD patients (Rippin & Eldar-Finkelman, 2021; Arciniegas Ruiz & Eldar-Finkelman, 2022). Moreover, direct insulin administration to healthy individuals and AD patients improved memory performance in small studies, but mixed results were reported for larger clinical trials (Hallschmid, 2021; Morris & Burns, 2012). It is clear that more research is required to fully understand the roles of insulin signaling in NDs and whether activation of specific elements of this signaling pathway may benefit patients.

12. Finally, the number of animals used in the Barnes maze test (4/5 mice) seems insufficient. The learning curve for the AD model is not really consistent in the days 3,4 and 5. The same effect is observed in the test days (15 and 16). Although, Fig 5G shows a mild significance when comparing days 5 and 16 between AD and AD+SC79, this difference should be supported by a robust learning curve, or any small change (i.e. adding one more animal or changing the days compared) could wipe it out.

We thank the reviewer for the comment. We have tested another 16 mice (four mice of WT, five mice of WT + SC79, three mice of AD, and four mice of AD + SC79) after submitting our manuscript and have added now the new results to **Figs 5F-H** (please see below). Though there are variations in the results because of the intrinsic variations of animal behavior, trends are confirmed and *p*-values still indicate statistical significance (p-value < 0.05). Importantly, the learning curves at days 3,4 and 5 are much more consistent now.

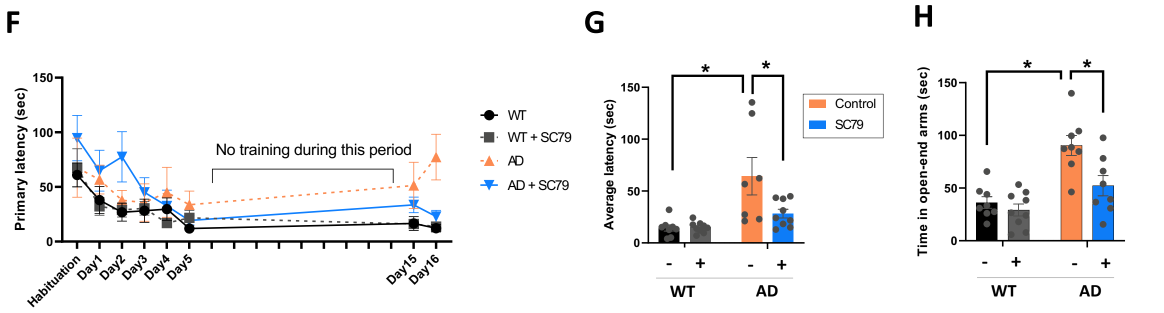

23rd Oct 2023

Manuscript Number: MSB-2023-11801R
Title: Multi-layered network expansion model identifies Akt1 as common modulator of neurodegeneration

Dear Dr Gsponer,

Thank you for sending us your revised manuscript. We have now heard back from the two reviewers who agreed to evaluate your revised study. As you will see below, the reviewers are satisfied with the performed revisions and support publication. As such, I am glad to inform you that we can soon accept your manuscript for publication, pending some minor revisions listed below, all related to editorial issues.

- The funding information (NRF-2022M3A9B6082687, and NRF-2018R1A5A1025077; 2021R1A2C1006353; Korea University grant; CIHR) needs to be entered in the submission system.

- Please indicate the statistical test used for data analysis in the legends of Figures 3a-b.

- The "Tool Availability" section should be renamed to "Data Availability". Full source codes etc. should be made available on GitHub.

- Tables S1-S8 should be uploaded individually (one xls file per Dataset) as EV Datasets EV1-EV8. The callouts in the text should be corrected accordingly. Please include a description of each dataset in the xls file as a separate sheet.

- Please include callouts for Appendix Figure S14 and Datasets EV7-EV8 in the main text.

- The callouts to Appendix Figures should be Appendix Figure S1, Appendix Figure S2 etc. (instead of Fig S1, Fig S2 etc.).

- Please make sure that no text remains labelled in red in the Appendix. The Appendix will appear online as it is provided (it is not going to be typeset).

- The source data files need to be reorganized. Please provide one file (or zip folder) per main figure. A single ZIP folder should be provided for all the source data for the Appendix figures.

- The synopsis image does not display well at the final size required. Please resupply the image as a jpg or png at the required final size (it needs to be exactly 550 px wide, and the height ideally < 500 px), ensuring that all labels are legible. Reorganizing the image so that not all image elements are displayed on "one line" might work better.

Please resubmit your revised manuscript online, with a covering letter listing amendments and responses to each point raised by the referees. Please resubmit the paper **within one month** and ideally as soon as possible. If we do not receive the revised manuscript within this time period, the file might be closed and any subsequent resubmission would be treated as a new manuscript. Please use the Manuscript Number (above) in all correspondence.

Click on the link below to submit your revised paper.

https://msb.msubmit.net/cgi-bin/main.plex

As a matter of course, please make sure that you have correctly followed the instructions for authors as given on the submission website.

Thank you for submitting this paper to Molecular Systems Biology.

Kind regards,

Maria

Maria Polychronidou, PhD
Senior Editor
Molecular Systems Biology

If you do choose to resubmit, please click on the link below to submit the revision online before 22nd Nov 2023.

https://msb.msubmit.net/cgi-bin/main.plex

IMPORTANT:
Please note that corresponding authors are required to supply an ORCID ID for their name upon submission of a revised manuscript (EMBO Press signed a joint statement to encourage ORCID adoption).
(https://www.embopress.org/page/journal/17444292/authorguide#editorialprocess)
Currently, our records indicate that the ORCID for your account is 0000-0003-3672-6594.

Please click the link below to modify this ORCID:
Link Not Available

The system will prompt you to fill in your funding and payment information. This will allow Wiley to send you a quote for the article processing charge (APC) in case of acceptance. This quote takes into account any reduction or fee waivers that you may be eligible for. Authors do not need to pay any fees before their manuscript is accepted and transferred to the publisher.

*** PLEASE NOTE *** As part of the EMBO Press transparent editorial process initiative (see our Editorial at https://dx.doi.org/10.1038/msb.2010.72 , Molecular Systems Biology will publish online a Review Process File to accompany accepted manuscripts. When preparing your letter of response, please be aware that in the event of acceptance, your cover letter/point-by-point document will be included as part of this File, which will be available to the scientific community. More information about this initiative is available in our Instructions to Authors. If you have any questions about this initiative, please contact the editorial office (msb@embo.org).

Reviewer #2:

The authors have satisfactorily addressed my major concerns.

Suggest correction to the sentence:
A multi-layered network expansion model is introduced that finds (replace with "to find"? or rewrite) proteins with pathophysiological relevance for groups of diseases.

Reviewer #3:

I was not at all convinced by the first version of the m/s. However, in my opinion, the authors have done a great job during revision, addressing all the comments raised by the Reviewers. My opinion is tat now the m/s is ready for publication. I am still not convinced of some of the points/decision made by the authors, but they are all now properly discussed and should be clear to the reader.

Finally, I would like to thank the authors for their effort in tackling all the points raised.

All editorial and formatting issues were resolved by the authors.

27th Oct 2023

Manuscript number: MSB-2023-11801RR
Title: Multi-layered network expansion model identifies Akt1 as common modulator of neurodegeneration

Dear Dr Gsponer,

Thank you again for sending us your revised manuscript. We are now satisfied with the modifications made and I am pleased to inform you that your paper has been accepted for publication.

*** PLEASE NOTE *** As part of the EMBO Publications transparent editorial process initiative (see our Editorial at https://dx.doi.org/10.103/msb.2010.72), Molecular Systems Biology publishes online a Review Process File with each accepted manuscripts. This file will be published in conjunction with your paper and will include the anonymous referee reports, your point- by-point response and all pertinent correspondence relating to the manuscript. If you do NOT want this File to be published, please inform the editorial office at msb@embo.org within 14 days upon receipt of the present letter.

LICENSE AND PAYMENT:
All articles published in Molecular Systems Biology are fully open access: immediately and freely available to read, download and share.

Molecular Systems Biology charges an article processing charge (APC) to cover the publication costs. You, as the corresponding author for this manuscript, should have already received a quote with the article processing fee separately. Please let us know in case this quote has not been received.

Once your article is at Wiley for editorial production, you will receive an email from Wiley's Author Services system, which will ask you to log in and will present you with the publication license form for completion. Within the same system the publication fee can be paid by credit card, an invoice or pro forma can be requested.

Payment of the publication charge and the signed Open Access Agreement form must be received before the article can be published online.

Upon acceptance it is mandatory for you to return the completed payment form. Failure to send back the form may result in a delay in the publication of your paper.

Molecular Systems Biology articles are published under the Creative Commons licence CC BY, which facilitates the sharing of scientific information by reducing legal barriers, while mandating attribution of the source in accordance to standard scholarly practice.

Proofs will be forwarded to you within the next 2-3 weeks.

Thank you very much for submitting your work to Molecular Systems Biology.

Kind regards,

Maria

Maria Polychronidou, PhD
Senior Editor
Molecular Systems Biology

EMBO Press Author Checklist

Corresponding Author Name: Jörg Gsponer
Journal Submitted to: Molecular Systems Biology
Manuscript Number: MSB-2023-11801

USEFUL LINKS FOR COMPLETING THIS FORM
The EMBO Journal - Author Guidelines
EMBO Reports - Author Guidelines
Molecular Systems Biology - Author Guidelines
EMBO Molecular Medicine - Author Guidelines

Reporting Checklist for Life Science Articles (updated January
This checklist is adapted from Materials Design Analysis Reporting (MDAR) Checklist for Authors. MDAR establishes a minimum set of requirements in transparent reporting in the life sciences (see Statement of Task: 10.31222/osf.io/9sm4x). Please follow the journal's guidelines in preparing your
Please note that a copy of this checklist will be published alongside your article.

Abridged guidelines for figures
1. Data
The data shown in figures should satisfy the following conditions:
- the data were obtained and processed according to the field's best practice and are presented to reflect the results of the experiments in an accurate and unbiased manner.
- ideally, figure panels should include only measurements that are directly comparable to each other and obtained with the same assay.
- plots include clearly labeled error bars for independent experiments and sample sizes. Unless justified, error bars should not be shown for technical
- if n<5, the individual data points from each experiment should be plotted. Any statistical test employed should be justified.
- Source Data should be included to report the data underlying figures according to the guidelines set out in the authorship guidelines on Data

2. Captions
Each figure caption should contain the following information, for each panel where they are relevant:
- a specification of the experimental system investigated (eg cell line, species name).
- the assay(s) and method(s) used to carry out the reported observations and measurements.
- an explicit mention of the biological and chemical entity(ies) that are being measured.
- an explicit mention of the biological and chemical entity(ies) that are altered/varied/perturbed in a controlled manner.
- the exact sample size (n) for each experimental group/condition, given as a number, not a range;
- a description of the sample collection allowing the reader to understand whether the samples represent technical or biological replicates (including how many animals, litters, cultures, etc.).
- a statement of how many times the experiment shown was independently replicated in the laboratory.
- definitions of statistical methods and measures:
  - common tests, such as t-test (please specify whether paired vs. unpaired), simple χ2 tests, Wilcoxon and Mann-Whitney tests, can be unambiguously identified by name only, but more complex techniques should be described in the methods section;
  - are tests one-sided or two-sided?
  - are there adjustments for multiple comparisons?
  - exact statistical test results, e.g., P values = x but not P values < x;
  - definition of 'center values' as median or average;
  - definition of error bars as s.d. or s.e.m.

Please complete ALL of the questions below.
Select "Not Applicable" only when the requested information is not relevant for your study.

Materials

Newly Created Materials	Information included in the manuscript?	In which section is the information available? (Reagents and Tools Table, Materials and Methods, Figures, Data Availability Section)
New materials and reagents need to be available; do any restrictions apply?	Not Applicable	

Antibodies	Information included in the manuscript?	In which section is the information available? (Reagents and Tools Table, Materials and Methods, Figures, Data Availability Section)
For **antibodies** provide the following information:<br>- Commercial antibodies: RRID (if possible) or supplier name, catalogue number and or/clone number<br>- Non-commercial: RRID or citation	Not Applicable	

DNA and RNA sequences	Information included in the manuscript?	In which section is the information available? (Reagents and Tools Table, Materials and Methods, Figures, Data Availability Section)
**Short novel DNA or RNA including primers, probes**: provide the sequences.	Not Applicable	

Cell materials	Information included in the manuscript?	In which section is the information available? (Reagents and Tools Table, Materials and Methods, Figures, Data Availability Section)
**Cell lines:** Provide species information, strain. Provide accession number in repository **OR** supplier name, catalog number, clone number, and/**OR** RRID.	Yes	Reagents and Tools Table
**Primary cultures:** Provide species, strain, sex of origin, genetic modification status.	Yes	Reagents and Tools Table
Report if the cell lines were recently **authenticated** (e.g., by STR profiling) and tested for mycoplasma contamination.	Not Applicable	

Experimental animals	Information included in the manuscript?	In which section is the information available? (Reagents and Tools Table, Materials and Methods, Figures, Data Availability Section)
**Laboratory animals or Model organisms:** Provide species, strain, sex, age, genetic modification status. Provide accession number in repository **OR** supplier name, catalog number, clone number, **OR** RRID.	Yes	Reagents and Tools Table
**Animal observed in or captured from the field:** Provide species, sex, and age where possible**.**	Not Applicable	
Please detail **housing and husbandry conditions**.	Yes	Materials and Methods

Plants and microbes	Information included in the manuscript?	In which section is the information available? (Reagents and Tools Table, Materials and Methods, Figures, Data Availability Section)
**Plants:** provide species and strain, ecotype and cultivar where relevant, unique accession number if available, and source (including location for collected wild specimens).	Not Applicable	
**Microbes:** provide species and strain, unique accession number if available, and source.	Not Applicable	

Human research participants	Information included in the manuscript?	In which section is the information available? (Reagents and Tools Table, Materials and Methods, Figures, Data Availability Section)
If collected and within the bounds of privacy constraints report on age, sex and gender or ethnicity for all study participants.	Not Applicable	

Core facilities	Information included in the manuscript?	In which section is the information available? (Reagents and Tools Table, Materials and Methods, Figures, Data Availability Section)
If your work benefited from core facilities, was their service mentioned in the acknowledgments section?	Not Applicable	

Design

Study protocol	Information included in the manuscript?	In which section is the information available? (Reagents and Tools Table, Materials and Methods, Figures, Data Availability Section)
If study protocol has been **pre-registered, provide DOI in the manuscript**. For clinical trials, provide the trial registration number **OR** cite DOI.	Not Applicable	
Report the **clinical trial registration number** (at ClinicalTrials.gov or equivalent), where applicable.	Not Applicable	

Laboratory protocol	Information included in the manuscript?	In which section is the information available? (Reagents and Tools Table, Materials and Methods, Figures, Data Availability Section)
Provide DOI OR other citation details if **external detailed step-by-step protocols** are available.	Not Applicable	

Experimental study design and statistics	Information included in the manuscript?	In which section is the information available? (Reagents and Tools Table, Materials and Methods, Figures, Data Availability Section)
Include a statement about **sample size** estimate even if no statistical methods were used.	Yes	Materials and Methods, Figures
Were any steps taken to minimize the effects of subjective bias when allocating animals/samples to treatment (e.g. **randomization procedure**)? If yes, have they been described?	Yes	Materials and Methods
Include a statement about **blinding** even if no blinding was done.	Yes	Materials and Methods
Describe **inclusion/exclusion criteria** if samples or animals were excluded from the analysis. Were the criteria pre-established?<br><br>If sample or data points were omitted from analysis, report if this was due to attrition or intentional exclusion and provide justification.	Not Applicable	
For every figure, are **statistical tests** justified as appropriate? Do the data meet the assumptions of the tests (e.g., normal distribution)? Describe any methods used to assess it. Is there an estimate of variation within each group of data? Is the variance similar between the groups that are being statistically compared?	Yes	Figures

Sample definition and in-laboratory replication	Information included in the manuscript?	In which section is the information available? (Reagents and Tools Table, Materials and Methods, Figures, Data Availability Section)
In the figure legends: state number of times the experiment was **replicated** in laboratory.	Yes	Figures
In the figure legends: define whether data describe **technical or biological replicates**.	Yes	Figures

Ethics

Ethics	Information included in the manuscript?	In which section is the information available? (Reagents and Tools Table, Materials and Methods, Figures, Data Availability Section)
Studies involving **human participants**: State details of **authority granting ethics approval** (IRB or equivalent committee(s), provide reference number for approval.	Not Applicable	
Studies involving **human participants**: Include a statement confirming that **informed consent** was obtained from all subjects and that the experiments conformed to the principles set out in the WMA Declaration of Helsinki and the Department of Health and Human Services Belmont Report.	Not Applicable	
Studies involving **human participants:** For publication of **patient photos**, include a statement confirming that consent to publish was obtained.	Not Applicable	
Studies involving experimental **animals**: State details of **authority granting ethics approval** (IRB or equivalent committee(s), provide reference number for approval. Include a statement of compliance with ethical regulations.	Yes	Materials and Methods
Studies involving **specimen and field samples:** State if relevant **permits** obtained, provide details of authority approving study; if none were required, explain why.	Not Applicable	

Dual Use Research of Concern (DURC)	Information included in the manuscript?	In which section is the information available? (Reagents and Tools Table, Materials and Methods, Figures, Data Availability Section)
Could your study fall under dual use research restrictions? Please check biosecurity documents and list of **select agents and toxins** (CDC): https://www.selectagents.gov/sat/list.htm	Not Applicable	
If you used a select agent, is the security level of the lab appropriate and reported in the manuscript?	Not Applicable	
If a study is subject to dual use research of concern regulations, is the name of the **authority granting approval and reference number** for the regulatory approval provided in the manuscript?	Not Applicable	

Reporting

The MDAR framework recommends adoption of discipline-specific guidelines, established and endorsed through community initiatives. Journals have their own policy about requiring specific guidelines and recommendations to complement MDAR.

Adherence to community standards	Information included in the manuscript?	In which section is the information available? (Reagents and Tools Table, Materials and Methods, Figures, Data Availability Section)
State if relevant guidelines or checklists (e.g., **ICMJE, MIBBI, ARRIVE, PRISMA**) have been followed or provided.	Not Applicable	
For **tumor marker prognostic studies,** we recommend that you follow the **REMARK** reporting guidelines (see link list at top right). See author guidelines, under 'Reporting Guidelines'. Please confirm you have followed these guidelines.	Not Applicable	
For **phase II and III randomized controlled trials**, please refer to the **CONSORT** flow diagram (see link list at top right) and submit the CONSORT checklist (see link list at top right) with your submission. See author guidelines, under 'Reporting Guidelines'. Please confirm you have submitted this list.	Not Applicable	

Data Availability

Data availability	Information included in the manuscript?	In which section is the information available? (Reagents and Tools Table, Materials and Methods, Figures, Data Availability Section)
Have **primary datasets** been deposited according to the journal's guidelines (see 'Data Deposition' section) and the respective accession numbers provided in the Data Availability Section?	Yes	Data Availability
Were **human clinical and genomic datasets** deposited in a public access-controlled repository in accordance to ethical obligations to the patients and to the applicable consent agreement?	Not Applicable	
Are **computational models** that are central and integral to a study available without restrictions in a machine-readable form? Were the relevant accession numbers or links provided?	Yes	Data Availability
If publicly available data were reused, provide the respective **data citations in the reference list**.	Yes	Reagents and Tools Table
